# eNOS uncoupling drives oxidative stress and arrhythmogenic vulnerability in experimental hypothyroidism

**DOI:** 10.1016/j.redox.2026.104447

**Published:** 2026-10-20

**Authors:** Diego Santos Souza, Leisiane Pereira Marques, Aimée Obolari Durço, Fabiana da Silva Alcântara, Diego José Belato Orts, Melissa Tainan Silva Dias, Michael Ramon de Lima Conceiçao, Jorge Lucas Teixeira-Fonseca, Luiz Fernando Santos Matos, Maria Luiza Alarcon Mady Barbosa, Daiara Custódio Santos, Luciano dos Santos Aggum Capettini, Carlos Roberto Prudencio, Danilo Lustrino, Jader Santos Cruz, Danilo Roman-Campos

**Affiliations:** a Department of Physiology, Federal University of Sergipe, São Cristóvão, Brazil; b Department of Biophysics, Federal University of São Paulo, São Paulo, Brazil; c Department of Pharmacology, Federal University of Minas Gerais, Belo Horizonte, Brazil; d Immunology Center, Institute Adolfo Lutz, São Paulo, Brazil; e Department of Biochemistry and Immunology, Federal University of Minas Gerais, Belo Horizonte, Brazil

**Keywords:** Cardiac arrhythmia, Oxidative stress, eNOS uncoupling, Hypothyroidism, Redox signaling

## Abstract

**Aims:**

Cardiac arrhythmias are a major cause of morbidity and sudden cardiac death worldwide, and hypothyroidism has emerged as an important risk factor. Among the mechanisms involved in cardiac electrical stability, nitric oxide (NO) signaling, particularly via endothelial nitric oxide synthase (eNOS), plays a critical role in cardiac excitation–contraction coupling. Here, we investigated whether hypothyroidism increases susceptibility to stress-induced arrhythmias through eNOS uncoupling and excessive reactive oxygen species (ROS) production.

**Methods and results:**

Hypothyroidism was induced in Swiss mice by methimazole administration in the drinking water for 21 days. *In vivo* electrocardiographic recordings revealed a marked increase in stress-induced arrhythmias following dobutamine/caffeine challenge. At the cellular level, left ventricular cardiomyocytes from hypothyroid mice exhibited elevated ROS production, electrical instability, enhanced Na^+^ and Ca^2+^ currents, and reduced repolarizing K^+^ currents, resulting in action potential prolongation. Unbalanced oxidative stress was associated with redox-dependent Ca²⁺/calmodulin-dependent protein kinase II (CaMKII) activation, contributing to intracellular Ca^2+^ instability and triggered activity in cardiomyocytes. Mechanistically, hypothyroidism promotedeNOS uncoupling in cardiomyocytes, evidenced by a reduced eNOS dimer/monomer ratio, leading to increasedROSgeneration and impaired NO production. Pharmacological restoration of eNOS coupling with tetrahydrobiopterin (BH4) normalized ROS and NO at the cellular level, attenuated redox-dependent CaMKII activation, and significantly reduced arrhythmia incidence *in vivo*.

**Conclusion:**

Together, these findings demonstrate that eNOS uncoupling is a key driver of oxidative stress and arrhythmogenic vulnerability in experimental hypothyroidism, suggesting that restoration of eNOS coupling may represent a potential therapeutic strategy to reduce arrhythmogenic risk in hypothyroidism.

## Graphical abstract

**Figure:**
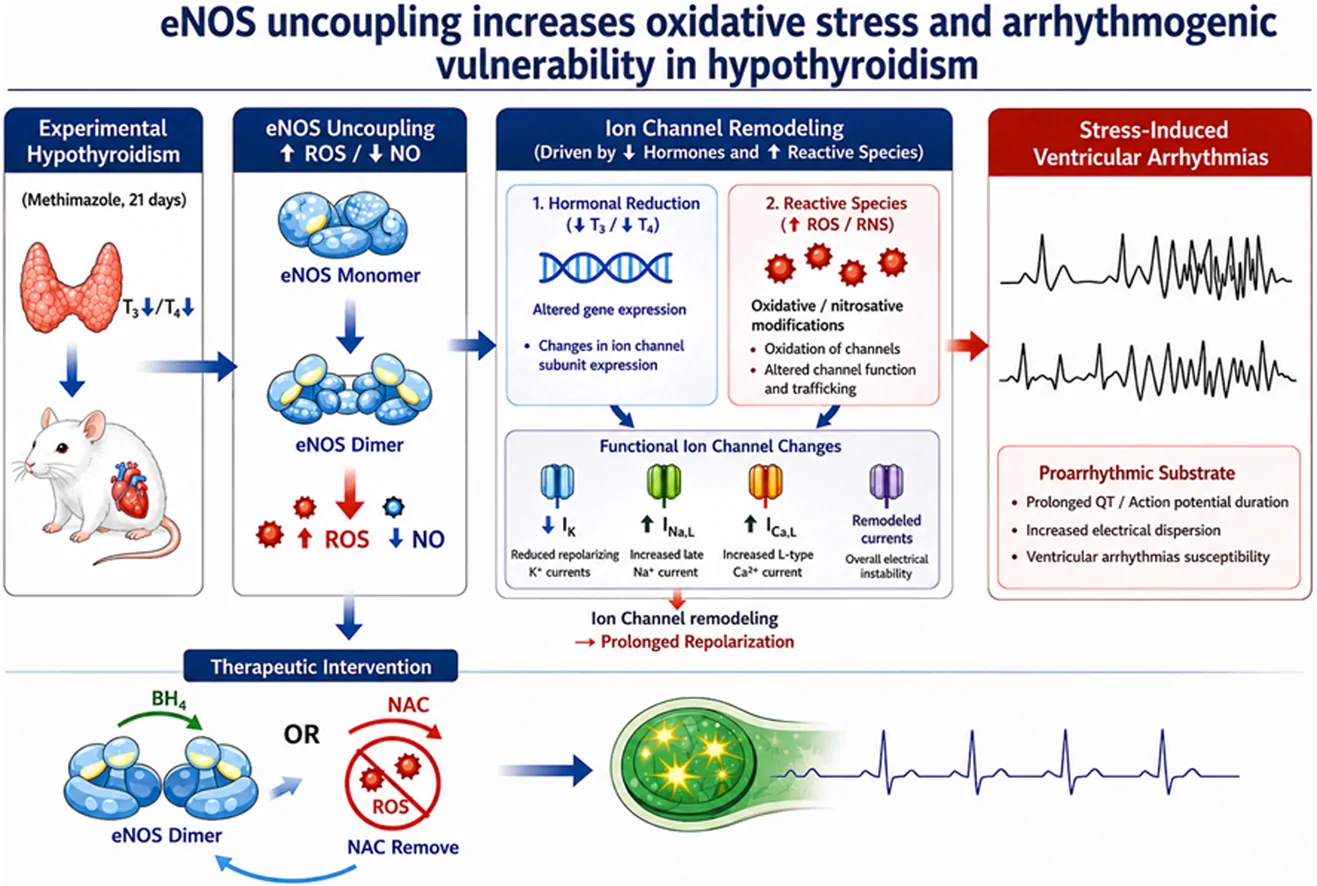

## 1 Introduction

Thyroid hormones (THs) are fundamental regulators of cardiac transcription [1], controlling the expression of key myocardial genes involved in ion channel function, metabolism, and intracellular calcium handling, thereby governing heart rate and excitation-contraction coupling (ECC) [2]. In hypothyroidism (Hypo), this regulation is compromised, leading to structural and functional deficits, including impaired cardiomyocyte shortening, reduced sarcoplasmic reticulum Ca^2+^ ATPase (SERCA) expression, increased expression of its inhibitor, phospholamban (PLB), and subsequent attenuation of the intracellular calcium transient [1], [2]. These changes establish a known substrate for contractile dysfunction and bradycardia. However, the mechanism linking thyroid deficiency to malignant electrical instability and stress-induced arrhythmias remains incompletely understood.

Cardiac arrhythmia is a leading cause of disability and sudden death worldwide, and patients with Hypo exhibit a heightened risk. However, since Hypo is associated with impaired metabolism, ventricular arrhythmias may be unmasked under stress conditions [3], [4]. Growing evidence suggests that unbalanced nitric oxide (NO) production and excessive oxidative stress play a key role in modulating ECC and electrical stability in various cardiac pathologies [5], [6]. NO is synthesized by nitric oxide synthases (NOS), with endothelial (eNOS), neuronal (nNOS), and inducible (iNOS) isoforms present in the heart [7].

Crucially, the concept that NOS uncoupling impairs NO-mediated signaling while generating cytotoxic reactive oxygen species (ROS) has been directly implicated in the genesis of severe arrhythmias [7]. Given the well-established ionic remodeling associated with hypothyroidism and the recognized role of ROS in promoting electrical instability (e.g., through calcium/calmodulin-dependent protein kinase II (CaMKII) activation), we hypothesized that hypothyroidism leads to the production of cytotoxic reactive free radicals via uncoupled NOS and that this mechanism is directly associated with stress-induced arrhythmia. Therefore, we aimed to thoroughly investigate the electrophysiological remodeling and assess whether NOS uncoupling contributes to heightened oxidative stress and the resulting arrhythmogenic susceptibility in a murine model of hypothyroidism. This study provides integrated mechanistic evidence demonstrating how impaired eNOS coupling converges with electrophysiological remodeling to promote ventricular arrhythmogenesis in hypothyroidism.

## 2 Methods

Additional methodological details are in the online supplement (Supplementary Methods).

### 2.1 Animals

The Swiss mice (25 - 30 g) were obtained from the Animal Care Facility of the Federal University of São Paulo (Ethics Committee for Animal Research number - UNIFESP #9389091220). All procedures involving mice were conducted in accordance with the guidelines of the National Council for the Control of Animal Experimentation (CONCEA), Brazil, and NIH Guide for the Care and Use of Laboratory Animals. Hypothyroidism was induced by adding 0.1% (w/vol) of methimazole to the their drinking water for 21 days [8], [9]. All the experimental procedures were performed on day 22. Mice were anesthetized via inhalation of isoflurane induced at 5% and maintained at 2% with 60% O_2_ [10]. For euthanasia, animals were deeply anesthetized with 5% isoflurane, followed by decapitation to allow subsequent heart collection.

### 2.2 *In vivo* electrocardiography recordings

Mice were anesthetized with isoflurane 2 %. Electrocardiographic (ECG) recordings were performed using a non-invasive system (ECG-PC version 2.07, Brazilian Electronic Technology—TEB, Belo Horizonte, MG, Brazil). Arrhythmia susceptibility in vivo was evaluated after the injection of caffeine (120 mg/kg, i.p.) and dobutamine (1 mg/kg,i.p.) [8]. To investigate the role of ROS on arrhythmia susceptibility, the animals were treated with N-Acetyl-l-cysteine (NAC-100 mg/kg, i.p.) or tetrahydrobiopterin (BH4, 20 mg/kg, i.p.- Sigma-Aldrich, St. Louis, MO, USA) [11], [12]. ECG analysis was performed using LabChart version 8 [13].

### 2.3 Cardiomyocyte isolation

Heparin (1000 UI/Kg, i.p.) was administered, and after 15 min mice were euthanized and their hearts removed. The aorta was then cannulated and promptly set up in a Langendorff apparatus for cardiomyocyte isolation. Experiments were performed using an enzymatic assay as described by Shioya (2007) [14].

### 2.4 Patch-clamp recordings

Patch-clamp recordings in both voltage-clamp and current-clamp modes were performed on isolated ventricular endocardial cardiomyocytes using an EPC-10 patch-clamp amplifier (HEKA, Holliston, Massachusetts, USA) and an AxoPatch 200B (Axon Instruments, Molecular Devices, USA) at room temperature (20–26°C). Data analysis was conducted using Clampfit 11.1 software (Molecular Devices, Sunnyvale, CA, USA).

### 2.5 Action potential recordings

Action potentials (APs) were recorded in the whole-cell current-clamp configuration. APs were evoked by a 1 nA square pulse of 3-5 ms duration at a frequency of 5 Hz. The internal pipette solution contained (in mM): 130 l-aspartate, 130 KOH, 20 KCl, 10 HEPES, 2 MgCl_2_.6H_2_O, and 5 NaCl. The pH was adjusted to 7.2 with KOH. The external solution was Tyrode solution (in mM): 140 NaCl, 5.4 KCl, 0.5 MgCl_2_.6H_2_O, 0.33 NaH_2_PO_4_, 11 glucose, 5 HEPES, and 1.8 CaCl_2_.2H_2_O (pH 7.4). This protocol was repeated in the absence and presence of KN-93, a CaMKII inhibitor [13]. To induce arrhythmia *in vitro*, cardiomyocytes were subject to a stimulus of 5 Hz (100 pulses) and then a pause of 10 s to observe spontaneous events [15].

### 2.6 Macroscopic ionic current recordings

All currents were recorded in the whole-cell voltage-clamp configuration.

#### 2.6.1 Total potassium currents (I_K_)

Voltage-dependent potassium currents, specifically the inwardly rectifying potassium current (I_K1_), transient outward (I_to_), delayed rectifier (I_Kdr_), and steady-state (I_ss_) currents, were determined. The internal pipette solution contained (in mM): 140 KCl, 1 MgCl_2_.6H_2_O, 11 EGTA, and 10 HEPES, pH adjusted to 7.2 with KOH. The external solution contained (in mM): 140 NMDG, 5.6 KCl, 1.8 CaCl_2_.2H_2_O, 0.1 CdCl_2_.H_2_O, 0.5 MgCl_2_.6H_2_O, 11 glucose, and 10 HEPES, pH adjusted to 7.4 with HCl [16], [17].

#### 2.6.2 Sodium current (I_Na_)

Macroscopic sodium currents were recorded with the following internal solution (in mM): 125 CsCl, 35 aspartic acid, 30 TEA-Cl, 5 Mg-ATP, 10 EGTA, 11 HEPES, 0.5 MgCl_2_ and 3.6 phosphocreatine (dissodium salt – 7.2 mM Na^+^), with pH adjusted to 7.3 with CsOH. The external solution contained (in mM): 125 TEA-Cl, 50 NaCl, 1.8 CaCl_2_, 5 CsCl, 1.2 MgCl_2_, 2 MnCl_2_, 20 HEPES, 11 glucose and 4-aminopyridine with pH adjusted to 7.3 with CsOH. To isolate I_Na_, 1 μM isradipine and 10 μM nifedipine were added to the external solution [18].

#### 2.6.3 Calcium current (I_Ca_)

For I_Ca_, the internal solution was (in mM): 120 CsCl, 20 TEA-Cl, 3 Na-ATP, 10 HEPES, 10 EGTA, and 2 Mg-ATP (pH 7.2 with CsOH). The external solution used was (in mM): 150 TEA-Cl, 0.5 MgCl_2_⋅6H_2_O, 1.8 CaCl_2_2.H_2_O, 10 HEPES, and 11 glucose (pH 7.4 with TEA-OH) [19].

#### 2.6.4 Sodium-calcium exchanger current (I_NCX_)

The I_NCX_ was recorded using a ramp protocol and measured as Ni^2+^ sensitive current. To isolate the I_NCX_, the internal pipette solution contained (in mM): 20 NaCl, 1.19 MgCl_2_, 10 HEPES, 20 TEA-Cl, 33.8 CaCl_2_, 40 EGTA, 40 aspartic acid, with pH adjusted to 7.2 with TEA-OH. The external solution was composed of (in mM): 145 NaOH, 145 aspartic acid, 5 HEPES, 2 MgCl_2_, 1 CaCl_2_, 2 BaCl_2_, with pH adjusted to 7.3 with NaOH. Ouabain (50 μM) was added to the bath solution to block the Na^+^/K^+^ pump, and nicardipine (2 μM) was used to block the L-type calcium current (I_Ca,L_) [20].

### 2.7 Calcium transient and spontaneous Ca^2+^ event recordings in ventricular cardiomyocytes

Isolated ventricular cardiomyocytes were loaded with 5 μM Fluo-4 AM in Tyrode's solution for 30 min at room temperature. After incubation, cells were rinsed and kept in fresh Tyrode's solution during imaging. Intracellular Ca^2+^ transients, Ca^2+^ sparks, and out-of-pacing calcium events were recorded under field stimulation at 5 Hz for 30 s to standardize excitation frequency. The amplitude of Ca^2+^ transients were analyzed using Clampfit 11 software (Molecular Devices, USA). Quantification of Ca^2+^ sparks was performed with ImageJ (NIH, USA) using the SparkMaster plug-in, which allows automated and reproducible detection of localized calcium release events in confocal line-scan images [8], [21], [22].

### 2.8 Measurements of ROS generation

ROS measurement in cardiomyocytes was evaluated using dihydroethidium (DHE), Mitotracker green and MitoSOX red. When appropriate, cardiomyocytes were pre-incubated with BH4, an eNOS cofactor; hydrogen peroxide (H_2_O_2_), an inducer of oxidative stress; apocynin, an NADPH oxidase inhibitor; N(G)-Nitro-l-arginine methyl ester (l-NAME) general NOS inhibitor; N(5)-(1-Iminoetil)-L-ornitina (L-NIO), specific inhibitor for eNOS; Nω-Propyl-l-arginine hydrochloride (NPLH), specific inhibitor for nNOS; 1400W dihydrochloride, specific inhibitor for iNOS [23], [24], [25]. For all analyses, we used Image J 1.52 × software (NIH, Bethesda, MD, USA). In all imaging experiments, at least one animal from each group was used on the same day to avoid artifacts from the imaging acquisition system.

### 2.9 Nitric oxide and nitrite levels

Nitric oxide (NO) production was assessed using two complementary approaches. Intracellular NO levels were evaluated in freshly isolated cardiomyocytes using 4-amino-5-methylamino-2′,7′-difluorofluorescein diacetate (DAF-FM diacetate; Invitrogen). Cells were incubated with DAF-FM diacetate for 30 min at room temperature in the dark, washed with Tyrode's solution to remove excess probe. Fluorescence intensity was quantified using ImageJ software. In parallel, NO production was indirectly determined by measuring nitrite levels using the Griess reaction. To further assess nitro-oxidative stress, myocardial 3-nitrotyrosine levels were quantified using a commercially available sandwich ELISA kit (Abcam, ab116691) according to the manufacturer's instructions.

### 2.10 Western blot analysis

Full-length gels and corresponding the Ponceau-stained membranes are provided in the supplementary material. To avoid contamination of endothelial proteins in Western blot analyses, protein expression was assessed in isolated left ventricular endocardial cardiomyocytes [26], [27]. Samples were extracted in RIPA buffer, and proteins were transferred to nitrocellulose membranes for immunoblotting. The membranes were incubated overnight and in a refrigerated environment, with the primary antibodies: anti-CaMKII delta (1:1000; A9196), anti-Oxi-CaMKII (1:1000, #071387), anti-phospho-CaMKII 1:500, #4301561), anti-eNOS (1:1000; sc-136977), anti-p^Ser1177^-eNOS (1:1000; sc- 12972), anti-nNOS (1:1000; sc-8309), anti-p^Ser852^-nNOS (1:1000; sc-19826), and anti-GAPDH (1:3000; sc-32233), anti-NOX2/gp91phox (1:2000; SAB5702937), anti-p22phox (1:1000; #27297), anti-p47phox (1:1000; #63290), anti-NOX4 (1:2000: MA5-32090), anti-Akt (1:500 A11016), anti-p^Ser473^-Akt (1:1000; #9271), anti-alpha-Tubulin (1:1000; #2144CS). After washing, the membranes were developed using enhanced chemiluminescence for immunodetection (Luminata strong™ - Western HRP substrate, Merck-Millipore). eNOS and nNOS coupling was determined as previously described [26].

### 2.11 Real-time quantitative RT-PCR (RT-qPCR)

Gene expression analysis (or target detection) was performed using two-step RT-qPCR with SYBR Green Master Mix (ThermoFisher, USA) [28]. Primers are detailed in the Supplementary Methods, and expression levels were normalized to the housekeeping gene GAPDH.

### 2.12 Statistics

GraphPad Prism software version 8.0.1 (Boston - USA) was used to conduct statistical and graphical representation. Data are expressed as mean ± SD. Normality was assessedusing the Shapiro-Wilk test. For comparisons between two groups, either unpaired Student's t-test or Mann–Whitney was applied, depending on data distribution. One-way or Two-way ANOVA followed by Bonferroni's post hoc test was used for multiple group comparisons. Fisher's exact test was applied for categorical data.

## 3 Results

### 3.1 Systemic electrophysiological alterations and potassium currents

The experimental design is summarized in Fig. 1A. After 21 days of methimazole treatment, mice developed a clear hypothyroid phenotype (Hypo), as confirmed by significant alterations in circulating T3, T4, and TSH levels (Supplementary Table 1).

**Fig. 1:**
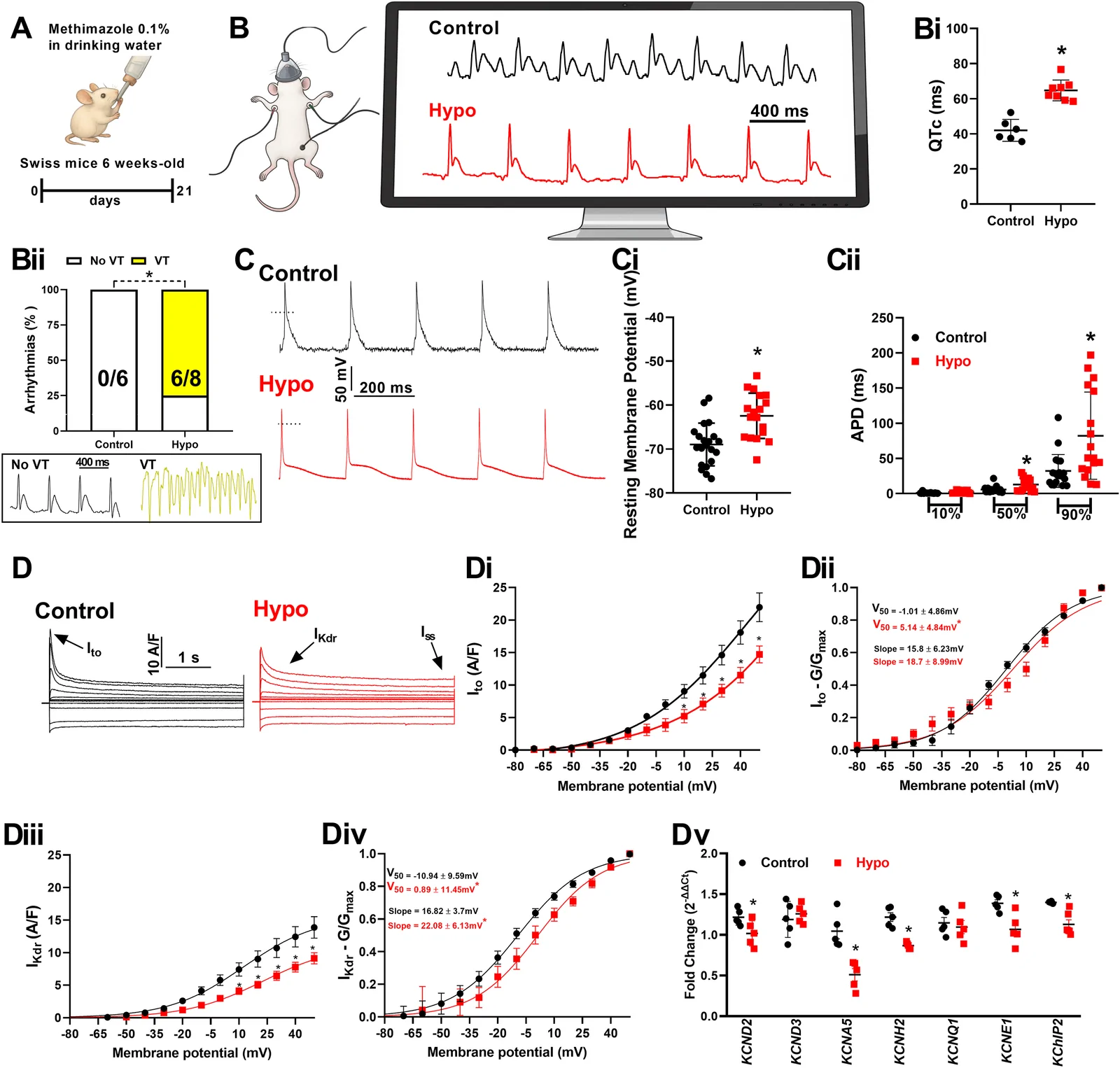
**Hypothyroidism induces electrophysiological remodeling associated with impaired ventricular repolarization and potassium channel downregulation.** (A) Experimental timeline showing induction of hypothyroidism by methimazole administration in drinking water for 21 days. (B) Representative in vivo electrocardiographic recordings obtained from Control and hypothyroid (Hypo) mice. (Bi) Quantification of corrected QT (QTc) interval. (Bii) Incidence of ventricular arrhythmias following adrenergic stress induced by dobutamine–caffeine challenge. (C) Representative ventricular action potential (AP) recordings from isolated left ventricular cardiomyocytes. (Ci) Resting membrane potential. (Cii) Action potential duration at 10% (APD_10_) , 50% (APD_50_), and 90% (APD_90_) of repolarization. (D) Representative potassium current recordings. (Di) Current density–voltage (I–V) relationship for the transient outward potassium current (I_to_). (Dii) Steady-state activation curves for I_to_. (Diii) Current density–voltage relationship for the delayed rectifier potassium current (I_Kdr_). (Div) Steady-state activation curves for I_Kdr_. (Dv) Relative mRNA expression of potassium channel subunits (*Kcnd2, Kcnd3, Kcna5, Kcnh2, Kcnq1, Kcne1, and Kchip2*) in left ventricular tissue. Data are presented as mean ± SD. Statistical analyses were performed using unpaired Student's t-test (Bi, Ci, Cii, and Dv), two-way ANOVA followed by Bonferroni's multiple comparisons test (Di–Div), or Fisher's exact test (Bii), as appropriate. N = 6–8 animals per group for in vivo analyses (B), N = 5 animals per group for electrophysiological experiments (C and D), and n = 18–21 isolated cardiomyocytes.

*In vivo* ECG recordings obtained from anesthetized mice revealed marked electrophysiological disturbances in the Hypo group compared with the Control group. These included a significant reduction in heart rate and prolongation of the PR interval (Supplementary Table 2), consistent with previous reports [29], [30] (Fig. 1B). Notably, the corrected QT (QTc) interval was significantly prolonged in Hypo animals (Fig. 1Bi).

During adrenergic stress induced by the dobutamine–caffeine challenge, Hypo mice exhibited a blunted chronotropic response, with approximately a 30% reduction in heart rate increase compared with the Control group (Supplementary Fig. 1A). Despite this attenuated response, the stress challenge triggered ventricular arrhythmias in the majority of Hypo animals, with ventricular tachycardia observed in 6 out of 8 mice (Fig. 1Bii), confirming enhanced arrhythmogenic susceptibility [2].

To identify the cellular mechanisms underlying these abnormalities, action potentials (APs) were recorded from isolated left ventricular cardiomyocytes paced at 5 Hz (Fig. 1C). Hypo cardiomyocytes displayed a significant depolarization of the resting membrane potential (∼10 mV) compared with the Control group (Fig. 1Ci). Moreover, AP duration (APD) was markedly prolonged at both 50% and 90% of repolarization (APD_50_ and APD_90_; Fig. 1Cii; Supplementary Table 3).

This AP prolongation was associated with significant reductions in the repolarizing currents I_to_ (transient outward) and I_Kdr_ (delayed rectifier) (Fig. 1D, Di and Diii), accompanied by a rightward shift in their steady-state activation curves (Fig. 1 Dii and Div). In contrast, the sustained outward potassium current (I_SS_) and its activation kinetics were not altered in the Hypo group (Supplementary Fig. 1C and D). Additionally, inward rectifier potassium current (I_K1_), primarily mediated by Kir2.1 (KCNJ2) channels, was reduced at membrane potentials more negative than −90 mV (Supplementary Fig. 1E and F), potentially contributing to resting membrane depolarization. No differences were observed in steady-state inactivation of either I_to_ or I_Kdr_ (Supplementary Fig. 2A and B). However, recovery from inactivation revealed a selective increase in the slow recovery time constant (τ_2_) of I_to_ in Hypo cardiomyocytes, (Supplementary Fig. 2C), while I_Kdr_ recovery from inactivation remained unchanged (Supplementary Fig. 2D).

Because membrane capacitance was significantly lower in Hypo cardiomyocytes, all ionic currents were normalized to capacitance to account for differences in cell size and allow direct comparison of current densities (Supplementary Fig. 1B).

Consistent with these functional alterations, quantitative gene expression analysis revealed significant downregulation of mRNA for key potassium channel subunits, including *Kcnd2* (Kv4.2; I_to_), *Kcna5* (Kv1.5; I_Kur_), *Kcnh2* (Kv11.1; I_Kr_), as well as accessory subunits *Kcne1* and *Kchip2* (Fig. 1Dv).

### 3.2 Sodium and calcium currents are increased in hypothyroidism

Representative current–voltage (I–V) relationships of peak sodium current (I_Na_) are shown in Fig. 2A. Hypothyroidism induced significant remodeling of peak I_Na_ density. At −20 mV, current density increased from −114.7 ± 41.08 pA/pF in Control cardiomyocytes to −160.0 ± 56.08 pA/pF in the Hypo group (Fig. 2Ai). Despite this increase in current density, no significant differences were observed in the steady-state activation (G–V), half-activation potential (V_50_), or slope factor between groups (Fig. 2Aii).

**Fig. 2:**
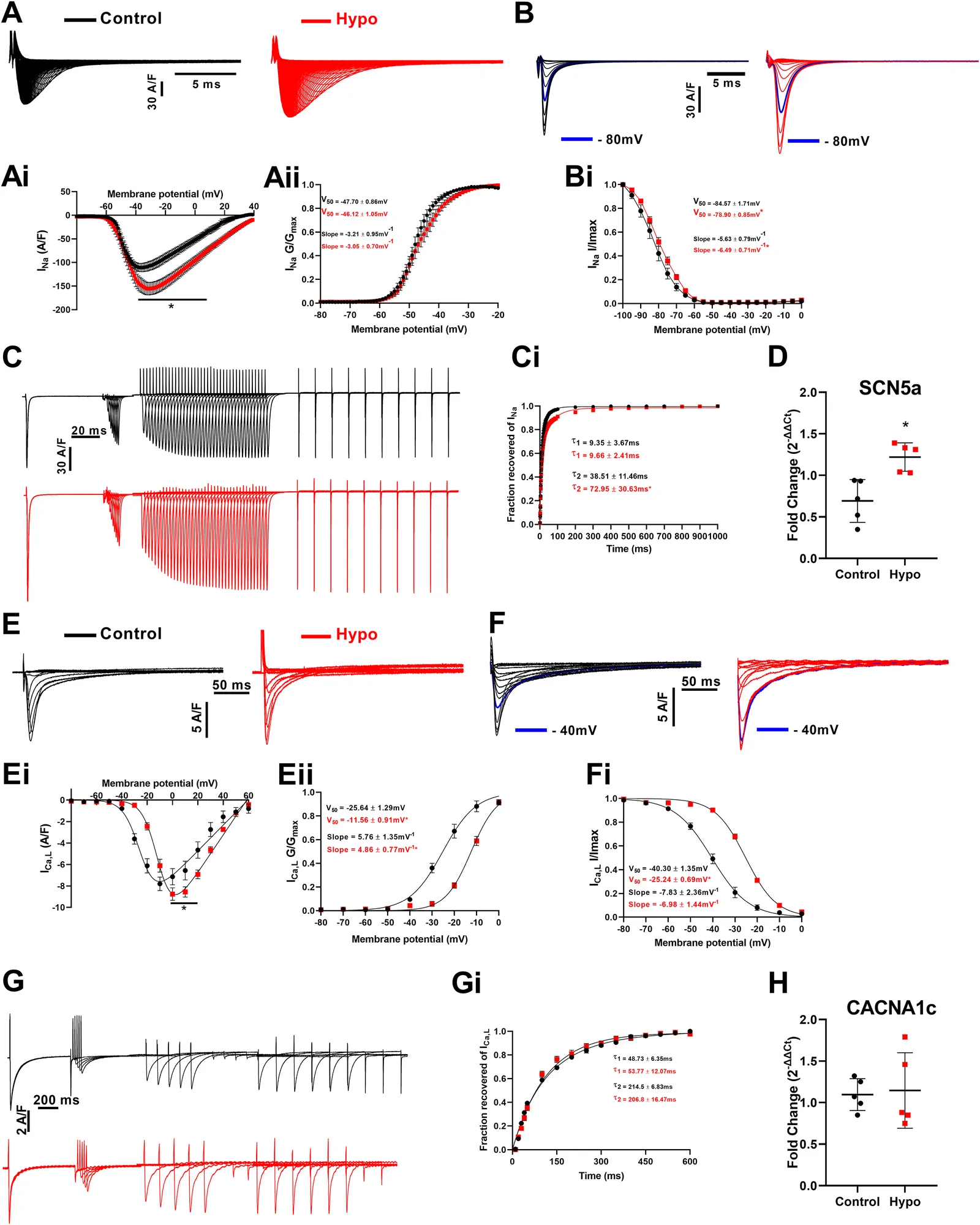
**Hypothyroidism enhances sodium and calcium currents in ventricular cardiomyocytes.** (A) Representative whole-cell sodium current (I_Na_) recordings obtained from Control and hypothyroid (Hypo) cardiomyocytes. (Ai) Current density–voltage (I–V) relationship for peak I_Na_. (Aii) Steady-state activation curves. (B) Representative steady-state inactivation protocol. (Bi) Steady-state inactivation curves. (C) Representative recovery-from-inactivation protocol. (Ci) Recovery kinetics fitted with a biexponential function. (D) Relative *Scn5a* mRNA expression in isolated left ventricular cardiomyocytes. (E) Representative L-type calcium current (I_Ca,L_) recordings. (Ei) Current density–voltage relationship for I_Ca,L_. (Eii) Steady-state activation curves. (F) Representative steady-state inactivation protocol for I_Ca,L_. (Fi) Steady-state inactivation curves. (G) Representative recovery-from-inactivation protocol. (Gi) Recovery kinetics. (H) Relative *Cacna1c* mRNA expression. Data are presented as mean ± SD. Statistical analyses were performed using two-way ANOVA followed by Bonferroni's multiple comparisons test (Ai and Ei) or unpaired Student's t-test (D and H), as appropriate. N = 5 animals per group; n = 15–20 isolated cardiomyocytes.

Analysis of sodium channel steady-state inactivation revealed important gating alterations. Representative traces from the steady-state inactivation protocol (Fig. 2B), demonstrated a positive shift in the half-inactivation potential in Hypo cardiomyocytes (Fig. 2Bi). This shift was accompanied by a significant change in the slope factor, indicating an increased fraction of available sodium channels over a broader voltage range. Moreover, recovery from inactivation was significantly delayed in Hypo cardiomyocytes, as reflected by an increase in the slow recovery time constant (τ_2_), without significant changes in the fast component fraction (τ_1_) (Fig. 2C and Ci).

Consistent with these functional changes, quantitative gene expression analysis of isolated left ventricular cardiomyocytes revealed a significant upregulation of *Scn5a* (Fig. 2D), which encodes the α-subunit of the predominant cardiac sodium channel, Na_v_1.5.

We next evaluated the calcium current (I_Ca_) (Fig. 2E). Hypo cardiomyocytes also exhibited increased peak I_Ca_ density, rising from −7.53 ± 2.52 pA/pF in Control to −9.56 ± 2.14 pA/pF at 0 mV in the Hypo group (Fig. 2Ei). This increase was accompanied by a rightward shift in the steady-state activation toward more positive potentials in Hypo cardiomyocytes, along with a reduction in the slope factor (Fig. 2Eii).

In steady-state inactivation experiments, the half-inactivation potential was significantly more positive in Hypo cardiomyocytes (−25 mV) compared with Control (−40 mV) (Fig. 2F and Fi). In contrast, no differences were observed in recovery from inactivation kinetics between groups (Fig. 2G and Gi). Notably, these functional alterations occurred without detectable changes in *Cacna1c* gene expression, which encodes the α1C subunit of the L-type calcium channel (Fig. 2H), the major contributor to I_Ca_ in adult murine ventricular cardiomyocytes.

### 3.3 Redox-dependent CaMKII activation contributes to electrical instability in hypothyroid cardiomyocytes

To further investigate mechanisms contributing to AP instability and arrhythmogenesis in hypothyroidism, we next evaluated Na^+^/Ca^2+^ exchanger (NCX) activity, cellular excitability, and CaMKII signaling.

The Na^+^/Ca^2+^ exchanger current (I_NCX_) was assessed as the Ni^2+^-sensitive current. Hypo cardiomyocytes exhibited a significant reduction in Ni^2+^-sensitive current density at +40 and + 50 mV (Supplementary Fig. 3A), indicating impaired NCX function under depolarized conditions. Representative current traces recorded before and after Ni^2+^ application are shown individually (Supplementary Fig. 3A and Ai) and as Ni^2+^ sensitive currents (Supplementary Fig. 3Aii). These functional alterations occurred in the absence of changes in *Slc8a1* (NCX1) gene expression (Supplementary Fig. 3B), suggesting post-transcriptional/regulatory modulation of NCX function or changes in free intracellular [Ca^2+^] or [Na^+^].

To determine whether these electrophysiological alterations translated into increased electrical instability, AP duration heterogeneity across the ventricular myocardium was studied. AP duration dispersion, defined as the variability of repolarization timing among cardiomyocytes, was significantly increased in Hypo cardiomyocytes at 90% repolarization (APD_90_ dispersion; Fig. 3Ai). In parallel, Hypo cardiomyocytes displayed enhanced electrical instability, characterized by spontaneous depolarizations following a 10 s pause after fast pacing (Fig. 3Aii), resulting in a significantly elevated proarrhythmic score (Fig. 3Aiii). Consistent with this heightened excitability, intracellular Ca^2+^ handling was markedly disrupted. Hypo cardiomyocytes exhibited reduced Ca^2+^ transient amplitude and an increased frequency of spontaneous Ca^2+^ sparks occurring between stimulation pulses, indicative of asynchronous (out-of-pacing) calcium release events (Supplementary Fig. 3C–F), in agreement with previous reports [2], [8].

**Fig. 3:**
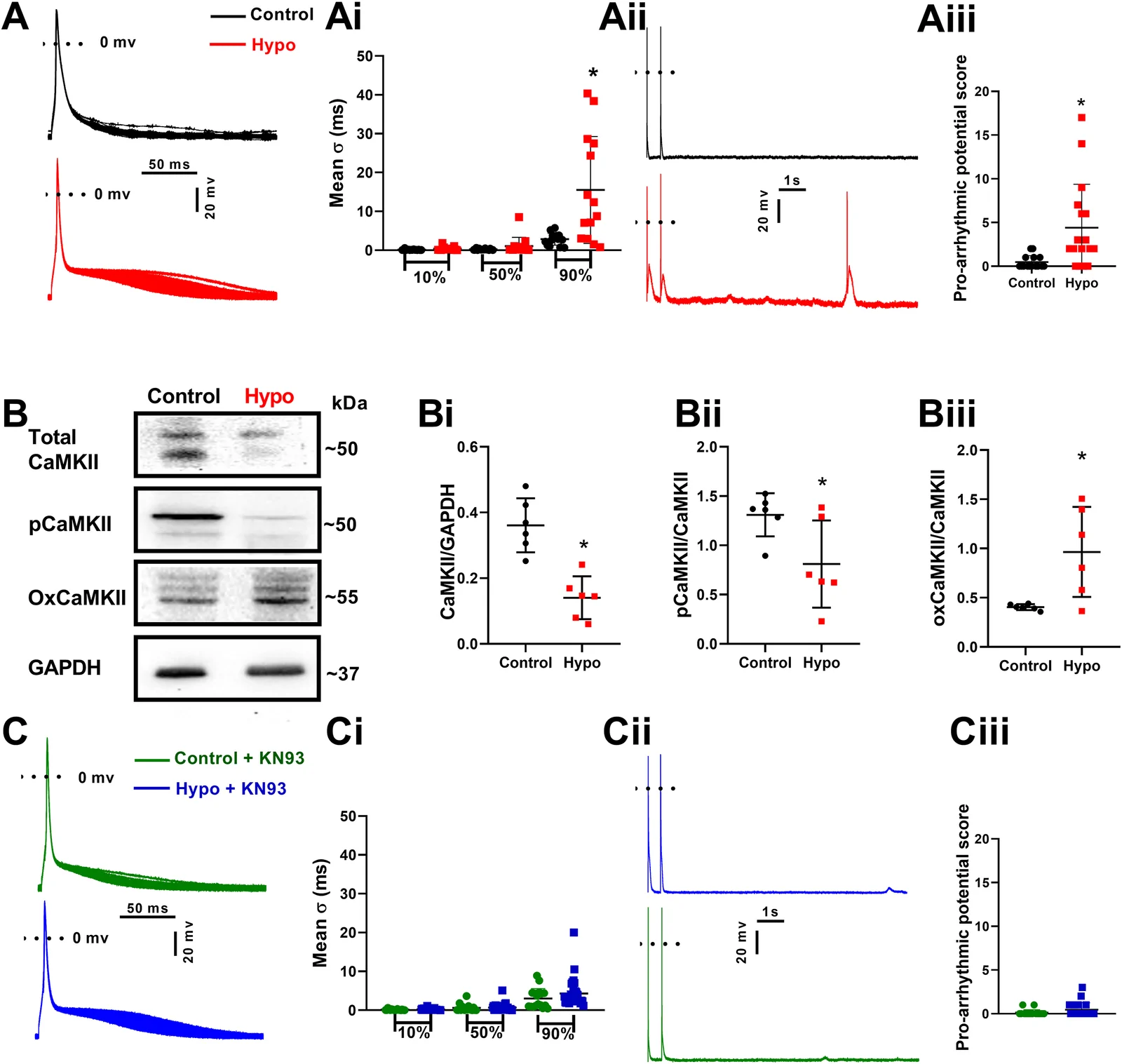
**Redox-dependent CaMKII activation contributes to electrical instability in hypothyroid cardiomyocytes.** (A) Increased action potential (AP) dispersion and spontaneous electrical activity in hypothyroid (Hypo) cardiomyocytes. (Ai) Quantification of AP duration dispersion at 90% repolarization (APD_90_). (Aii) Representative recordings showing spontaneous depolarizations following a 10-s pause after pacing. (Aiii) Quantification of the proarrhythmic score based on delayed afterdepolarizations (DADs) and triggered activity. (B) Representative immunoblots of total CaMKII, phosphorylated CaMKII (p-CaMKII), oxidized CaMKII (oxCaMKII), and GAPDH. (Bi–Biii) Densitometric analysis of total CaMKII, p-CaMKII, and oxCaMKII normalized to total CaMKII, respectively. (C) Effects of CaMKII inhibition with KN-93 on cardiomyocyte electrical activity. (Ci) Representative AP recordings. (Cii) APD_90_ dispersion following KN-93 treatment. (Ciii) Quantification of spontaneous depolarizations after pacing. Data are presented as mean ± SD. Statistical significance was determined using unpaired Student's t-test. N = 5–6 animals per group, with n = 15–20 isolated cardiomyocytes.

Because CaMKII is a central regulator of calcium handling and electrical stability in cardiomyocytes, we next assessed CaMKII expression and post-translational modification. Total CaMKII protein levels were significantly reduced in Hypo left ventricular endocardial cardiomyocytes when normalized to GAPDH (Fig. 3B–Bi). Phosphorylated CaMKII (pCaMKII) levels were also reduced (Fig. 3B and Bii; Supplementary Fig. 3G). In contrast, oxidized CaMKII (oxCaMKII) levels normalized to GAPDH were unchanged (Supplementary Fig. 3H). Normalization of oxCaMKII to total CaMKII revealed a significant increase in Hypo cardiomyocytes (Fig. 3Biii), indicating a shift toward redox-dependent CaMKII activation.

To evaluate the functional relevance of CaMKII activity to the observed disrupted cardiomyocyte excitability, cardiomyocytes were treated with the CaMKII inhibitor KN-93. KN-93 markedly reduced AP dispersion and prevented spontaneous depolarizations following the 10 s pause (Fig. 3C–Ciii), restoring cellular excitability toward control levels. In addition, KN-93 significantly reduced the proportion of cells exhibiting arrhythmic events, including triggered activity (Supplementary Fig. 3I–K).

KN-93 treatment also normalized resting membrane potential values (from −61.22 ± 4.96 to −70.42 ± 5.52 mV). While AP duration at 50% repolarization (APD_50_) remained prolonged compared with Controls, APD_90_ was significantly shortened relative to untreated Hypo cardiomyocytes, although it did not fully return to Control levels (Supplementary Table 3).

### 3.4 Nitric oxide synthase-dependent oxidative stress underlies impaired NO bioavailability in hypothyroid cardiomyocytes

Oxidative stress in isolated left ventricular cardiomyocytes was assessed using complementary fluorescence-based approaches (Fig. 4A). Representative pseudo-colored images obtained with dihydroethidium (DHE) revealed a marked increase in ROS-sensitive fluorescence in Hypo cardiomyocytes compared with Controls (Fig. 4B). Quantitative analysis demonstrated a 72.8% increase in DHE fluorescence intensity in the Hypo group, indicating enhanced cytosolic oxidative stress.

**Fig. 4:**
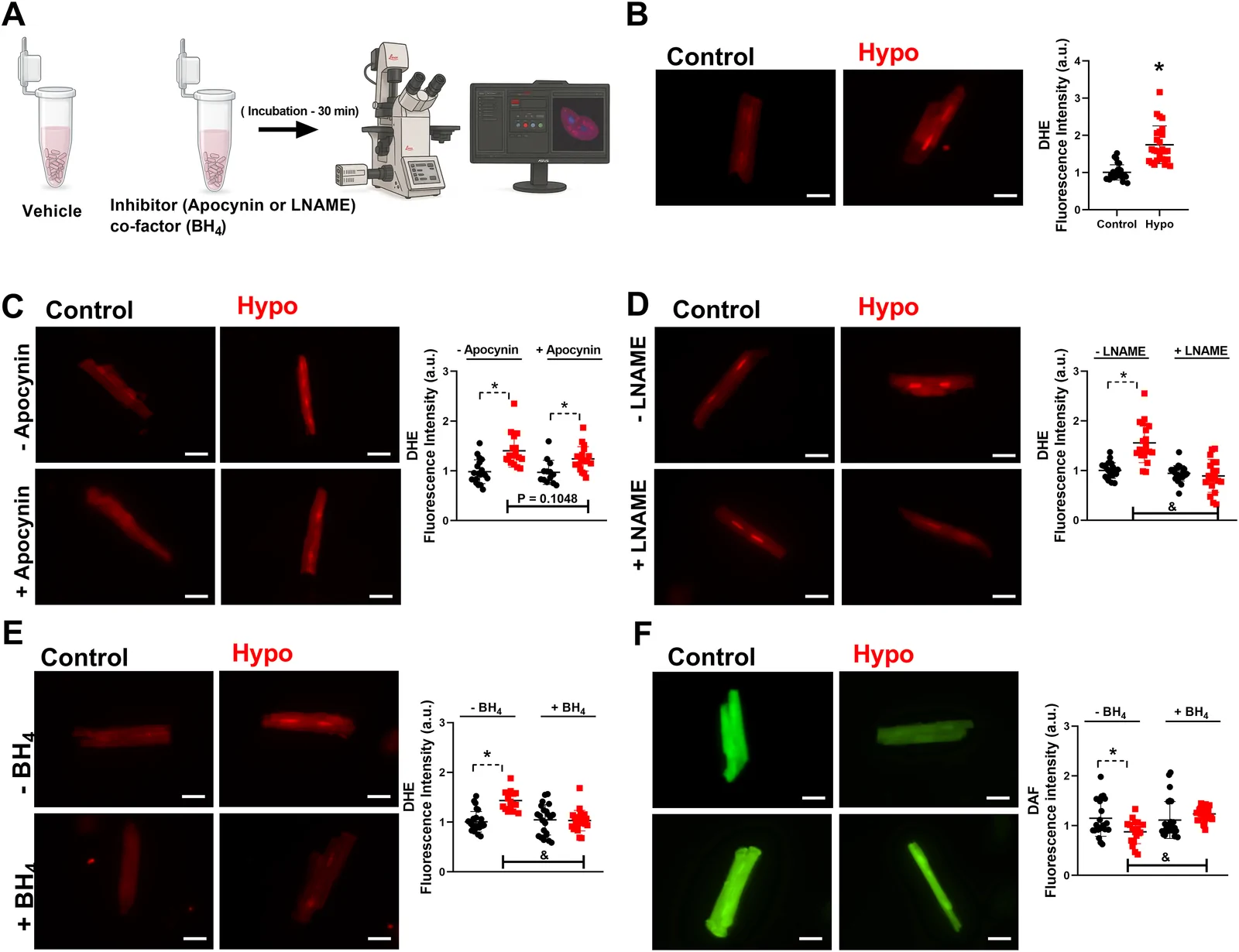
**Impaired nitric oxide bioavailability and NOS-dependent oxidative stress in hypothyroid cardiomyocytes.** (A) Experimental workflow illustrating ROS and nitric oxide (NO) measurements in isolated ventricular cardiomyocytes. (B) Representative pseudo-colored dihydroethidium (DHE) fluorescence images and quantification of cytosolic ROS levels. (C) Effect of NADPH oxidase inhibition with apocynin on DHE fluorescence. (D) Effect of the non-selective nitric oxide synthase inhibitor l-NAME on ROS production. (E) Effect of tetrahydrobiopterin (BH4) supplementation on DHE fluorescence. (F) Representative DAF-FM fluorescence images and quantification of intracellular NO production in Control and hypothyroid (Hypo) cardiomyocytes in the absence or presence of BH4. Scale bar = 20 μm. Data are presented as mean ± SD. Statistical significance was assessed using unpaired Student's t-test. N = 5 animals per group, with n = 30 cardiomyocytes analyzed per group.

To evaluate the contribution of mitochondrial ROS, cardiomyocytes were colabeled with MitoTracker Green to estimate mitochondrial content and MitoSOX Red to assess mitochondrial superoxide-associated fluorescence. Hypo cardiomyocytes exhibited a significant reduction in mitochondrial signal (31 ± 0.23%) accompanied by a 36 ± 0.14% decrease in MitoSOX fluorescence (Supplementary Fig. 4A–C), suggesting that mitochondria are unlikely to represent the primary source of increased cytosolic ROS in this model.

To investigate the potential involvement of NADPH oxidases, cardiomyocytes were treated with the NOX inhibitor apocynin. Apocynin did not reduce DHE fluorescence in Hypo cardiomyocytes (Fig. 4C). Consistently, protein expression levels of NOX4, gp91^phox^, and p22^phox^ were not altered between Control and Hypo groups (Supplementary Fig. 4D–G). Although p47^phox^ protein levels were reduced in Hypo cardiomyocytes (Supplementary Fig. 4H), this change did not correspond to decreased ROS production, suggesting that NADPH oxidases are unlikely to account for the increased ROS observed in Hypo cardiomyocytes.

In contrast, inhibition of nitric oxide synthase (NOS) with l-NAME normalized ROS-sensitive fluorescence in Hypo cardiomyocytes to levels comparable with Controls (Fig. 4D). Similarly, supplementation with tetrahydrobiopterin (BH4) 30 min prior fluorescence level measurements, a critical NOS cofactor, restored ROS levels in Hypo cardiomyocytes to those observed in Control cells (Fig. 4E).

To further assess NO bioavailability, NO production was measured using the NO-sensitive probe difluorofluorescein diacetate (DAF-FM). Hypothyroid cardiomyocytes exhibited significantly reduced NO-dependent fluorescence compared with Controls (Fig. 4F). Consistent with these findings, nitrite levels measured by the Griess reaction were also significantly reduced in Hypo cardiomyocytes (Supplementary Fig. S4I), and 30 min prior BH4 treatment restored nitrite levels to values comparable to those observed in the Control group, further supporting impaired NO bioavailability. Moreover, myocardial 3-nitrotyrosine levels were significantly increased, consistent with increased nitro-oxidative stress and suggesting increased formation of reactive nitrogen species, most likely peroxynitrite, secondary to the interaction between superoxide and nitric oxide (Supplementary Fig. S4J). Importantly, BH4 treatment markedly reduced 3-nitrotyrosine levels, indicating attenuation of nitro-oxidative stress possibly through restoration of eNOS coupling. Together, these findings support the concept that eNOS uncoupling shifts nitric oxide synthase activity from NO production toward superoxide generation, thereby reducing NO bioavailability while promoting protein nitration. Notably, BH4 treatment restored NO production to Control levels (Fig. 4F), consistent with improved eNOS coupling.

### 3.5 Impaired Akt/eNOS signaling contributes to oxidative stress in hypothyroid cardiomyocytes

To further identify the enzymatic source of increased ROS in Hypo cardiomyocytes, selective nitric oxide synthase (NOS) inhibitors were employed (Fig. 5A). Treatment with L-NIO, a selective endothelial NOS (eNOS) inhibitor, significantly reduced DHE-sensitive fluorescence in Hypo cardiomyocytes (Fig. 5Ai), indicating that eNOS activity contributes to ROS generation under hypothyroid conditions. In contrast, when Hypo cells were incubated with inhibitors of nNOS or iNOS (Fig. 5Ai), DHE-sensitive fluorescence was not restored to the Control levels.

**Fig. 5:**
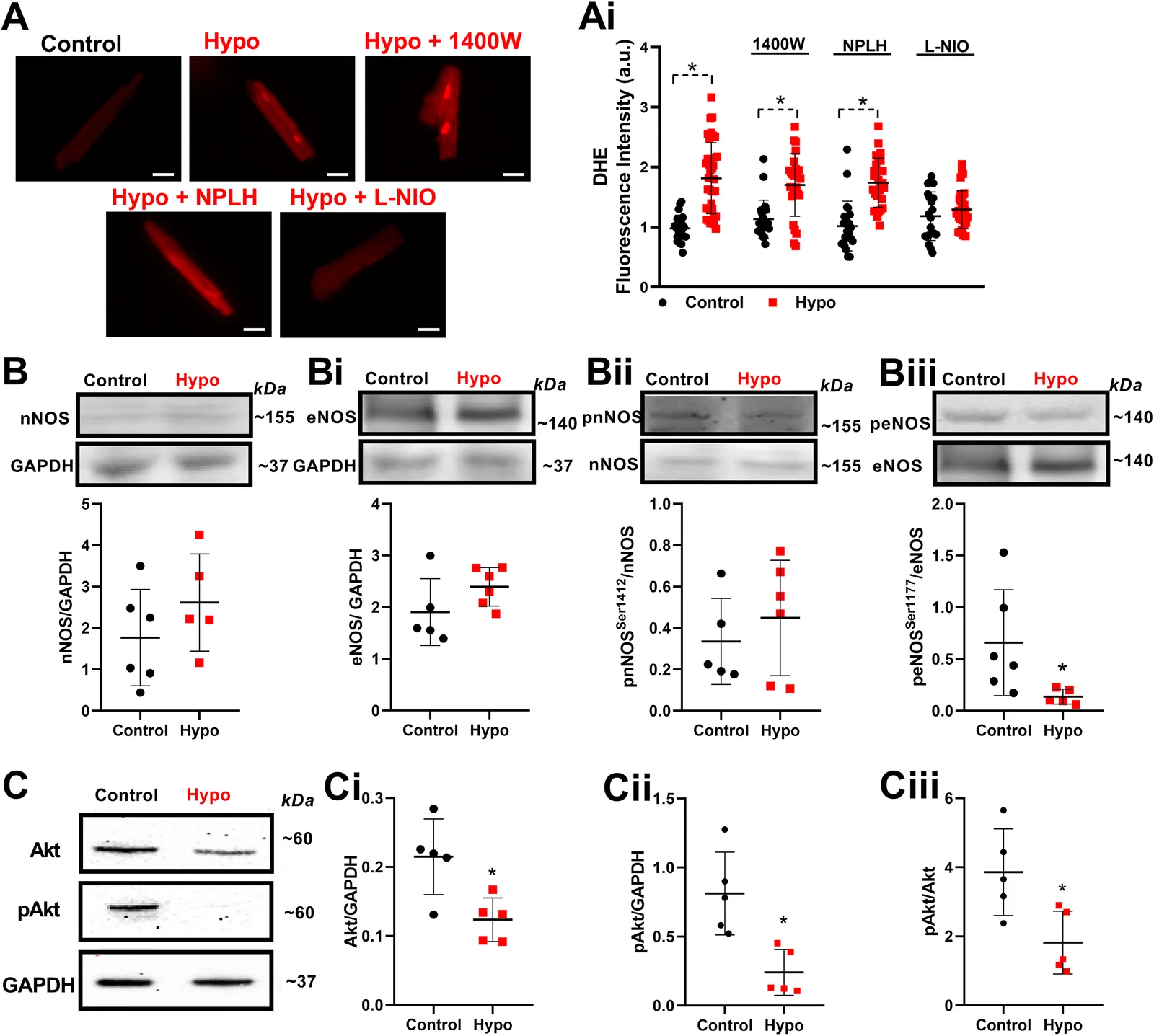
**Impaired Akt-dependent eNOS signaling contributes to oxidative stress in hypothyroid cardiomyocytes.** (A) Representative DHE fluorescence images illustrating the effects of selective inhibition of iNOS (1400W), nNOS (Nω-propyl-l-arginine hydrochloride; NPLH), and eNOS (L-NIO) on ROS production in isolated ventricular cardiomyocytes. (Ai) Quantification of DHE fluorescence intensity. (B) Representative immunoblots of total eNOS, phosphorylated eNOS (Ser1177), total nNOS, phosphorylated nNOS (Ser852), and GAPDH. (Bi–Biii) Densitometric analyses of total eNOS, total nNOS, and the phospho-eNOS/eNOS ratio. (C) Representative immunoblots of total Akt, phosphorylated Akt (Ser473), and GAPDH. (Ci–Ciii) Quantification of total Akt, phosphorylated Akt, and the phospho-Akt/Akt ratio. Scale bar = 20 μm. Data are presented as mean ± SD. Statistical analyses were performed using unpaired Student's t-test. N = 5–6 animals per group; n = 30 cardiomyocytes for fluorescence analyses.

Western blot analysis revealed no significant differences in total protein expression or phosphorylation levels of neuronal NOS (nNOS) or total eNOS between Control and Hypo groups (Fig. 5B–Bii). However, the phosphorylated-to-total eNOS ratio (peNOS/eNOS) was significantly reduced in Hypo cardiomyocytes (Fig. 5Biii), indicating altered regulation of eNOS activation.

To assess whether upstream signaling pathways might underlie this altered eNOS phosphorylation status, Akt signaling was examined. Both total Akt and phosphorylated Akt protein levels were significantly reduced in Hypo cardiomyocytes (Fig. 5C and Ci), resulting in a decreased phosphorylated-to-total Akt ratio (pAkt/Akt) (Fig. 5Cii and Ciii). These findings indicate an impairment of Akt-dependent signaling in hypothyroid cardiomyocytes, consistent with the reduced phosphorylation of eNOS.

### 3.6 eNOS uncoupling drives oxidative activation of CaMKII and arrhythmogenic susceptibility in hypothyroidism

To evaluate NOS coupling status, the dimer-to-monomer ratio of NOS isoforms was assessed under non-reducing conditions. No significant differences were detected in nNOS coupling between Control and Hypo cardiomyocytes (Fig. 6A and Ai, left panels). In contrast, the eNOS dimer-to-monomer ratio was significantly reduced in Hypo cardiomyocytes (Fig. 6A and Aii, right panels), consistent with eNOS uncoupling in hypothyroid cardiomyocytes.

**Fig. 6:**
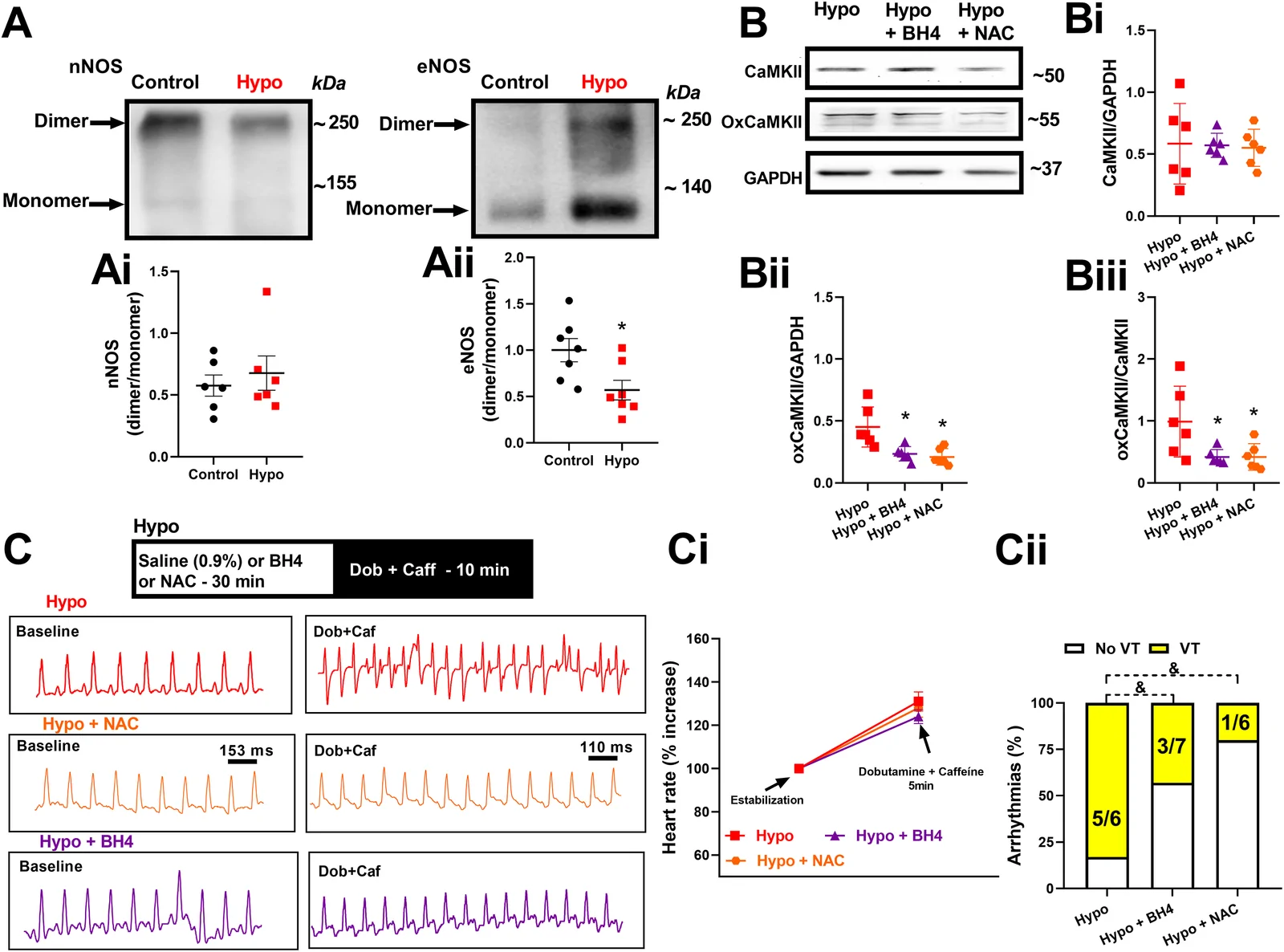
**eNOS uncoupling contributes to arrhythmogenic susceptibility in hypothyroid mice.** (A) Representative immunoblots obtained under non-reducing conditions showing nNOS and eNOS dimers and monomers. (Ai–Aii) Quantification of the dimer-to-monomer ratio for nNOS and eNOS. (B) Representative immunoblots of total CaMKII and oxidized CaMKII (oxCaMKII) in hypothyroid (Hypo) mice acutely (30 min before euthanasia) treated with tetrahydrobiopterin (BH_4_) or N-acetyl-l-cysteine (NAC). (Bi–Biii) Densitometric analysis of total CaMKII, oxCaMKII normalized to GAPDH, and the oxCaMKII/CaMKII ratio. (C) Experimental protocol for in vivo evaluation of arrhythmia susceptibility following BH_4_ or NAC pretreatment. (Ci) Representative ECG recordings obtained before and after dobutamine–caffeine challenge. (Cii) Heart rate response during adrenergic stimulation. (Ciii) Incidence of ventricular arrhythmias following pharmacological treatments. Data are presented as mean ± SD. Statistical analyses were performed using unpaired Student's t-test (A), one-way ANOVA followed by Bonferroni's multiple comparisons test (B), or Fisher's exact test (Ciii), as appropriate. N = 6 animals per group for biochemical analyses and N = 6–7 animals per group for in vivo experiments.

Because eNOS uncoupling is expected to enhance oxidative signaling, we next examined whether pharmacological restoration of eNOS coupling or antioxidant treatment modulates CaMKII oxidation. Total CaMKII protein expression was not altered by either BH4 or NAC treatment (Fig. 6B and Bi). In contrast, both interventions significantly reduced oxidized CaMKII (oxCaMKII) levels when normalized to GAPDH (Fig. 6Bii). Likewise, the oxCaMKII/CaMKII ratio was significantly decreased following BH4 or NAC treatment (Fig. 6Biii), indicating that both acute restoration of eNOS coupling and antioxidant therapy effectively attenuate oxidative activation of CaMKII without affecting total CaMKII abundance.

We then investigated whether attenuation of oxidative signaling translated into reduced arrhythmia susceptibility in vivo. Hypo mice were pretreated with tetrahydrobiopterin (BH4) or N-acetylcysteine (NAC) 30 min before ECG acquisition and the dobutamine–caffeine challenge (Fig. 6C). Neither treatment affected the stress-induced increase in heart rate (Fig. 6Ci). However, both BH4 and NAC reduced ventricular arrhythmia incidence from 83% in untreated Hypo mice to 43% and 16.6%, respectively (Fig. 6Cii). Despite this marked reduction in arrhythmias, neither intervention normalized PR or QTc intervals (Supplementary Table 2).

## 4 Discussion

The present study identifies a previously unrecognized mechanism underlying the increased arrhythmogenic vulnerability associated with hypothyroidism. Our findings support a hierarchical pathophysiological pathway in which thyroid hormone deficiency impairs eNOS signaling, leading to eNOS uncoupling, reduced nitric oxide (NO) bioavailability, oxidative activation of CaMKII, and progressive electrical remodeling that culminates in stress-induced ventricular arrhythmias.

In humans, Hypo is associated with several cardiac rhythm disturbances, most commonly sinus bradycardia, affecting more than 50% of patients, as well as QT interval prolongation [29]. Consistent with these clinical observations, our experimental model recapitulates key features of electrical dysfunction seen in hypothyroid patients [30]. Hypo mice exhibited reduced heart rate and marked QTc prolongation under basal conditions. Although the chronotropic response to adrenergic stimulation was blunted, stress challenge unmasked a pronounced proarrhythmic susceptibility, culminating in episodes of ventricular tachycardia. These in vivo abnormalities are consistent with intrinsic cardiomyocyte dysfunction, characterized by hyperexcitability and impaired repolarization, as previously reported in similar models [1], [8], [11].

AP prolongation is a well-established hallmark of hypothyroid cardiomyocytes and is primarily attributed to impaired ventricular repolarization. Consistent with previous reports, we observed significant reductions in I_to_, I_Kdr_, and I_K1_, accompanied by downregulation of several potassium channel subunits, including *Kcnd2, Kcna5, Kcnh2, Kcne1, and Kchip2*. These changes were associated with altered I_to_ gating, further reducing repolarization reserve. In parallel, enhanced peak I_Na_ and I_Ca,L_, together with altered channel kinetics, increased inward depolarizing currents, collectively creating an electrophysiological substrate that favors action potential prolongation and arrhythmogenesis [8], [31], [32], [33].

The reduction in I_NCX_ observed in hypothyroid cardiomyocytes indicates impaired Ca^2+^ extrusion capacity, suggesting defective intracellular calcium homeostasis. Although the direct functional consequences of this remodeling remain to be fully established, is expected to prolong intracellular calcium elevation during diastole and thereby increase susceptibility to spontaneous calcium release. Nevertheless, our data indicate that NCX remodeling alone is unlikely to represent the primary arrhythmogenic mechanism. Instead, because oxidized CaMKII was markedly increased, reduced exchanger activity most likely amplifies an already unstable calcium signaling environment probably created by oxidative CaMKII activation. Thus, NCX dysfunction should be viewed as an important contributor that exacerbates calcium dysregulation rather than the initiating mechanism of electrical instability. This interpretation is consistent with the broader concept that oxidative stress, rather than isolated ion transporter dysfunction, orchestrates the arrhythmogenic remodeling associated with hypothyroidism [34], [35], [36].

Disrupted intracellular Ca^2+^ homeostasis represents a critical convergence point in the development of electrical instability in hypothyroid cardiomyocytes. Previous studies, including our own, have consistently demonstrated reduced Ca^2+^ transient amplitude and impaired sarcoplasmic reticulum (SR) Ca^2+^ release, largely attributable to altered SERCA–phospholamban regulation [2], [8]. Under these conditions, reduced SR calcium loading would be expected to suppress spontaneous calcium release and consequently reduce arrhythmogenic activity. Surprisingly, however, hypothyroid cardiomyocytes exhibited increased Ca^2+^ spark frequency and spontaneous Ca^2+^ waves following stress, indicating that mechanisms other than SR calcium load govern calcium instability in this model. Our findings strongly suggest that this apparent paradox is explained by redox-dependent activation of CaMKII.

Although both total and phosphorylated CaMKII levels were reduced—likely reflecting diminished Ca^2+^/calmodulin-dependent activation due to smaller Ca^2+^ transients—we observed a relative increase in oxidized CaMKII (OxCaMKII). Under oxidative conditions, oxidation of regulatory methionine residues converts CaMKII into a constitutively active kinase that no longer depends on Ca^2+^/calmodulin for activation [37]. This redox-driven activation could sustain kinase activity despite reduced Ca^2+^ availability, possibly promoting RyR2 hyperphosphorylation, increased SR Ca^2+^ leak, and spontaneous Ca^2+^ release events. Thus, a possible consequence would be that oxidative CaMKII activation overrides the expected protective effect of reduced SR Ca^2+^ load, driving proarrhythmic Ca^2+^ instability [37], [38].

An additional aspect deserving consideration is the spatial regulation of CaMKII signaling. Although the overall cellular content and phosphorylation of CaMKII were reduced, its intracellular distribution and local activation are likely altered. Selective activation of oxCaMKII within the highly restricted dyadic cleft microdomain places the active kinase in close proximity to ryanodine receptors (RyR2), enabling highly localized modulation of calcium release [39]. Such compartmentalized signaling provides a plausible explanation for how relatively modest biochemical changes can produce marked electrophysiological consequences. Rather than requiring widespread activation of the entire cellular CaMKII pool, localized oxidative activation within strategic microdomains may be sufficient to promote spontaneous SR Ca^2+^ leak, triggered activity, and ultimately ventricular electrical instability.

Consistent with this mechanism, hypothyroid cardiomyocytes exhibited a marked increase in cytoplasmic ROS despite a reduction in mitochondrial ROS production, indicating that mitochondria are unlikely to represent the primary source of oxidative stress in this model. Instead, our pharmacological and biochemical findings consistently identified eNOS as the predominant source of ROS generation [40]. Pharmacological inhibition with L-NIO normalized ROS levels, identifying eNOS as an important source of ROS in hypothyroid cardiomyocytes. Although total eNOS and nNOS expression remained unchanged, reduced eNOS phosphorylation and a decreased dimer/monomer ratio provided evidence of eNOS uncoupling [26]. Under these conditions, electron transfer within eNOS becomes uncoupled from l-arginine oxidation, favoring superoxide generation at the expense of nitric oxide (NO) production. Thus, rather than functioning as a cardioprotective source of NO, uncoupled eNOS becomes an important driver of oxidative stress and electrical instability.

Consistent with impaired eNOS function, myocardial nitrite levels were significantly reduced, indicating diminished NO bioavailability. Although nitrite represents an indirect index of NO metabolism, these findings closely agree with our DAF-FM measurements demonstrating reduced intracellular NO together with markedly elevated cytoplasmic ROS. Collectively, these complementary observations establish a coordinated shift in eNOS function from physiological NO production toward pathological superoxide generation, a hallmark of eNOS uncoupling.

Thyroid hormones are well established activators of the PI3K/Akt signaling pathway, which promotes phosphorylation of eNOS at Ser1177 and maintains NO bioavailability [41], [42]. Accordingly, the simultaneous reduction in total Akt, phosphorylated Akt, and phosphorylated eNOS observed in the present study strongly suggests that impaired Akt-dependent signaling represents an upstream event leading to eNOS uncoupling. Rather than acting as independent alterations, reduced Akt activity, impaired eNOS phosphorylation, diminished NO bioavailability, and increased ROS generation appear to constitute a single interconnected signaling cascade linking thyroid hormone deficiency to oxidative stress and electrical instability.

Importantly, oxidative activation of CaMKII did not restore physiological Akt signaling. Instead, oxCaMKII preferentially reinforced redox-dependent remodeling while dissociating oxidative signaling from the classical Ca^2+^/calmodulin-dependent activation pathway [43], [44]. This distinction is particularly relevant because it indicates that oxidative stress does not simply replace physiological CaMKII activation but fundamentally redirects kinase signaling toward a proarrhythmic phenotype.

The causal relationship between eNOS uncoupling and CaMKII oxidation was further supported by BH4 experiments. The likely restoration of eNOS coupling could reduce myocardial oxCaMKII levels, suggesting that CaMKII oxidation is downstream of eNOS dysfunction rather than an isolated consequence of hypothyroidism. This finding provides direct mechanistic evidence linking impaired NO signaling to redox-dependent CaMKII activation and further strengthens our proposed pathway in which eNOS uncoupling drives oxidative stress.

Functionally, restoration of eNOS coupling with BH_4_ or pharmacological scavenging of ROS with NAC markedly reduced stress-induced ventricular arrhythmias in vivo. Importantly, neither intervention normalized basal ECG parameters, including PR and QTc intervals, indicating that persistent ion channel remodeling remained despite attenuation of oxidative stress. These findings suggest that oxidative stress primarily functions as the trigger for ventricular arrhythmogenesis, whereas the underlying electrophysiological remodeling provides the arrhythmogenic substrate. Consequently, suppression of oxidative signaling is sufficient to markedly reduce arrhythmia susceptibility under pharmacological stress condition even in the presence of persistent electrical remodeling [45], [46]. Interestingly, NAC produced a greater antiarrhythmic effect than BH_4_. Although the precise mechanisms responsible for this difference remain to be established, the broader antioxidant properties of NAC may provide greater protection by simultaneously scavenging reactive oxygen species and replenishing intracellular glutathione stores [45], [46]. These observations further support the concept that oxidative stress is a central determinant of arrhythmogenic vulnerability in hypothyroidism.

From a clinical perspective, ventricular arrhythmias are relatively uncommon in hypothyroid patients under resting conditions, a pattern faithfully reproduced in our experimental model [29]. However, as described in several inherited and acquired channelopathies, electrical instability frequently becomes evident only under conditions of adrenergic stress [47], [48], [49]. The present findings therefore raise the possibility that conventional resting electrocardiography may underestimate arrhythmogenic risk in hypothyroid patients. Although further clinical investigation is clearly required, stress-based functional assessment may represent a useful strategy for identifying individuals with latent electrical vulnerability.

Collectively, the present study identifies eNOS uncoupling as a central mechanistic link between thyroid hormone deficiency and ventricular electrical instability. We propose a hierarchical pathway in which hypothyroidism impairs Akt-dependent eNOS signaling, promoting eNOS uncoupling, reduced NO bioavailability, excessive ROS generation, oxidative activation of CaMKII, disrupted calcium handling, and ultimately stress-induced ventricular arrhythmias. By integrating electrophysiological, biochemical, molecular, and pharmacological evidence, our findings substantially advance the current understanding of the pathophysiology of hypothyroidism-associated arrhythmias and identify restoration of eNOS coupling as a promising therapeutic strategy for reducing arrhythmogenic susceptibility.

## CRediT authorship contribution statement

**Diego Santos Souza:** Conceptualization, Data curation, Formal analysis, Funding acquisition, Supervision, Writing – original draft, Writing – review & editing. **Leisiane Pereira Marques:** Formal analysis, Methodology, Data curation, Writing – original draft, Writing – review & editing. **Aimée Obolari Durço:** Data curation, Formal analysis, Writing – original draft, Writing – review & editing. **Fabiana da Silva Alcântara:** Data curation, Writing – original draft, Formal analysis. **Diego José Belato Orts:** Data curation, Formal analysis, Writing – original draft. **Melissa Tainan Silva Dias:** Data curation, Formal analysis. **Michael Ramon de Lima Conceiçao:** Data curation, Formal analysis. **Jorge Lucas Teixeira-Fonseca:** Data curation, Formal analysis. **Luiz Fernando Santos Matos:** Data curation, Formal analysis. **Maria Luiza Alarcon Mady Barbosa:** Data curation, Formal analysis. **Daiara Custódio Santos:** Data curation, Formal analysis. **Luciano dos Santos Aggum Capettini:** Data curation, Formal analysis, Methodology. **Carlos Roberto Prudencio:** Data curation, Funding acquisition. **Danilo Lustrino:** Data curation, Formal analysis, Writing – original draft. **Jader Santos Cruz:** Data curation, Methodology, Writing – original draft, Writing – review & editing. **Danilo Roman-Campos:** Conceptualization, Formal analysis, Funding acquisition, Investigation, Methodology, Writing – original draft, Writing – review & editing.

## Data availability

The datasets generated and/or analyzed during the current study are available from the corresponding author upon reasonable request.

## Funding

This work was supported by the 10.13039/501100001807São Paulo Research Foundation [10.13039/501100001807FAPESP #s2021/05584-7 and 2020/14635-1 to DRC]. DSS, AOD, LPM, FSA, DJBO, MRLC, JLTF holds a Fellowship from 10.13039/501100001807FAPESP [grant #2019/18918-0, #2023/11912-2, #2021/15122-0, #2023/14219-6, #2022/07256-0, #2024/06444-2, and #2022/14133-1 respectively]. 10.13039/501100004901Fundação de Amparo à Pesquisa do Estado de Minas Gerais [LSAC – grant APQ-01901-21]. 10.13039/501100003593Conselho Nacional de Desenvolvimento Científico e Tecnológico (CNPq) (DSS #409449/2024-5 and #318573/2025-3; DRC #310112/2023-0; DL #14403404/2025-8, #300363/2025-7, and #443962/2024-3). 10.13039/501100003593CNPq/Decit/MS (DSS 405631/2024-3; 406492/2024-7). JSC is 10.13039/501100003593CNPq Research Fellow (Grant #312939/2021-3).

## Declaration of competing interest

The authors declare that they have no known competing financial interests or personal relationships that could have appeared to influence the work reported in this paper.
