# Supplementary material for: eNOS uncoupling drives oxidative stress and arrhythmogenic vulnerability in experimental hypothyroidism
Source: Redox Biol. 2026 Oct 20;98:104447. doi: 10.1016/j.redox.2026.104447 (PMC13720376; doi:10.1016/j.redox.2026.104447)
Supplement: Supplementary file 1 [file mmc3.pdf]

Original Western blot Figure 3Bi

CaMKII total - Ponceau of representative western blot

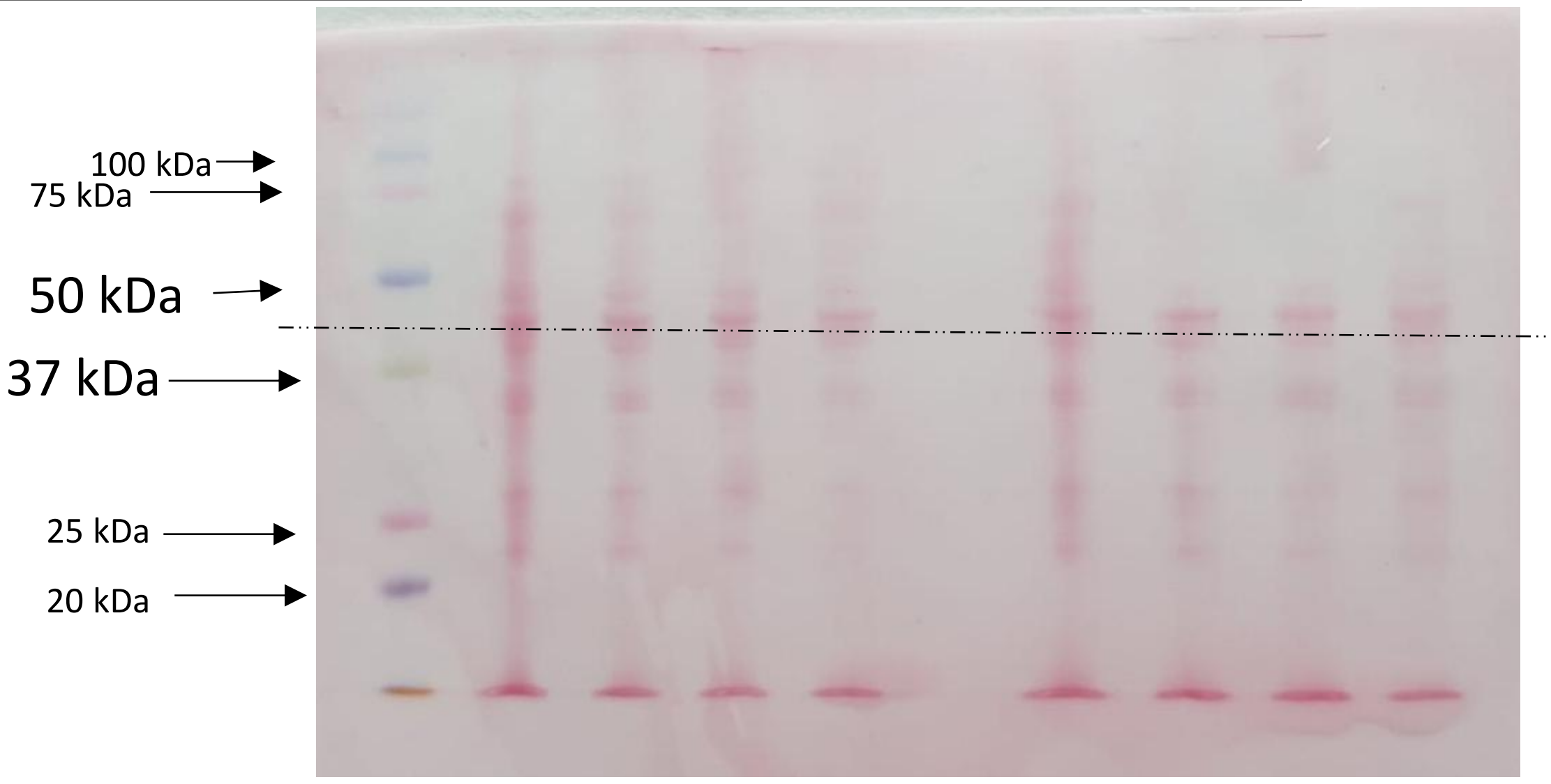

CaMKII total representative western blot

C = Control
H = Hipo

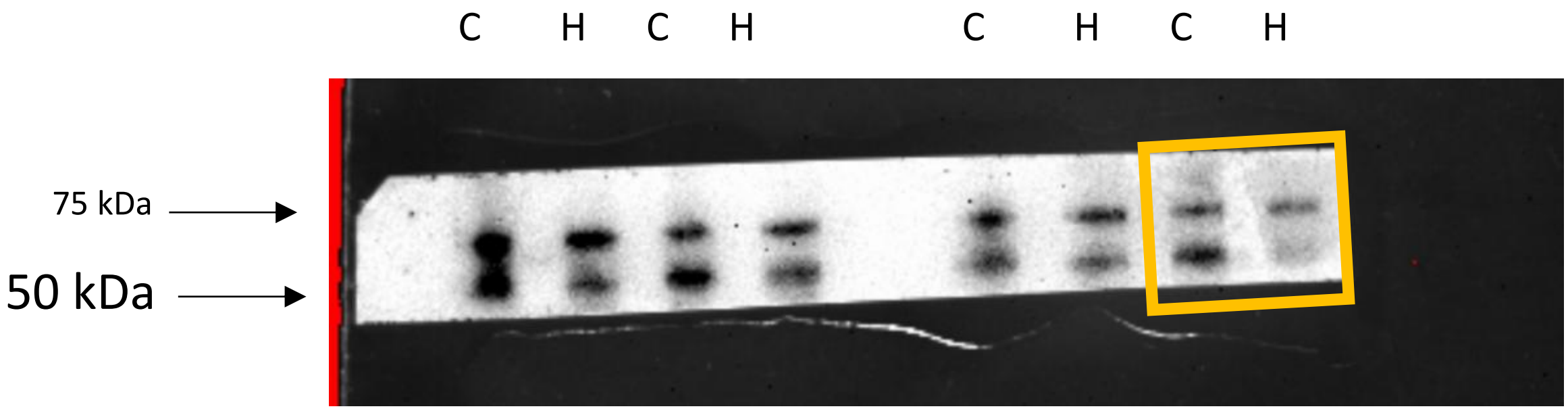

C = Control
H = Hipo

GAPDH

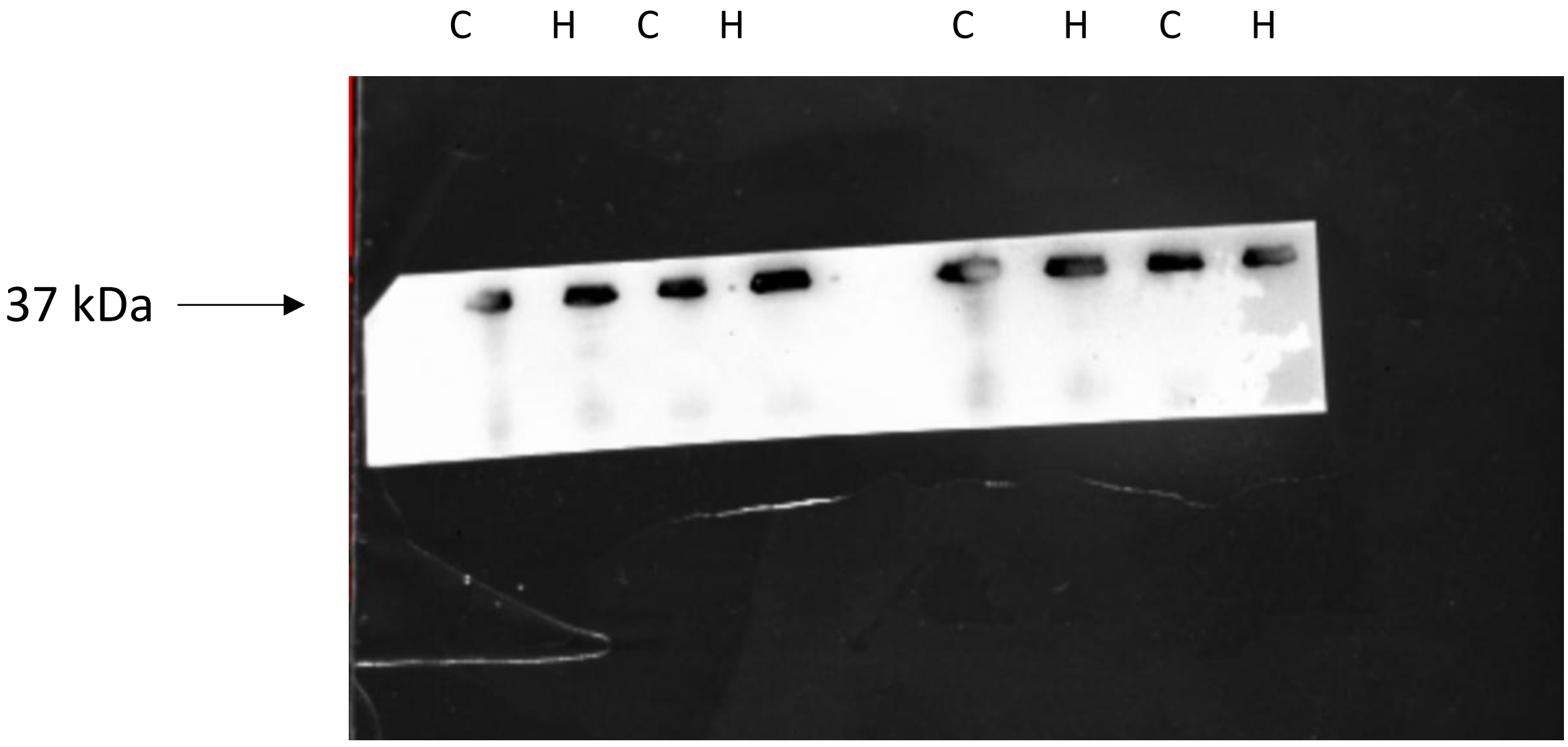

Chemiluminescence for immunodetection detected with iBrigt CL1000 (Thermo Fisher Scientific)

anti-GAPDH 1:1000, #32233, Santa Cruz Biotechnology;
anti-CaMKII 1:500, #5306, Santa Cruz Biotechnology

Original Western blot Figure 3Bi

Ponceou

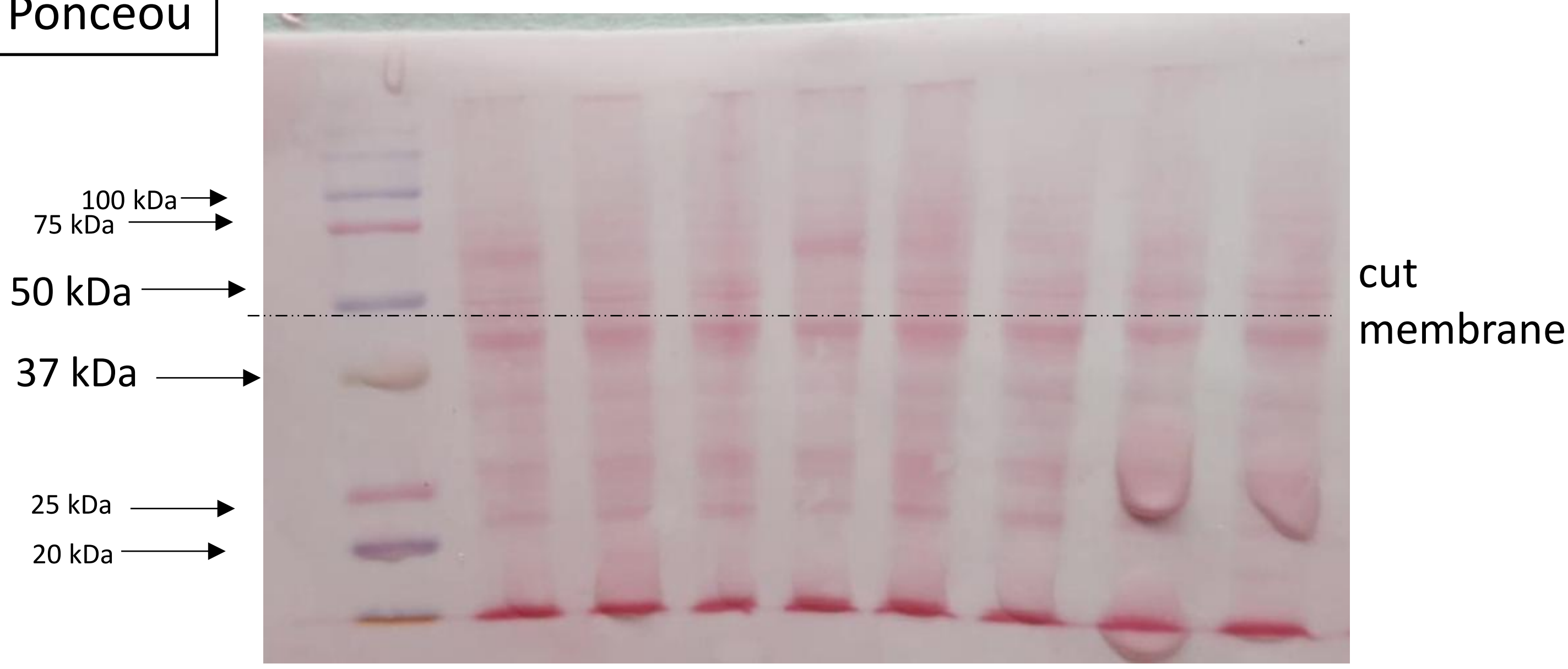

CaMKII total

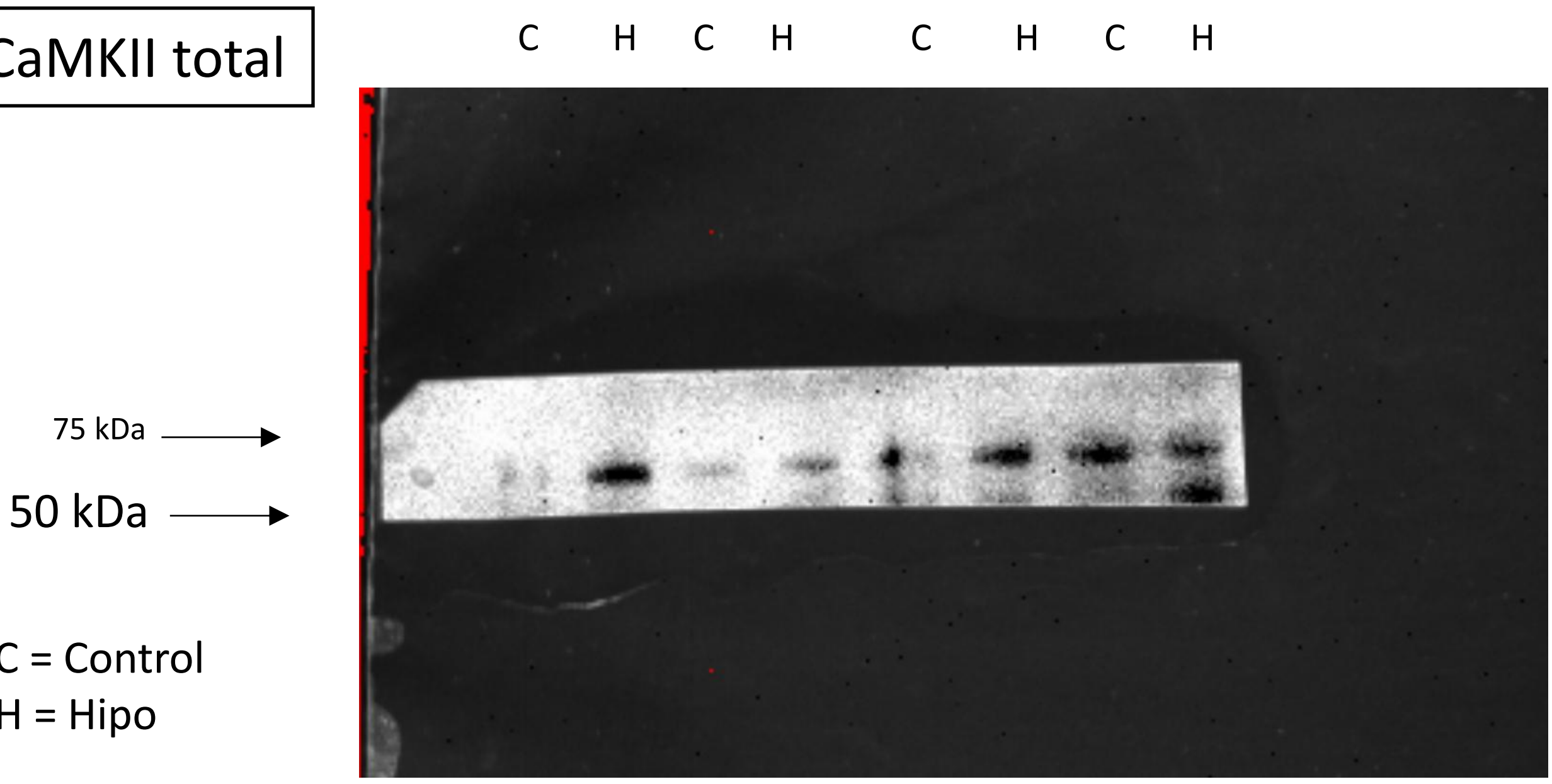

C = Control
H = Hipo

C = Control
H = Hipo

GAPDH

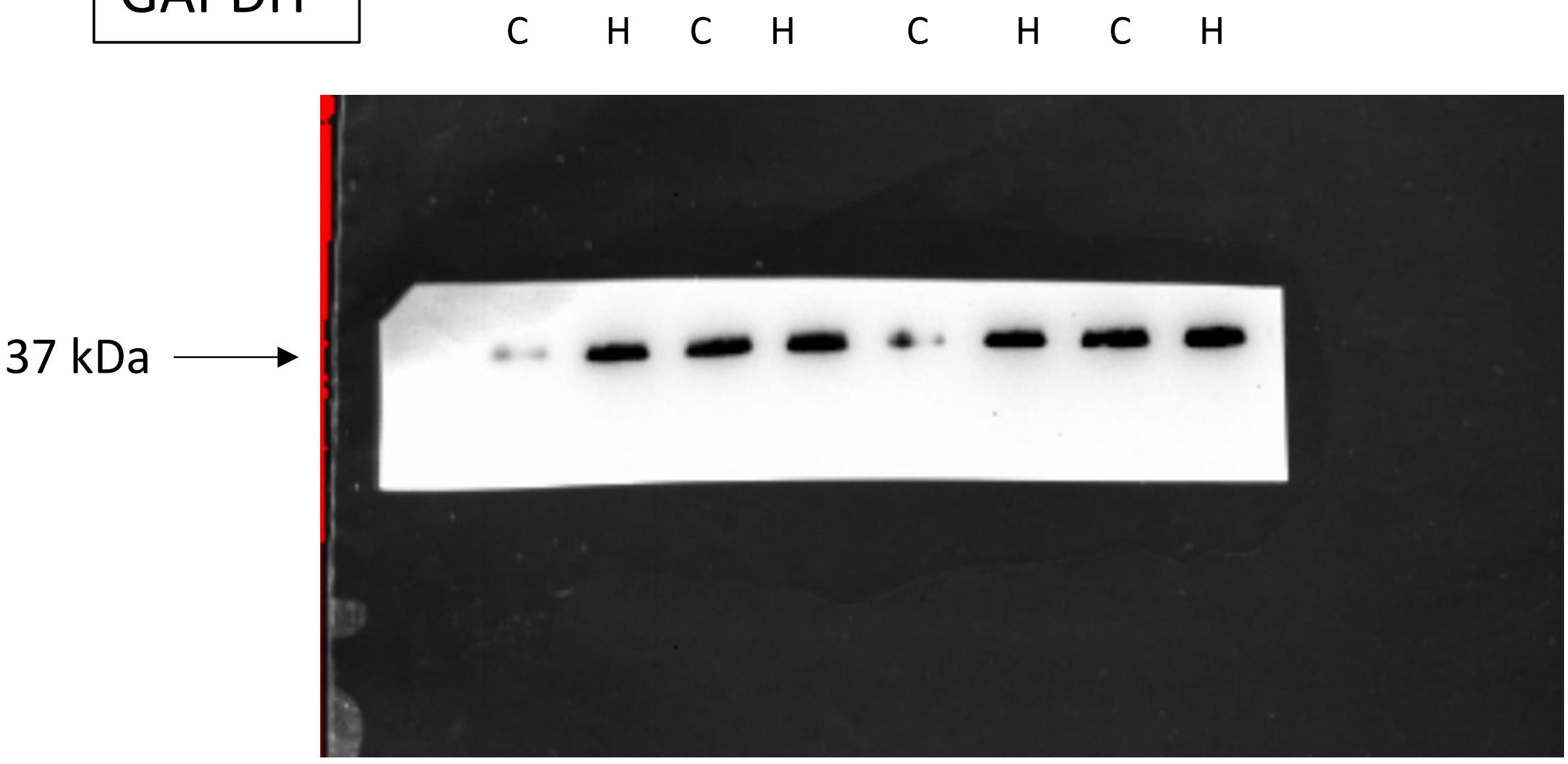

Chemiluminescence for immunodetection detected with iBrigt CL1000 (Thermo Fisher Scientific)

anti-GAPDH 1:1000, #32233, Santa Cruz Biotechnology;
anti-CaMKII 1:500, #5306, Santa Cruz Biotechnology

Original Western blot Figure 3Biii

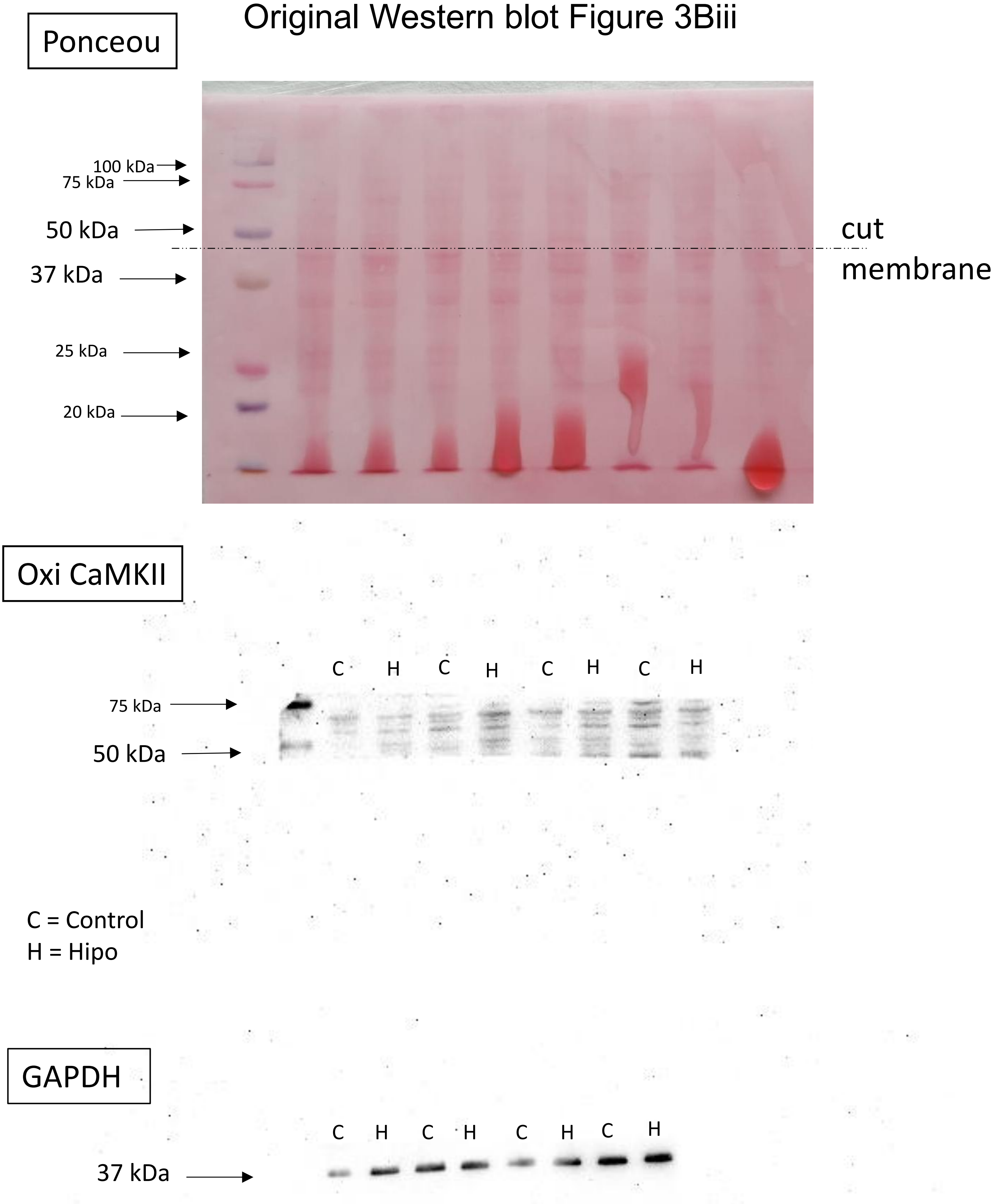

Chemiluminescence for immunodetection detected with LAS 4000 equipment (GE HealthCare Life Science)

anti-GAPDH 1:1000, #32233, Santa Cruz Biotechnology;
anti-Oxi-CaMKII 1:1000, #071387, Millipore

Original Western blot Figure 3Biii

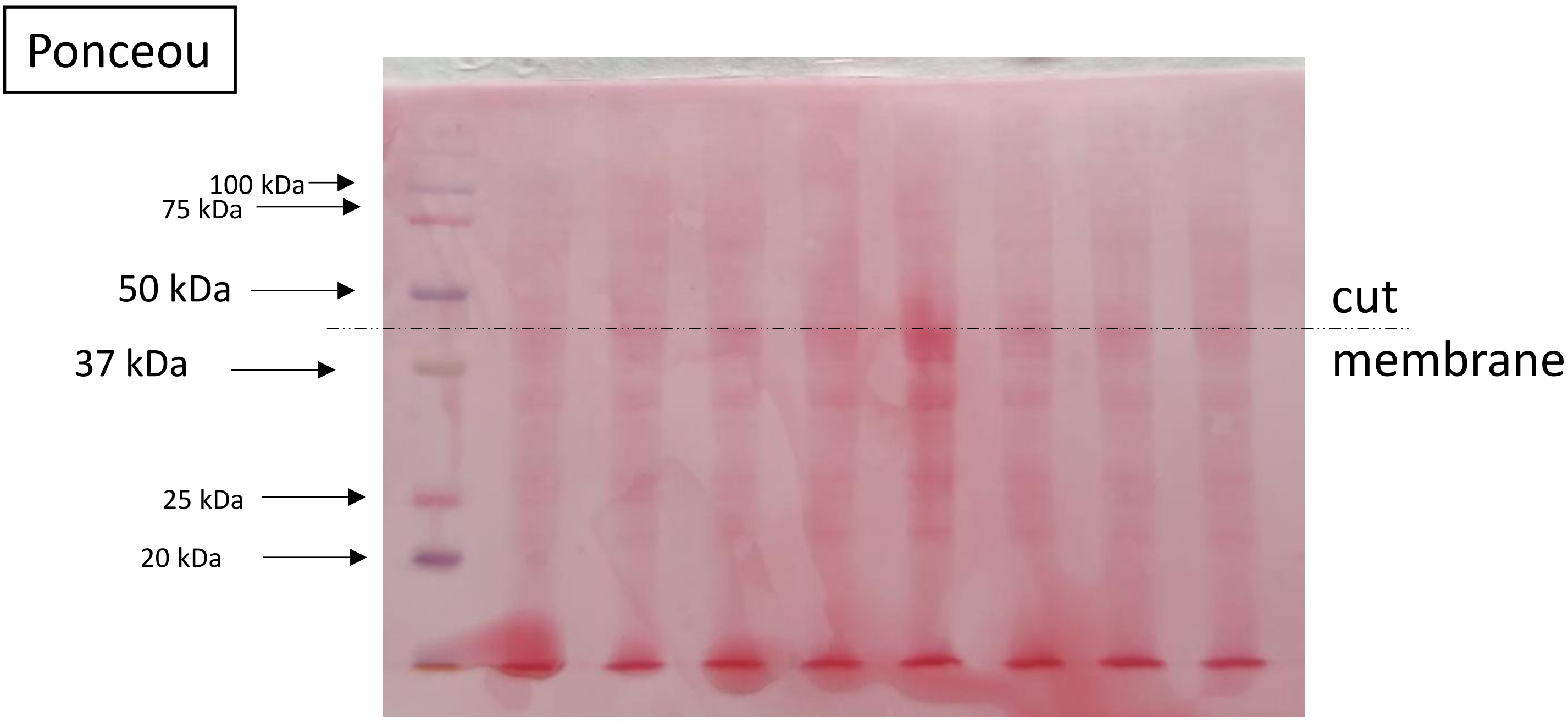

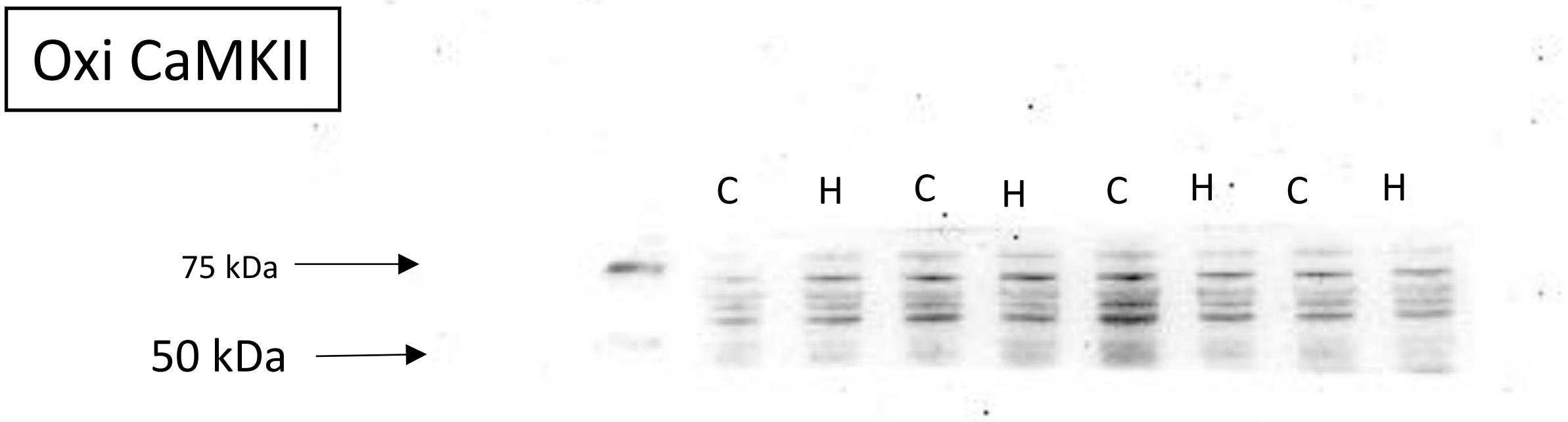

C = Control
H = Hipo

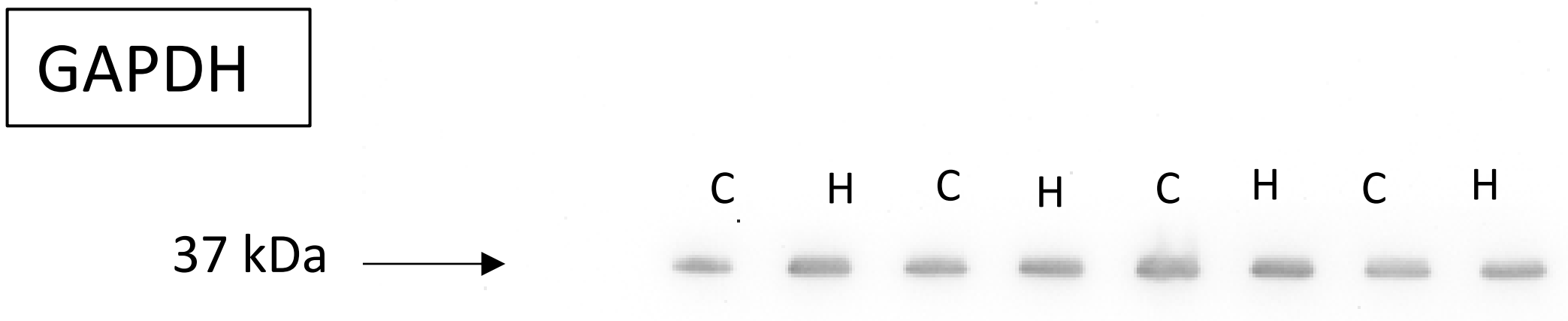

Chemiluminescence for immunodetection detected with LAS 4000 equipment (GE HealthCare Life Science)

anti-GAPDH 1:1000, #32233, Santa Cruz Biotechnology;
anti-Oxi-CaMKII 1:1000, #071387, Millipore

Original Western blot Figure 3Biii

Oxidized CaMKII - Ponceau of representative western blot

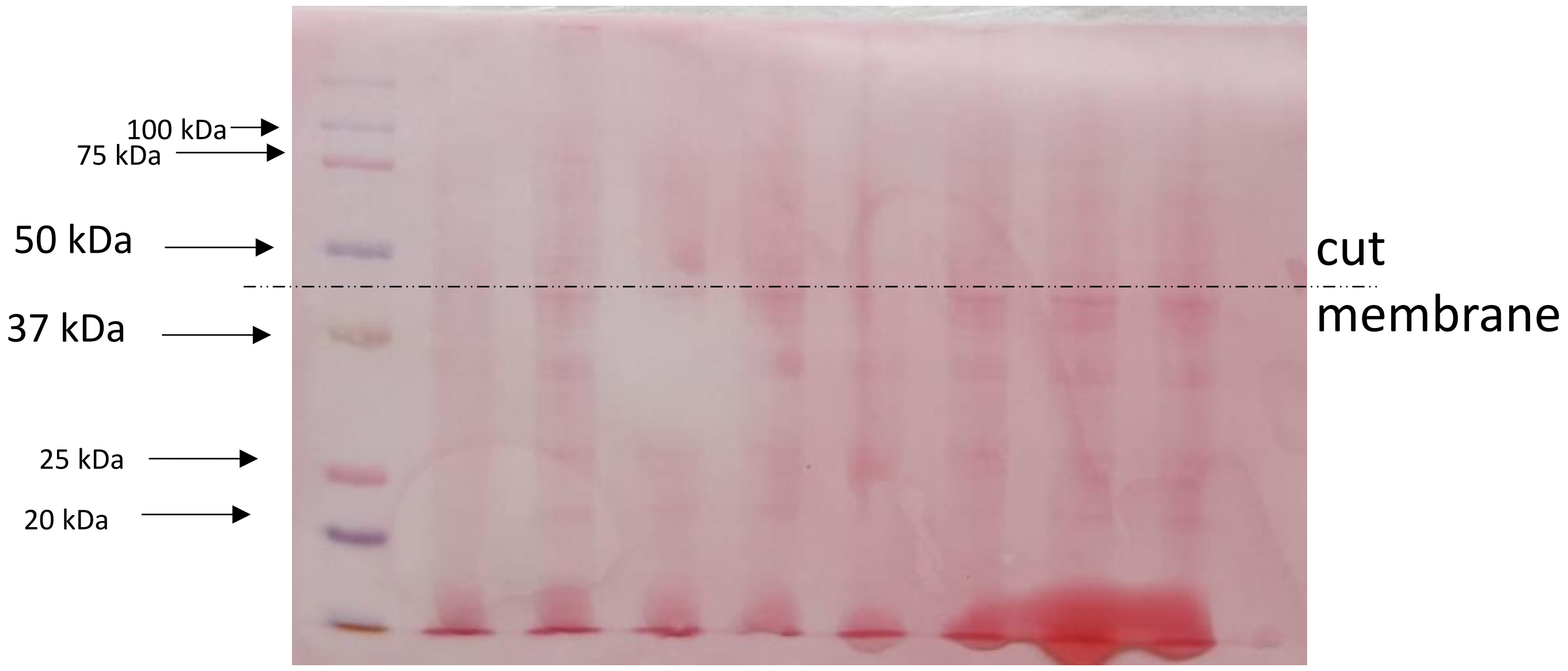

OxiCaMKII representative western blot

C = Control
H = Hipo

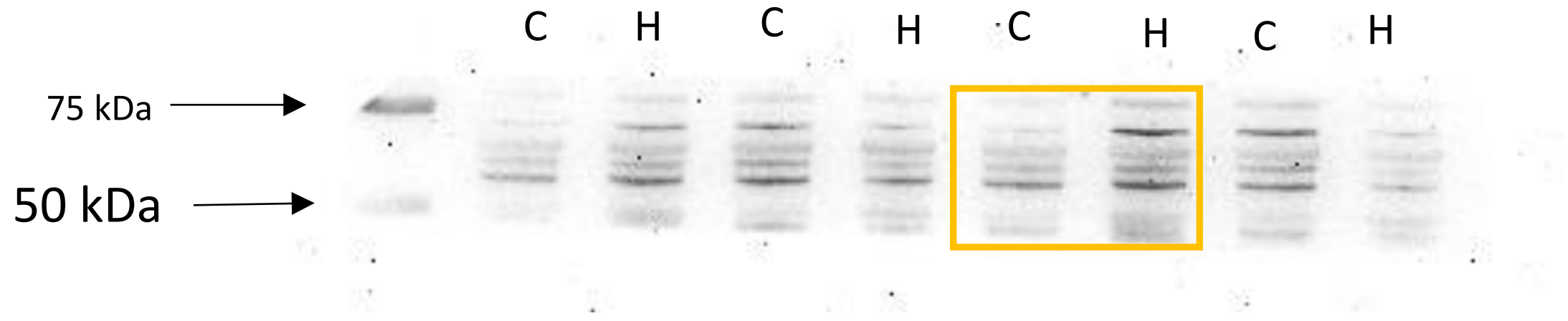

GAPDH

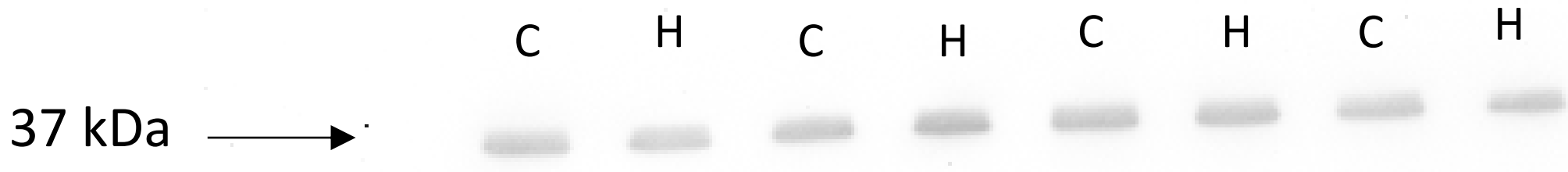

Chemiluminescence for immunodetection detected with LAS 4000 equipment (GE HealthCare Life Science)

anti-GAPDH 1:1000, #32233, Santa Cruz Biotechnology;
anti-Oxi-CaMKII 1:1000, #071387, Millipore

Original Western blot Figure 3Bii

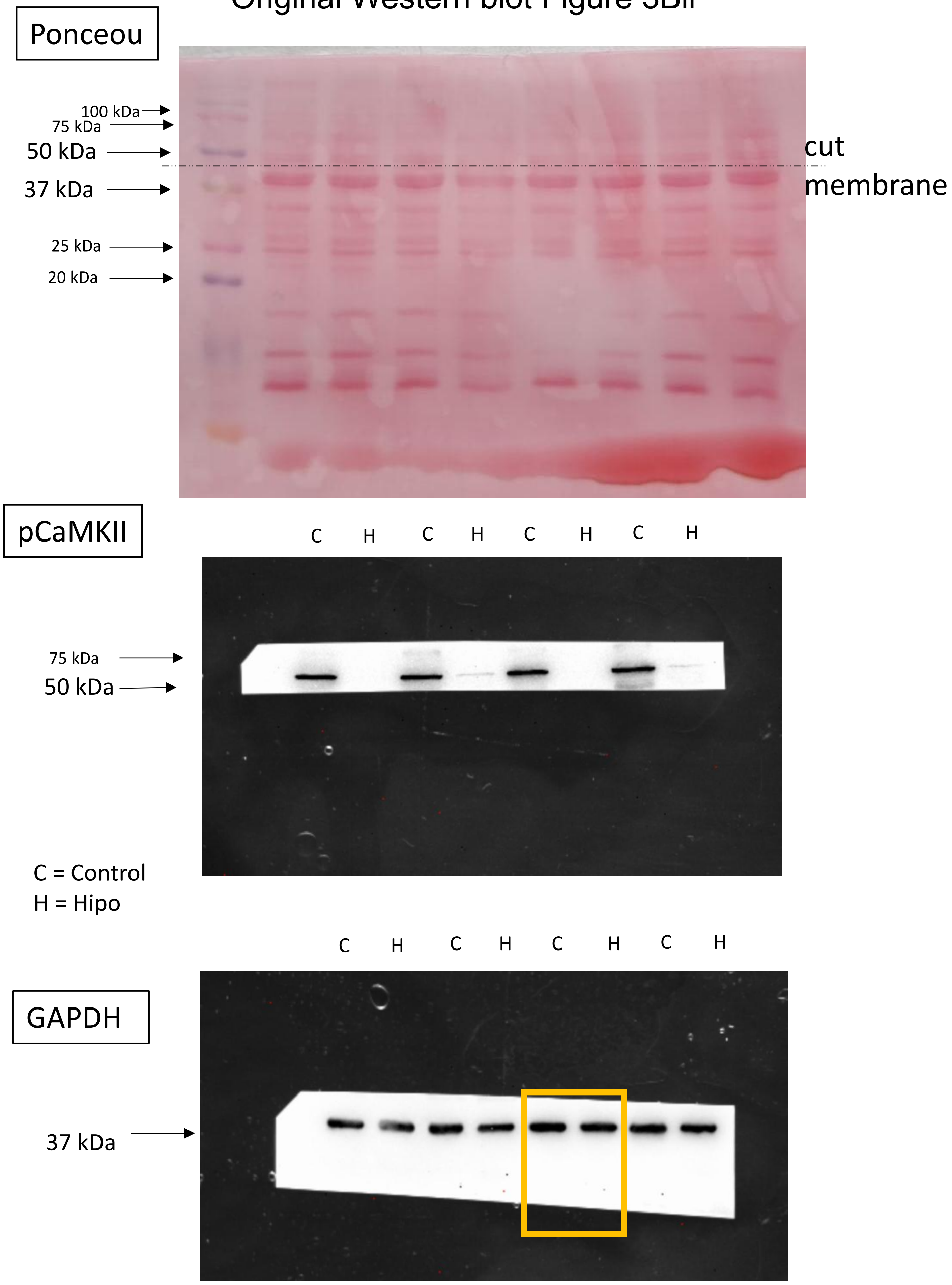

Chemiluminescence for immunodetection detected with iBrigt CL1000 (Thermo Fisher Scientific)

anti-GAPDH 1:1000, #32233, Santa Cruz Biotechnology;
anti-phospho-CaMKII 1:500, #4301561, Sigma Aldrich

Original Western blot Figure 3Bii

Phosphorylated CaMKII - Ponceau of representative western blot

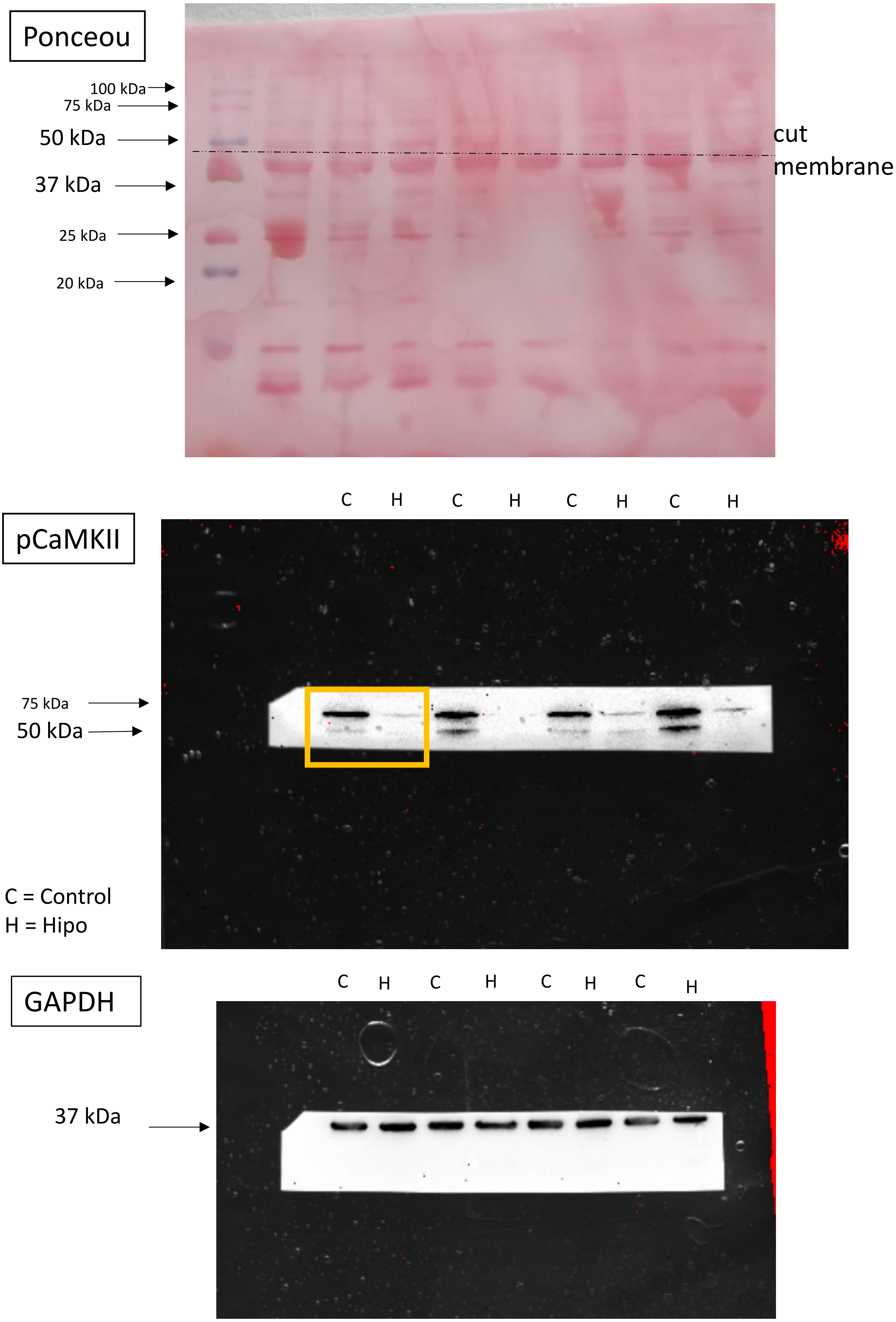

Chemiluminescence for immunodetection detected with iBrigt CL1000 (Thermo Fisher Scientific)

anti-GAPDH 1:1000, #32233, Santa Cruz Biotechnology;
anti-phospho-CaMKII 1:500, #4301561, Sigma Aldrich

Original Western blot Figure 5Ci

AKT total - Ponceau of representative western blot

Ponceou

75 kDa

50 kDa

37 kDa

25 kDa

20 kDa

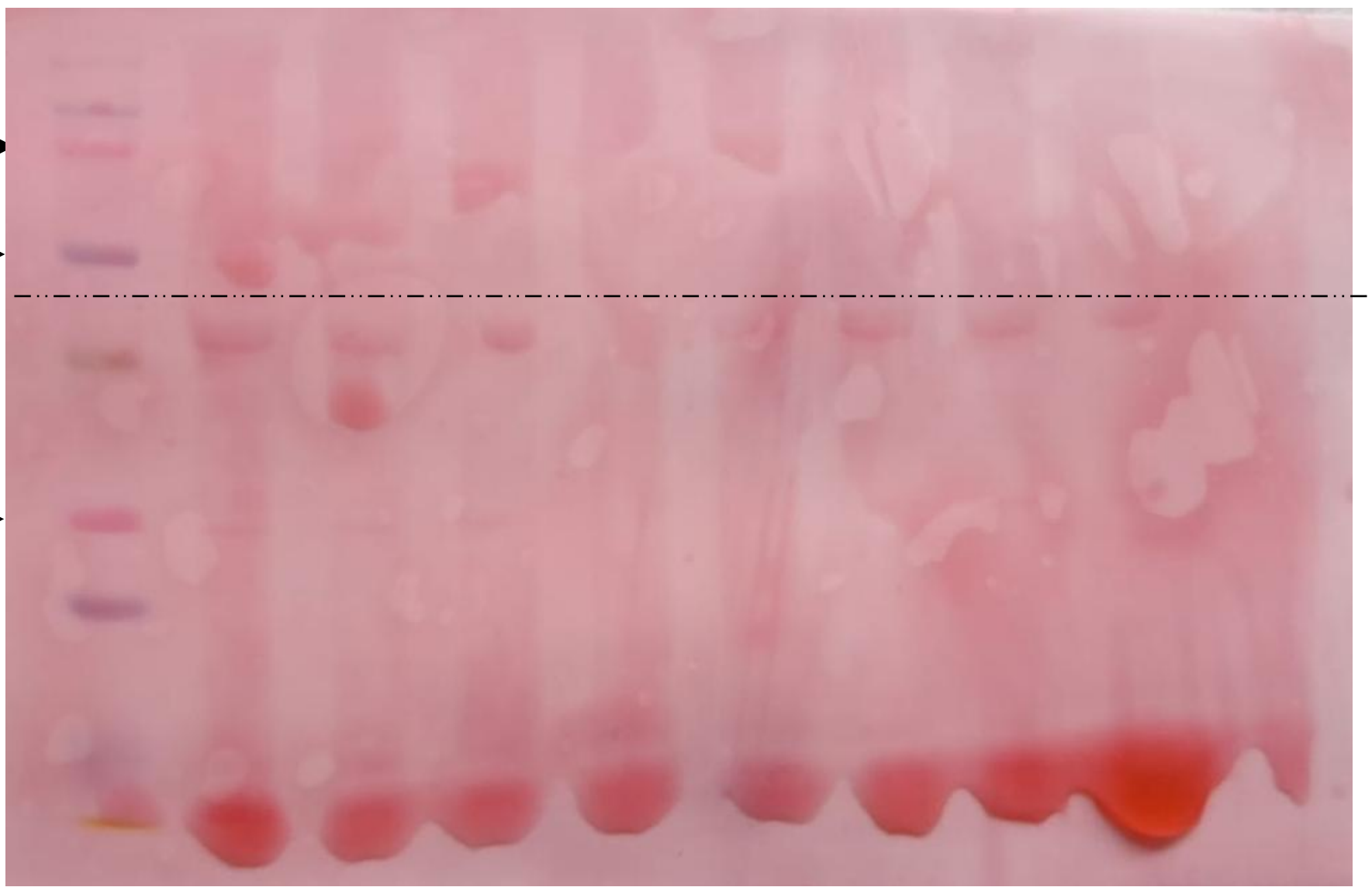

cut membrane

AKT total

C H C H C H C H

75 kDa

50 kDa

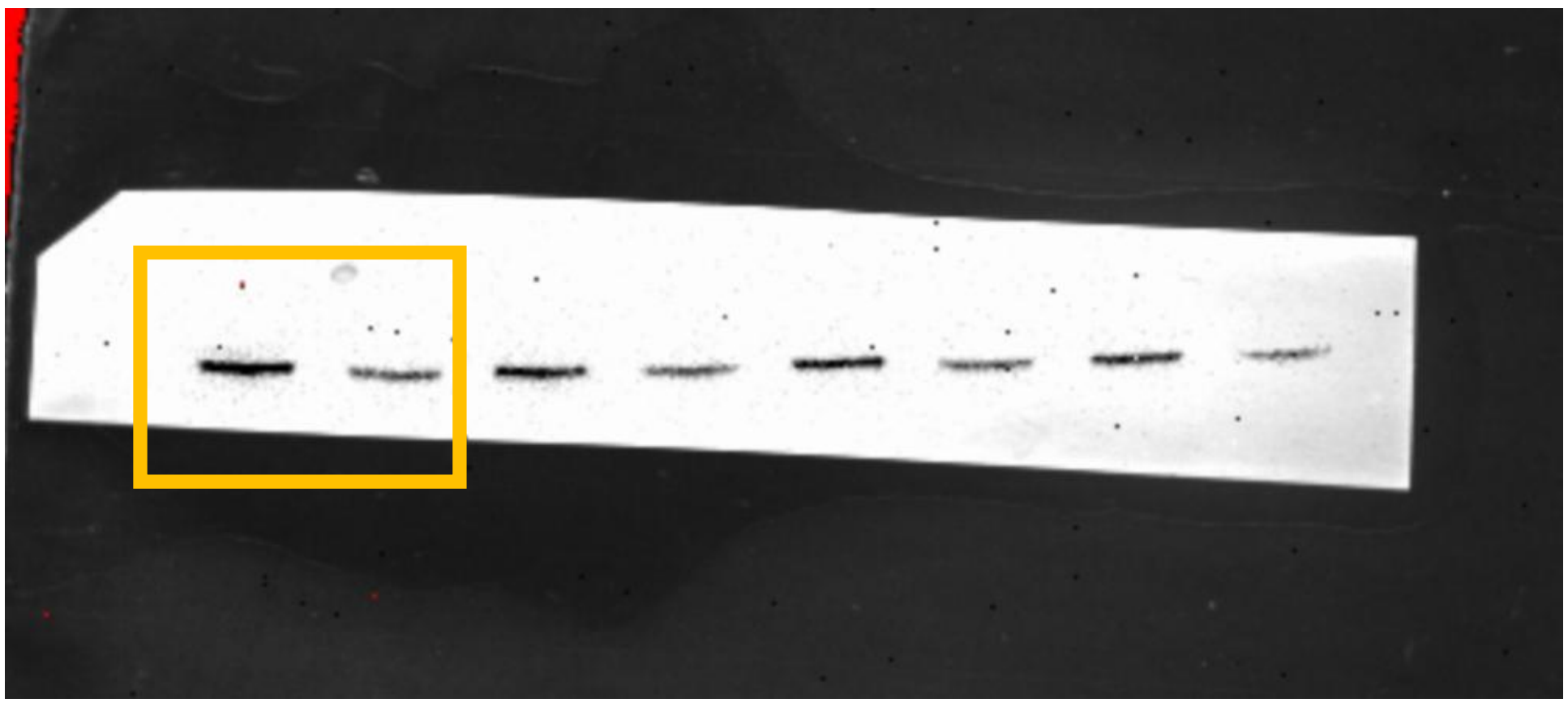

C = Control

H = Hipo

GAPDH

C H C H C H C H

37 kDa

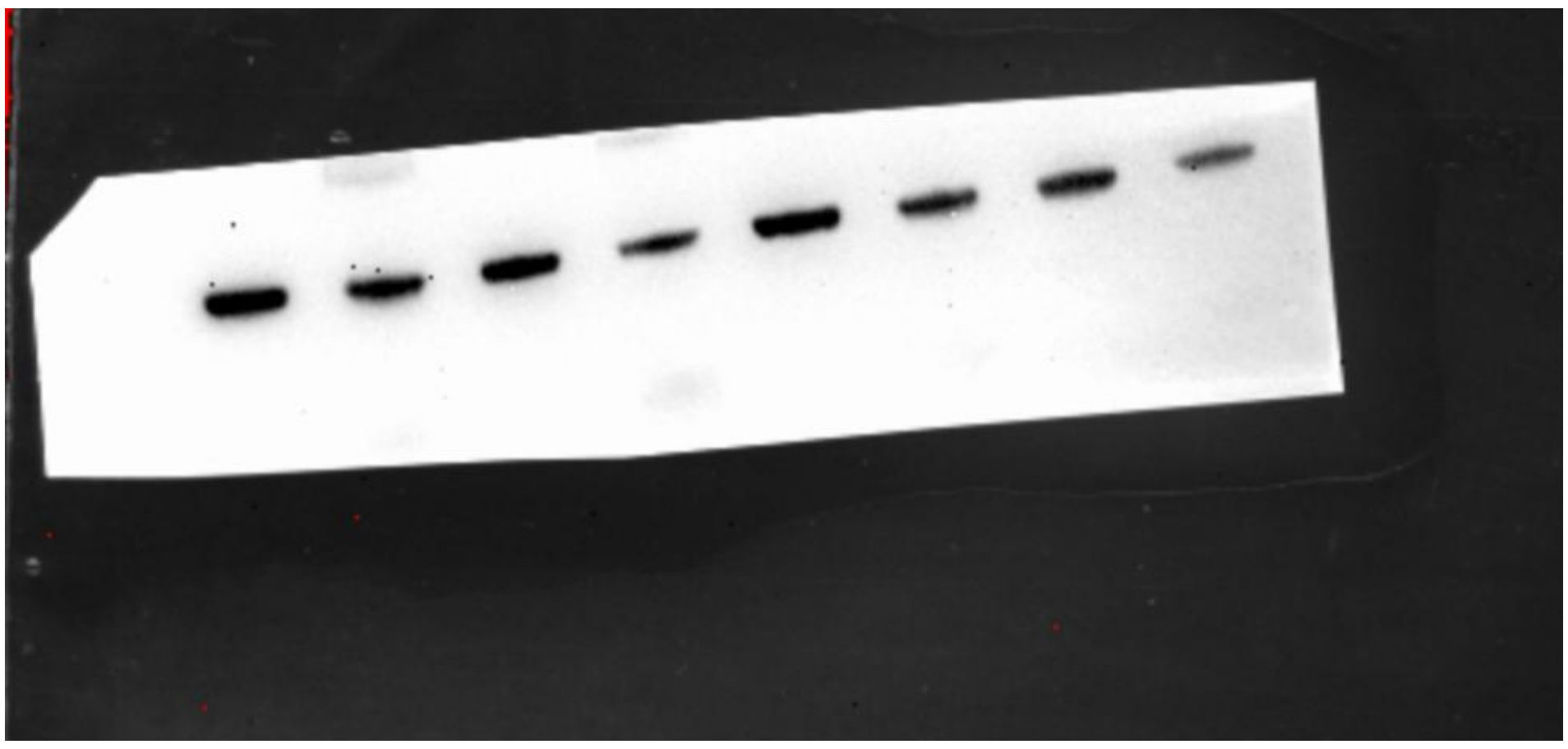

Chemiluminescence for immunodetection detected with iBrigt CL1000 (Thermo Fisher Scientific)

anti-GAPDH 1:1000, #32233, Santa Cruz Biotechnology;
anti-AKT 1:500, #9272S, Cell Signaling

Original Western blot Figure 5Ci

Ponceou

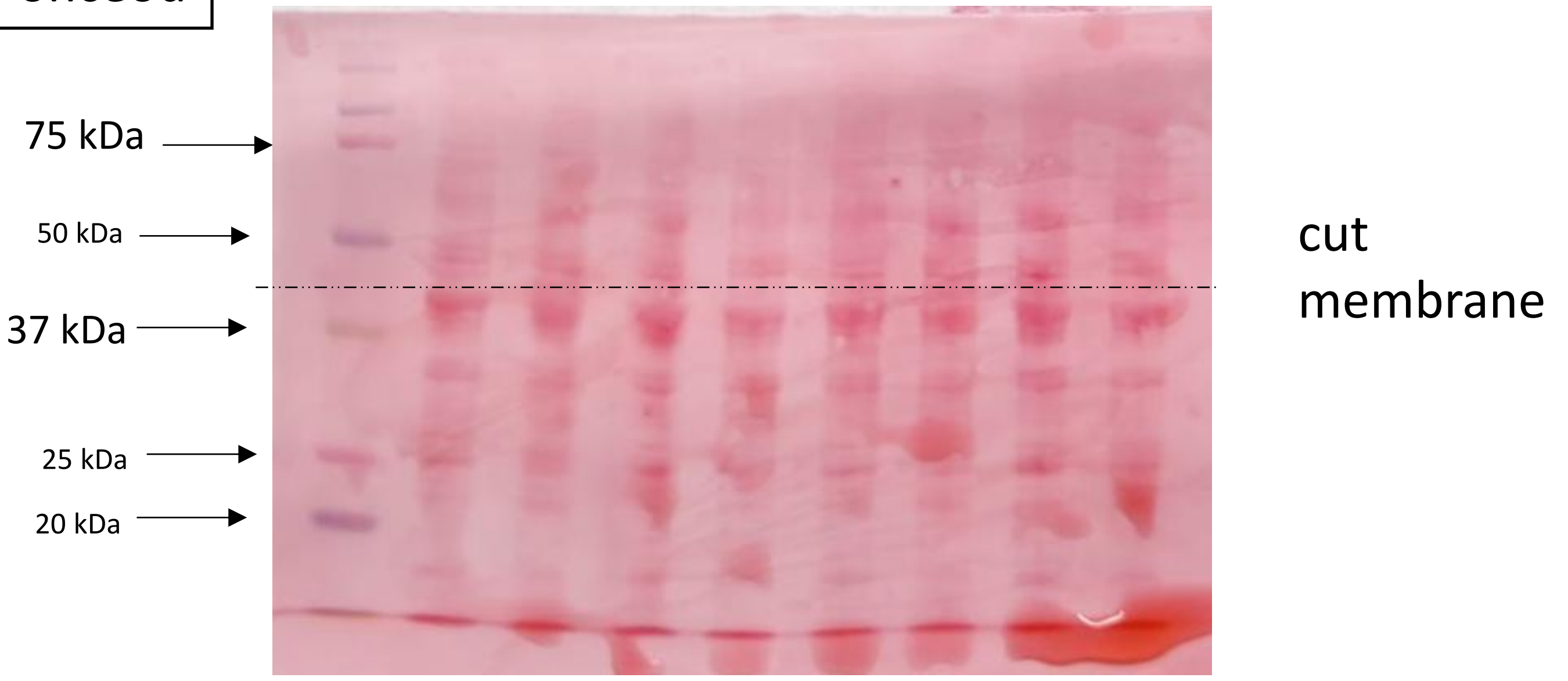

AKT total

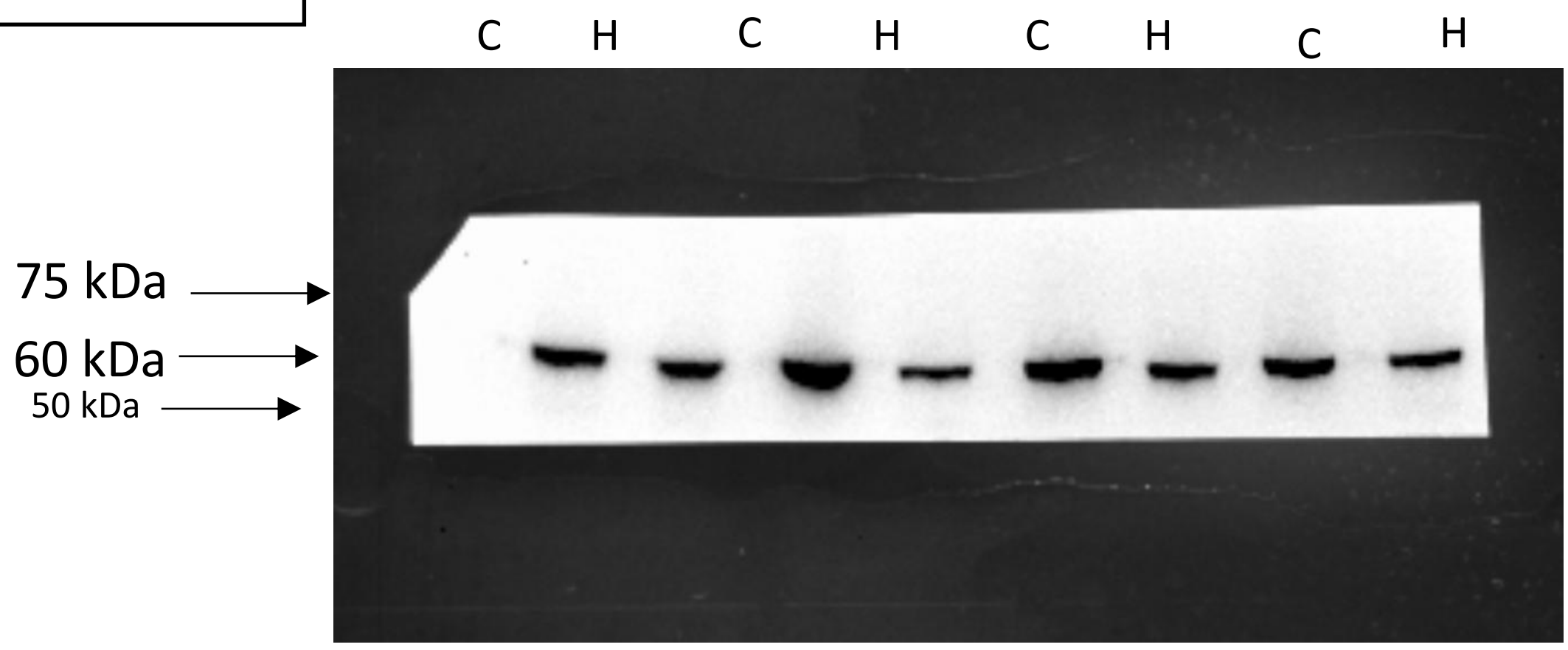

C = Control
H = Hipo

GAPDH

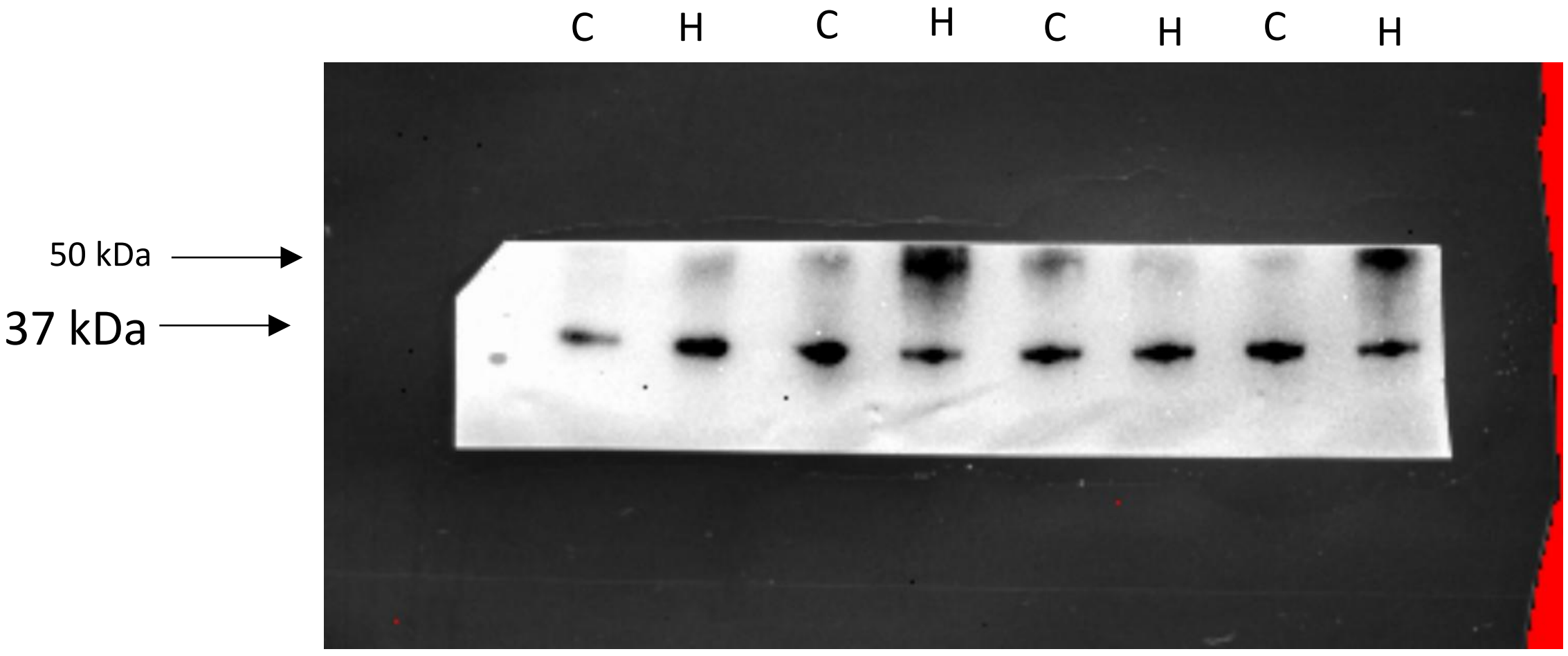

Chemiluminescence for immunodetection detected with iBrigt CL1000 (Thermo Fisher Scientific)

anti-GAPDH 1:1000, #32233, Santa Cruz Biotechnology;
anti-AKT 1:500, #9272S, Cell Signaling

Original Western blot Figure 5Cii

Phosphorylated AKT - Ponceau of representative western blot

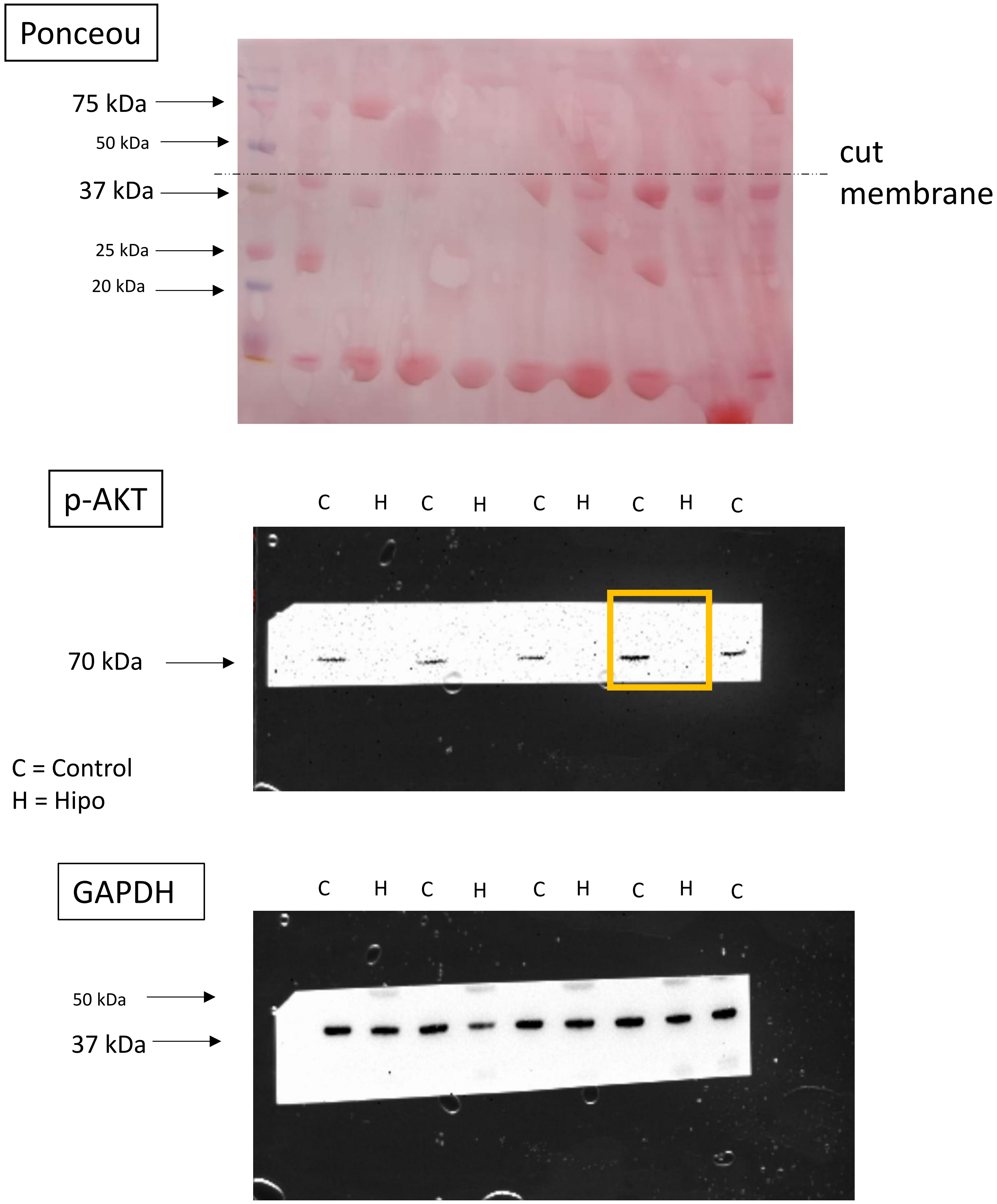

Chemiluminescence for immunodetection detected with iBrigt CL1000 (Thermo Fisher Scientific)

anti-GAPDH 1:1000, #32233, Santa Cruz Biotechnology; anti-pAKT 1:2000, #4060, Cell Signaling

Original Western blot Figure 5Cii

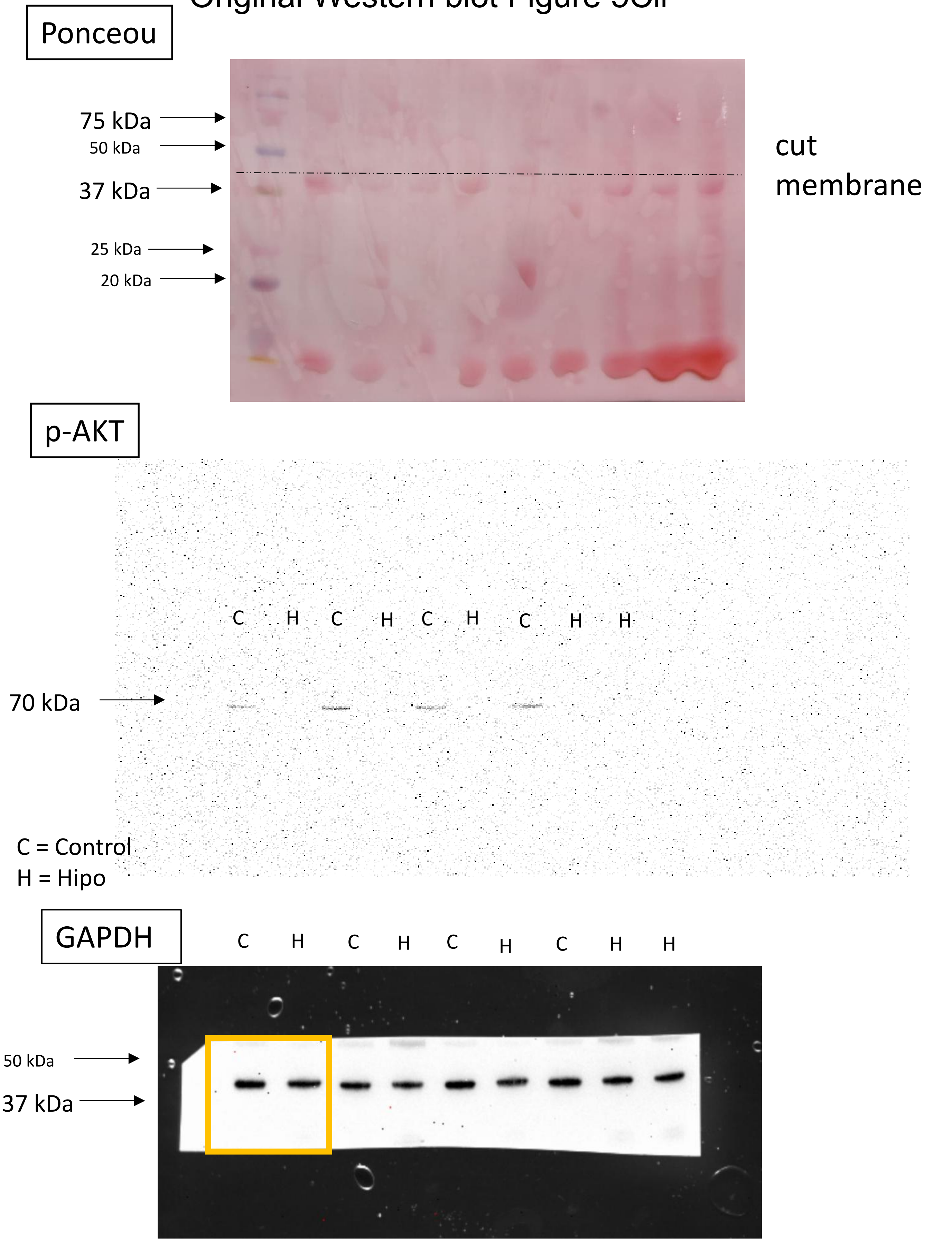

Chemiluminescence for immunodetection detected with iBrigt CL1000 (Thermo Fisher Scientific)

anti-GAPDH 1:1000, #32233, Santa Cruz Biotechnology;
anti-pAKT 1:2000, #4060, Cell Signaling

Original Western blot Figure 5Bi

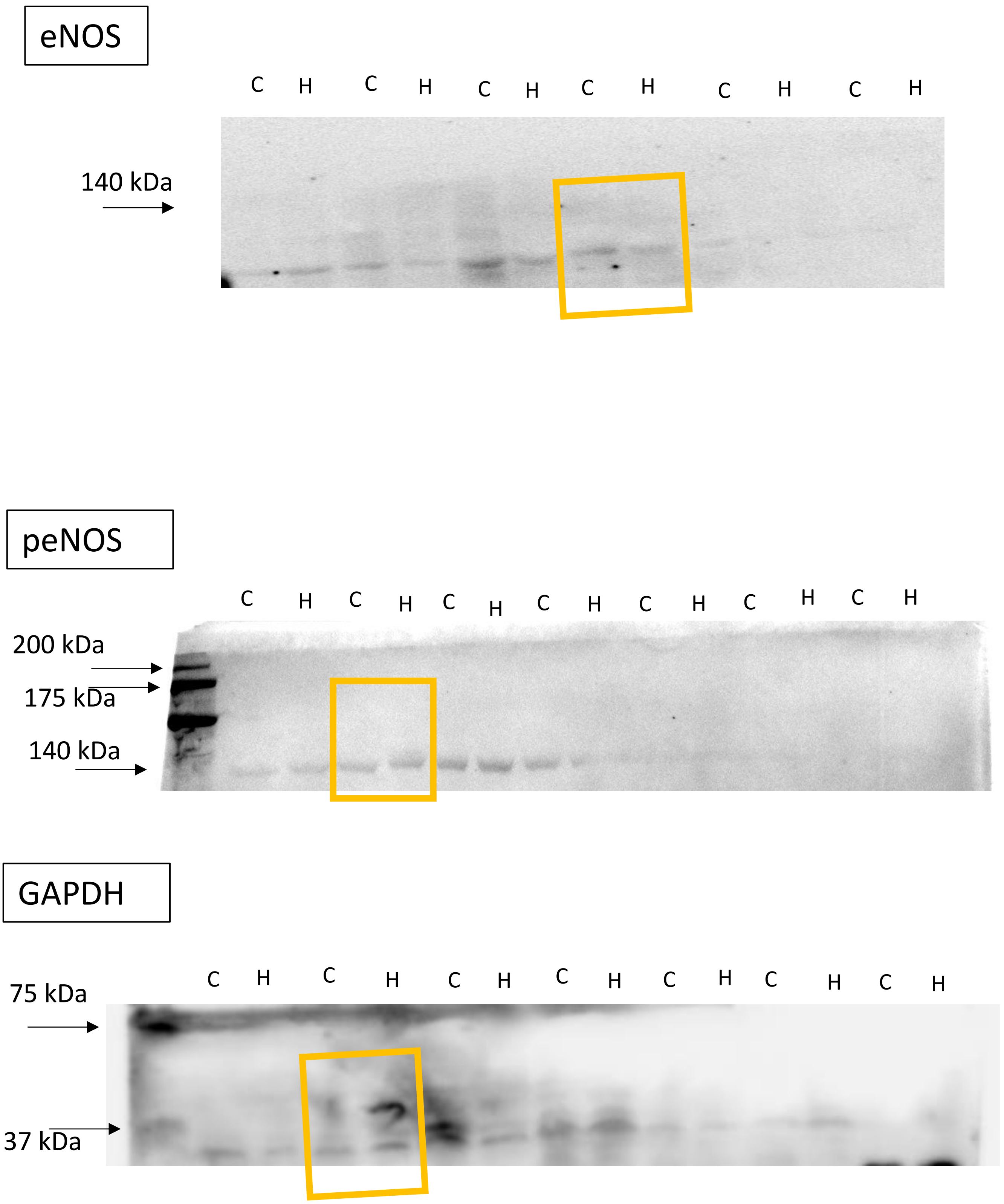

Ponceau staining was performed and is indicated in the corresponding experiments to confirm protein loading and transfer efficiency. However, images of the wells were not documented during this data acquisition; therefore, they are not available for inclusion.

Chemiluminescence for immunodetection detected with LAS 4000 equipment (GE HealthCare Life Science)

anti-eNOS 1:1000, sc-136977, Santa Cruz Biotechnology
anti-nNOS 1:1000, sc-8309, Santa Cruz Biotechnology
anti-GAPDH 1:1000, #32233, Santa Cruz Biotechnology

Original Western blot Figure 5B

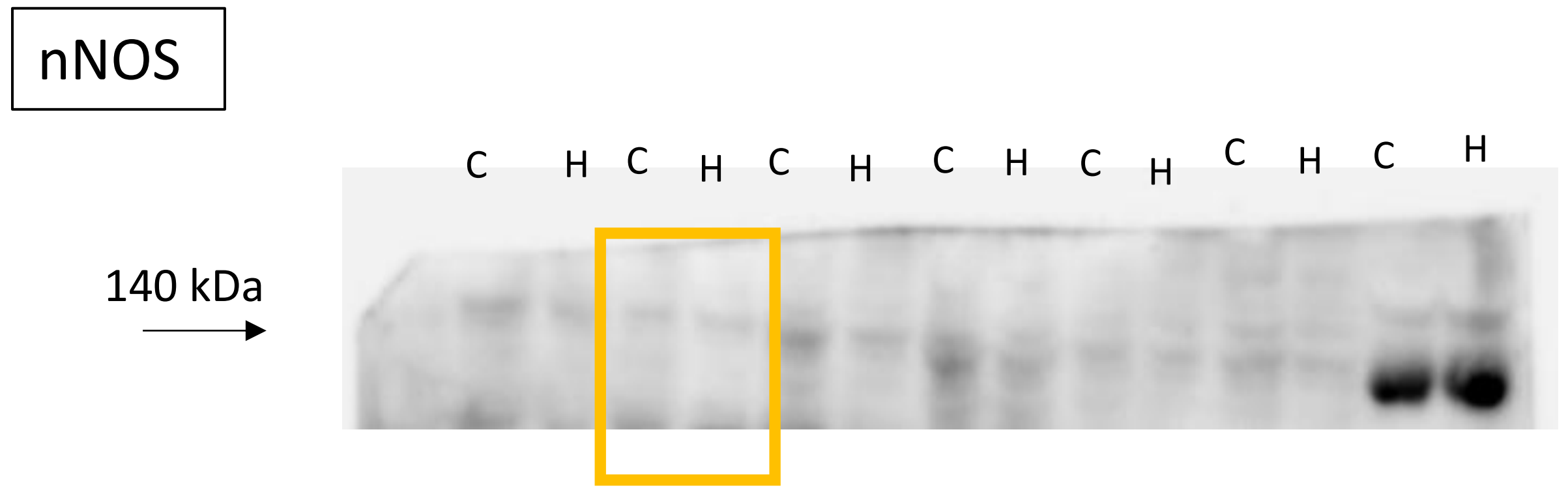

Original Western blot Figure 5Bii

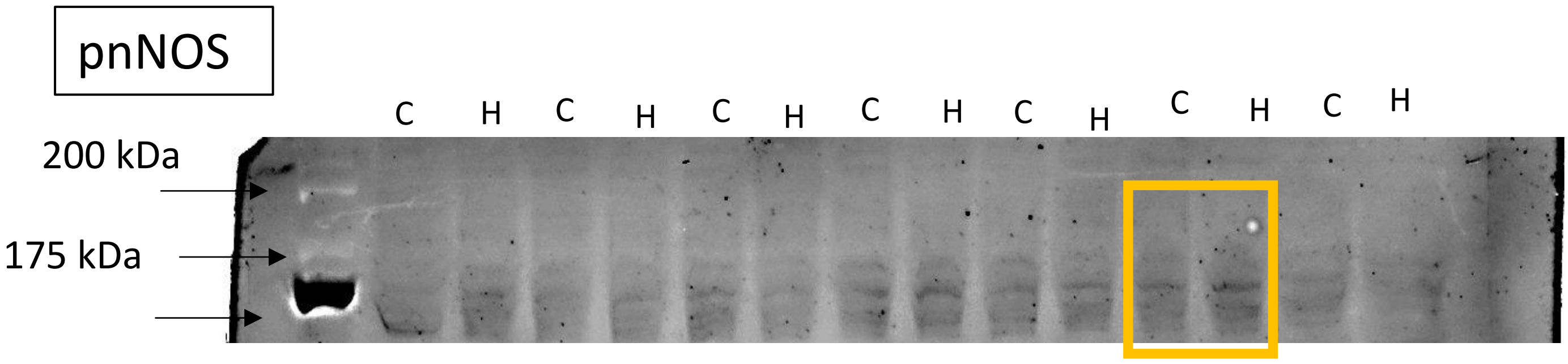

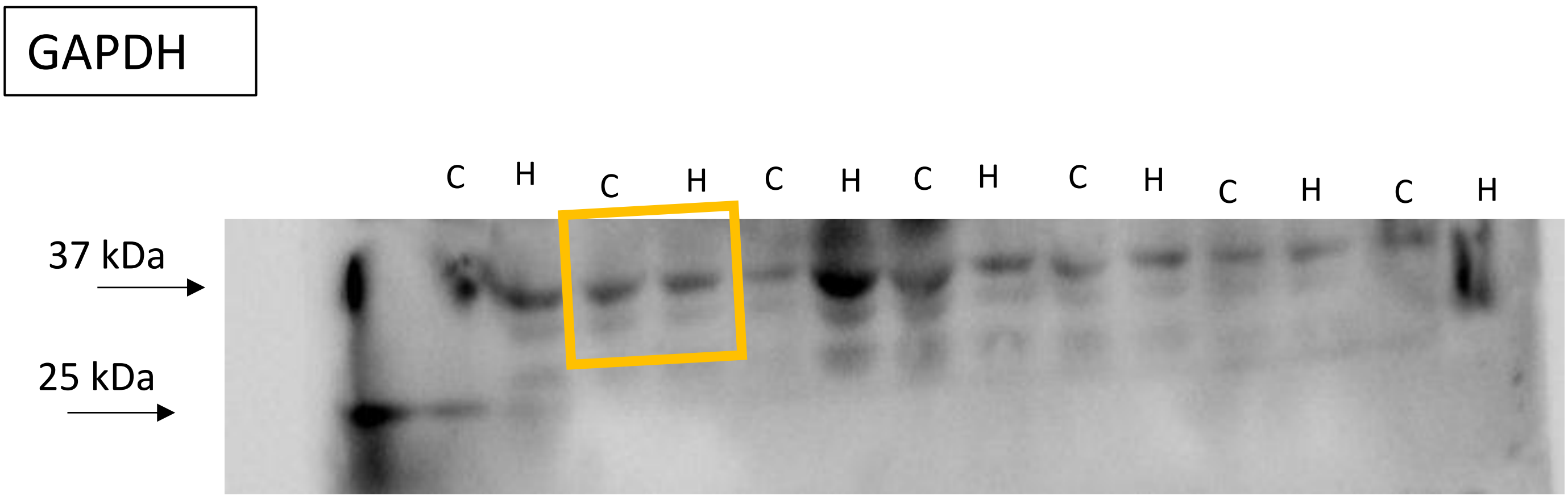

Ponceau staining was performed and is indicated in the corresponding experiments to confirm protein loading and transfer efficiency. However, images of the wells were not documented during this data acquisition; therefore, they are not available for inclusion.

Chemiluminescence for immunodetection detected with LAS 4000 equipment (GE HealthCare Life Science)

anti-pnNOSser852 1:1000; sc-19826, Santa Cruz Biotechnology
anti-peNOSser1177 1:1000; sc- 12972, Santa Cruz Biotechnology
anti-GAPDH 1:1000, #32233, Santa Cruz Biotechnology

Original Western blot Figure 6Ai

nNOS dimer / monomer

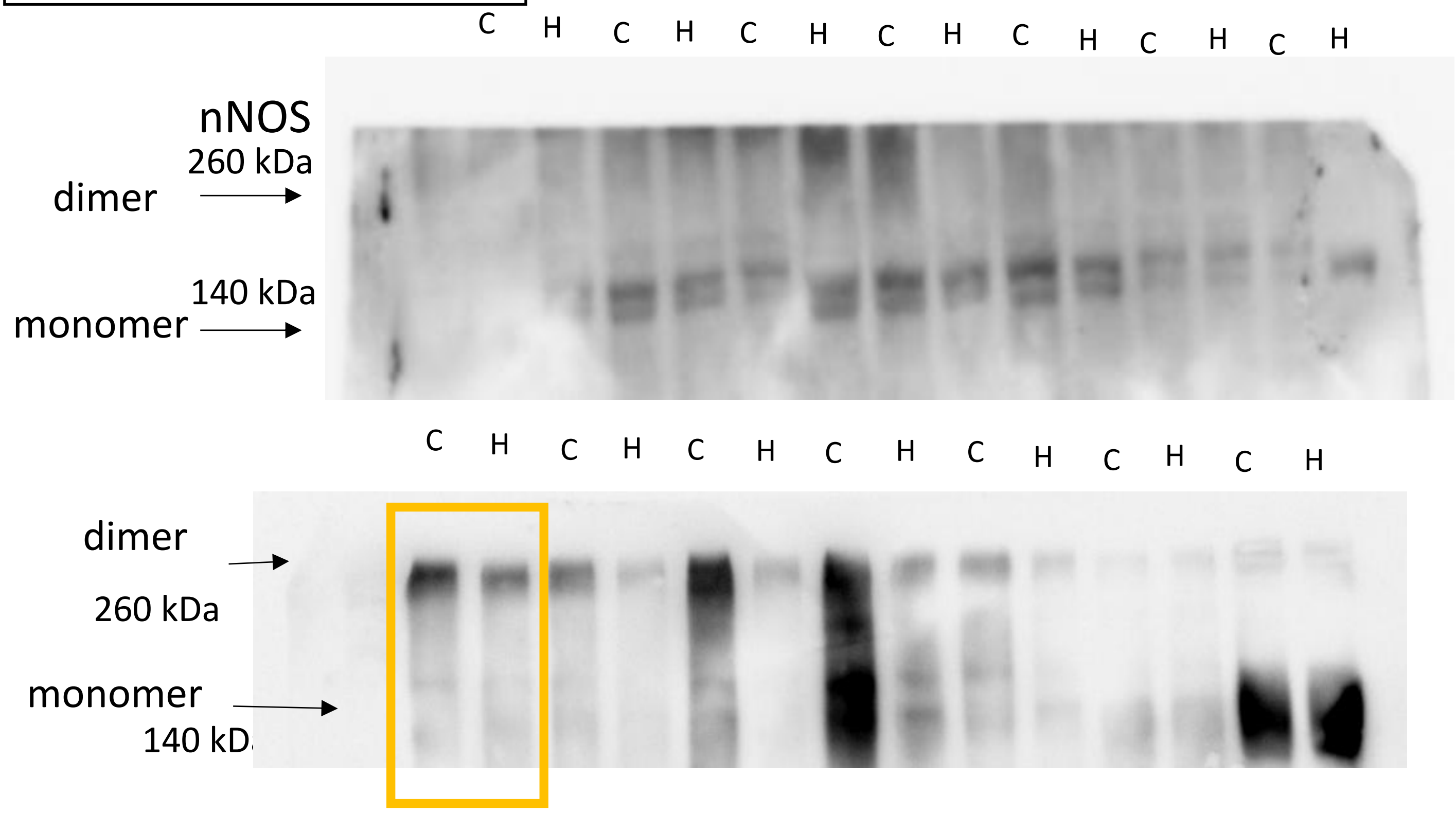

C = Control
H = Hipo

Ponceau staining was performed and is indicated in the corresponding experiments to confirm protein loading and transfer efficiency. However, images of the wells were not documented during this data acquisition; therefore, they are not available for inclusion.

Chemiluminescence for immunodetection detected with LAS 4000 equipment (GE HealthCare Life Science)

anti-nNOS 1:1000, sc-8309, Santa Cruz Biotechnology

Original Western blot Figure 6Aii

eNOS dimer / monomer

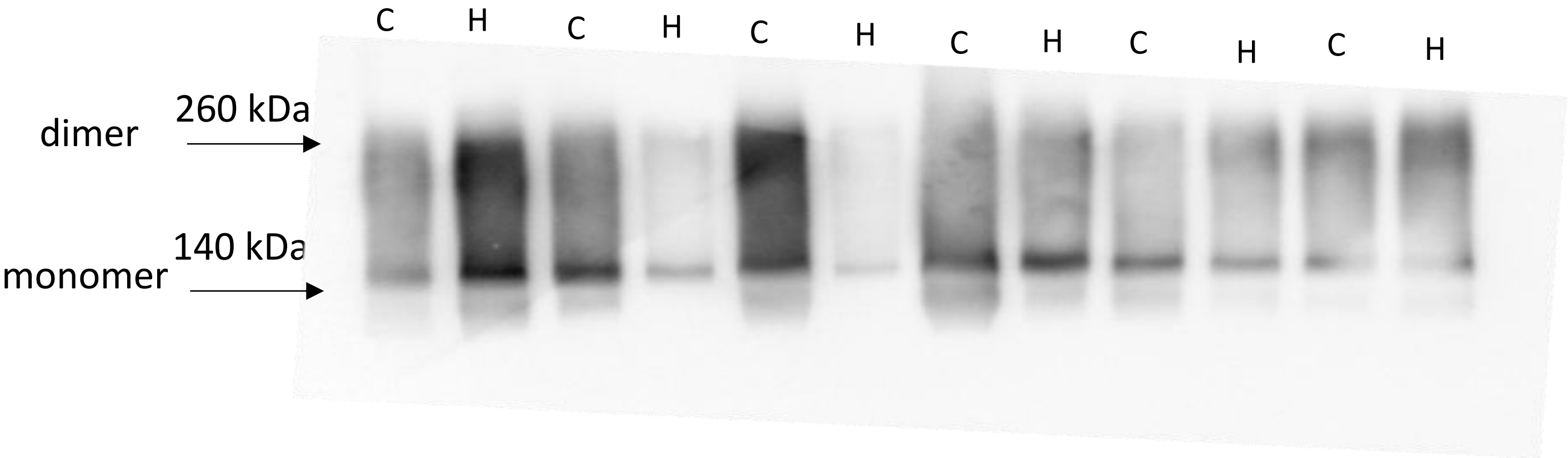

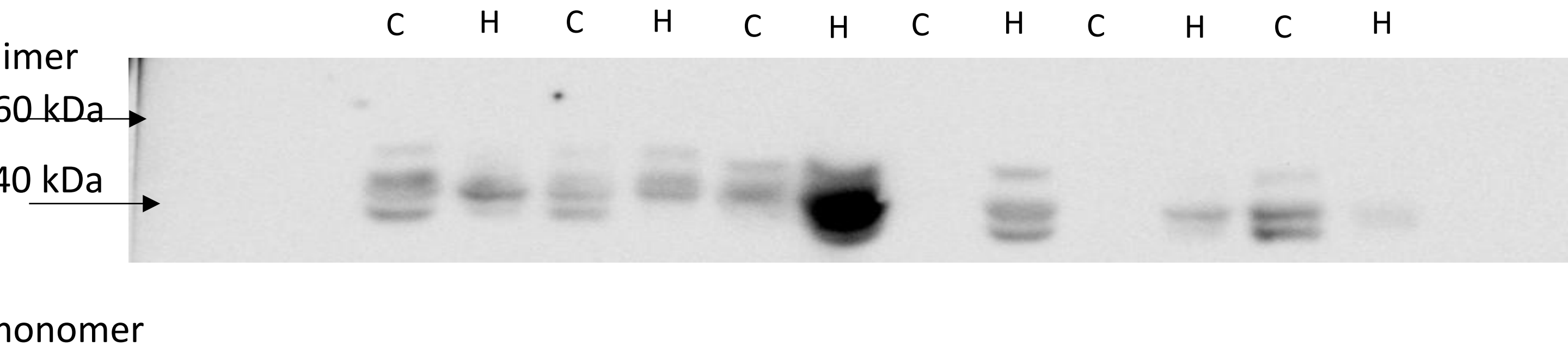

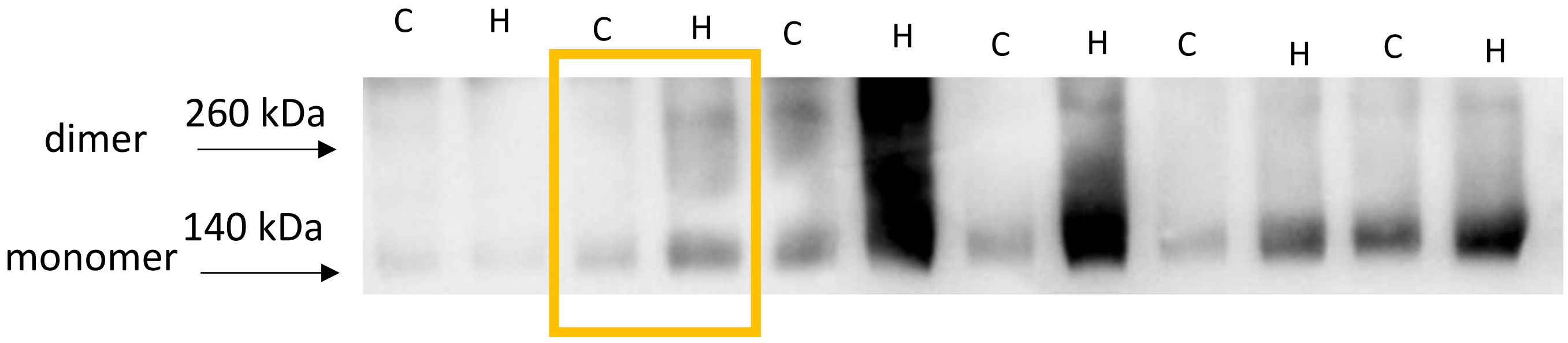

Ponceau staining was performed and is indicated in the corresponding experiments to confirm protein loading and transfer efficiency. However, images of the wells were not documented during this data acquisition; therefore, they are not available for inclusion.

Chemiluminescence for immunodetection detected with LAS 4000 equipment (GE HealthCare Life Science)

anti-nNOS 1:1000, sc-8309, Santa Cruz Biotechnology

Original Western blot Supplementary Figure 4F

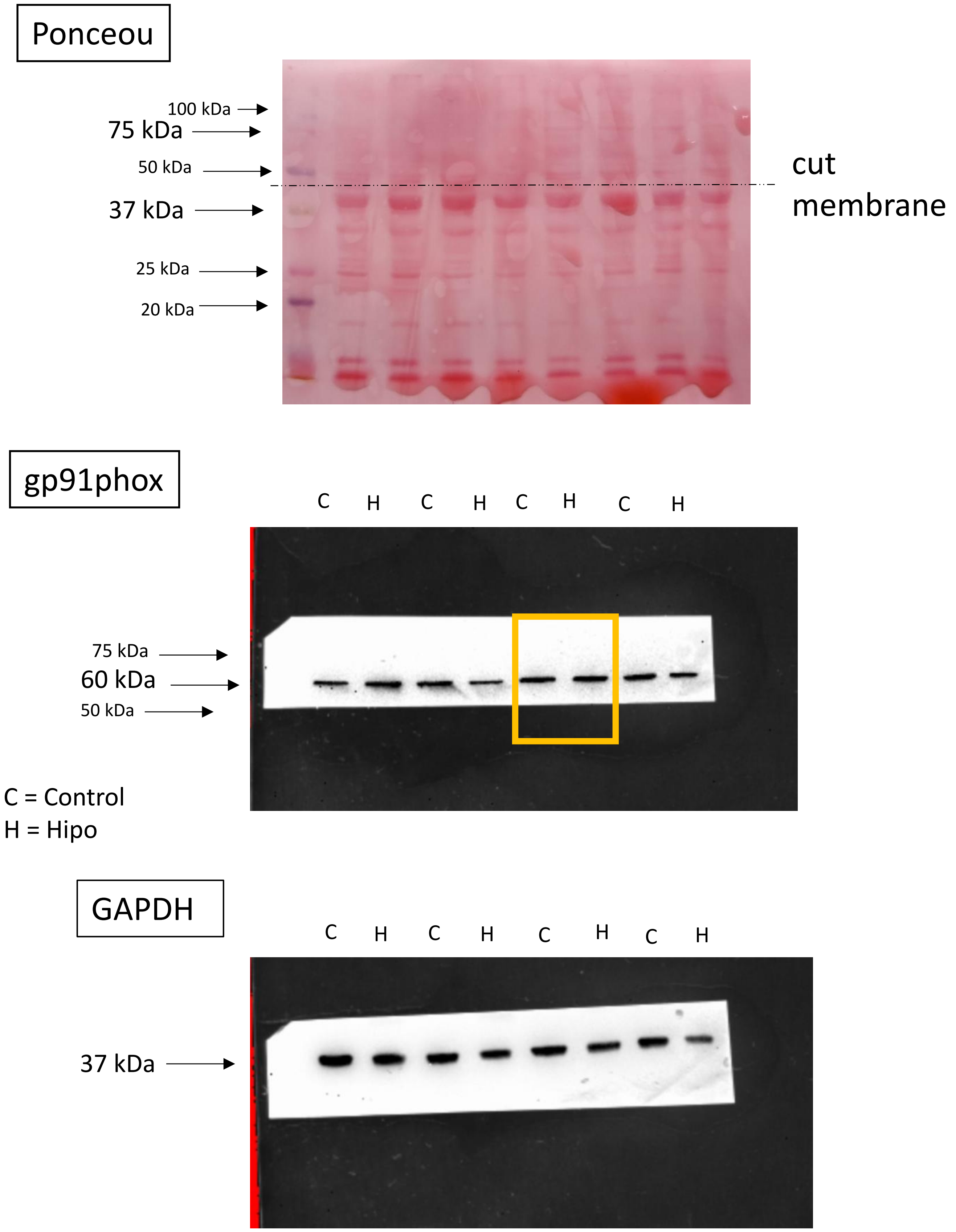

Chemiluminescence for immunodetection detected with iBrigt CL1000 (Thermo Fisher Scientific)

anti-GAPDH 1:1000, #32233, Santa Cruz Biotechnology;
anti-gp91phox 1:1000, #SAB5702937, Merck

Original Western blot Supplementary Figure 4F

Ponceou

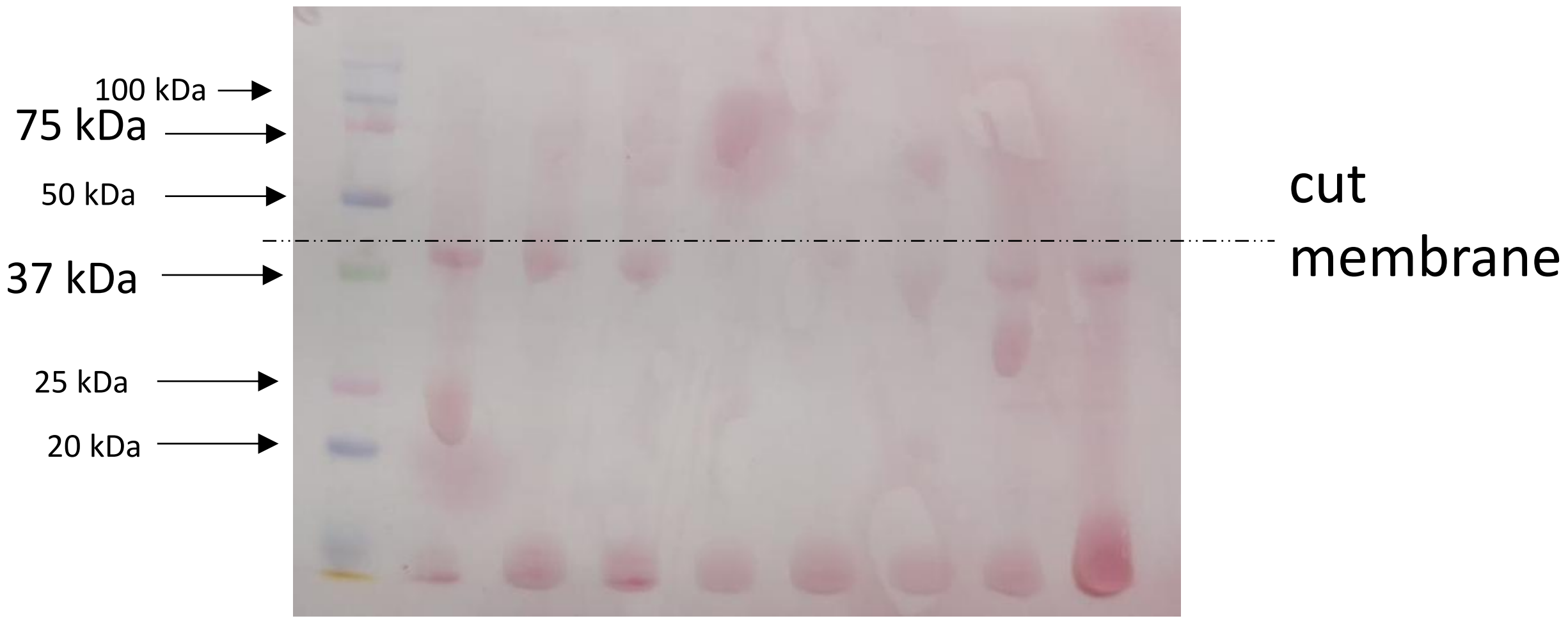

gp91phox

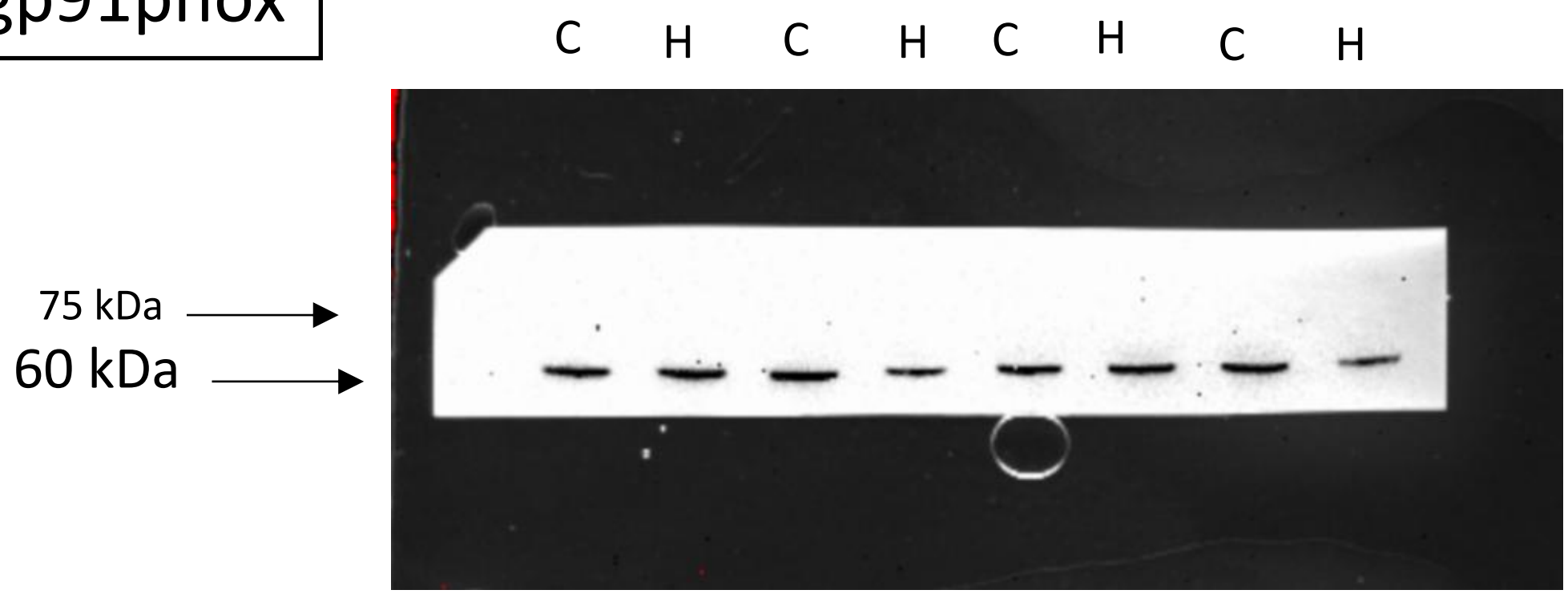

C = Control
H = Hipo

GAPDH

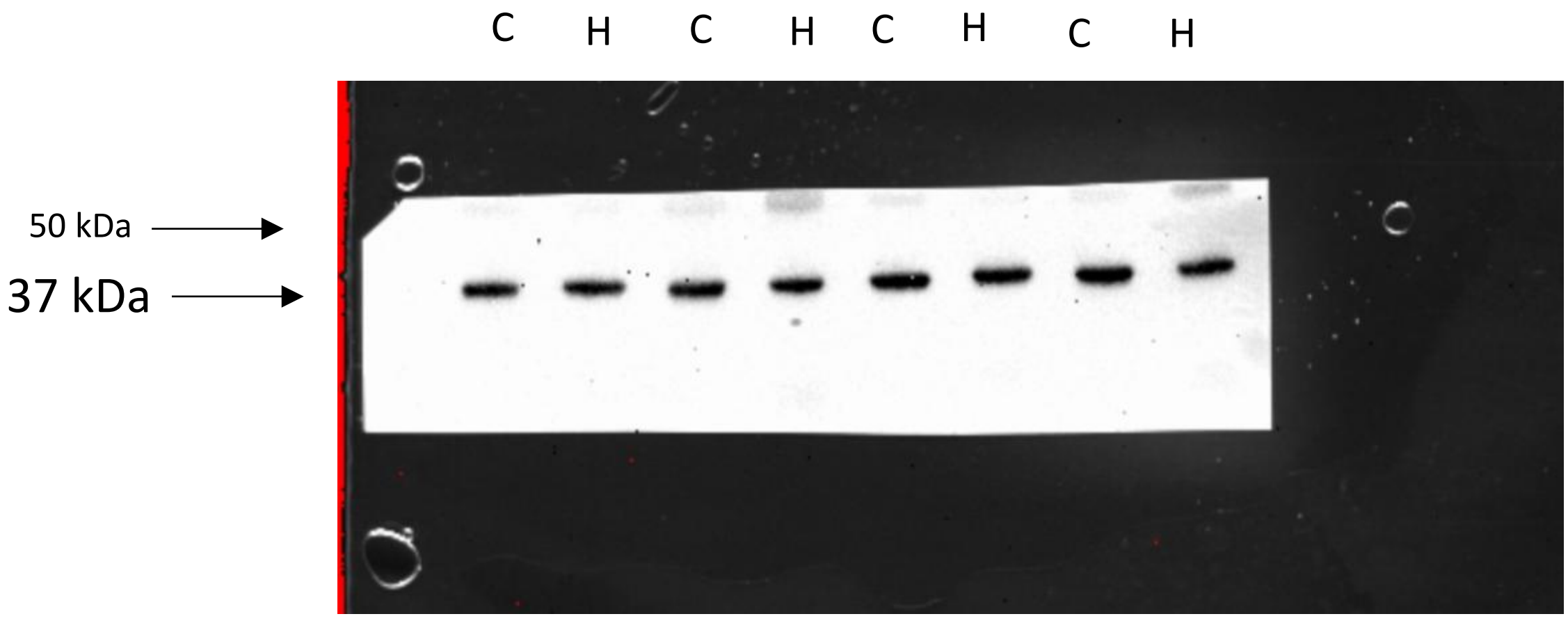

Chemiluminescence for immunodetection detected with iBrigt CL1000 (Thermo Fisher Scientific)

anti-GAPDH 1:1000, #32233, Santa Cruz Biotechnology;
anti-gp91phox 1:1000, #SAB5702937, Merck

Original Western blot Supplementary Figure 4E

Ponceou

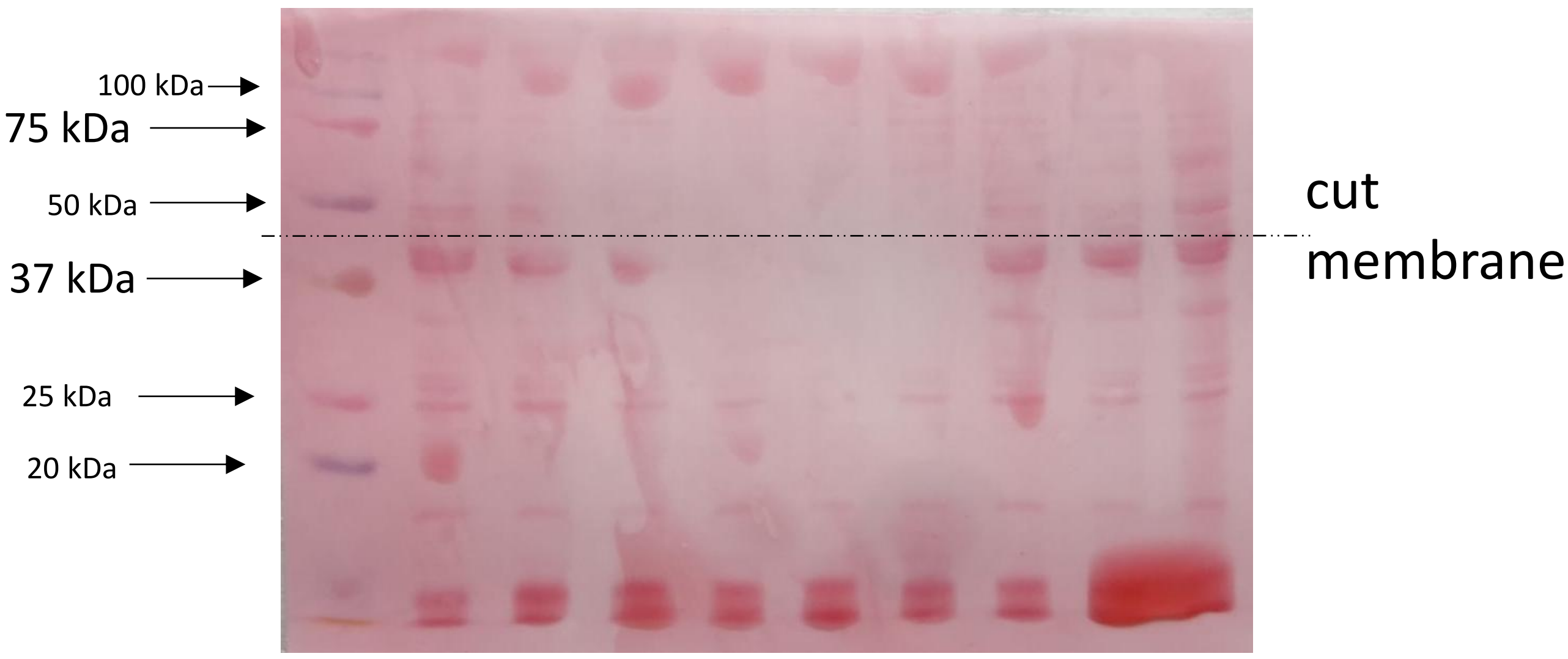

NOX4

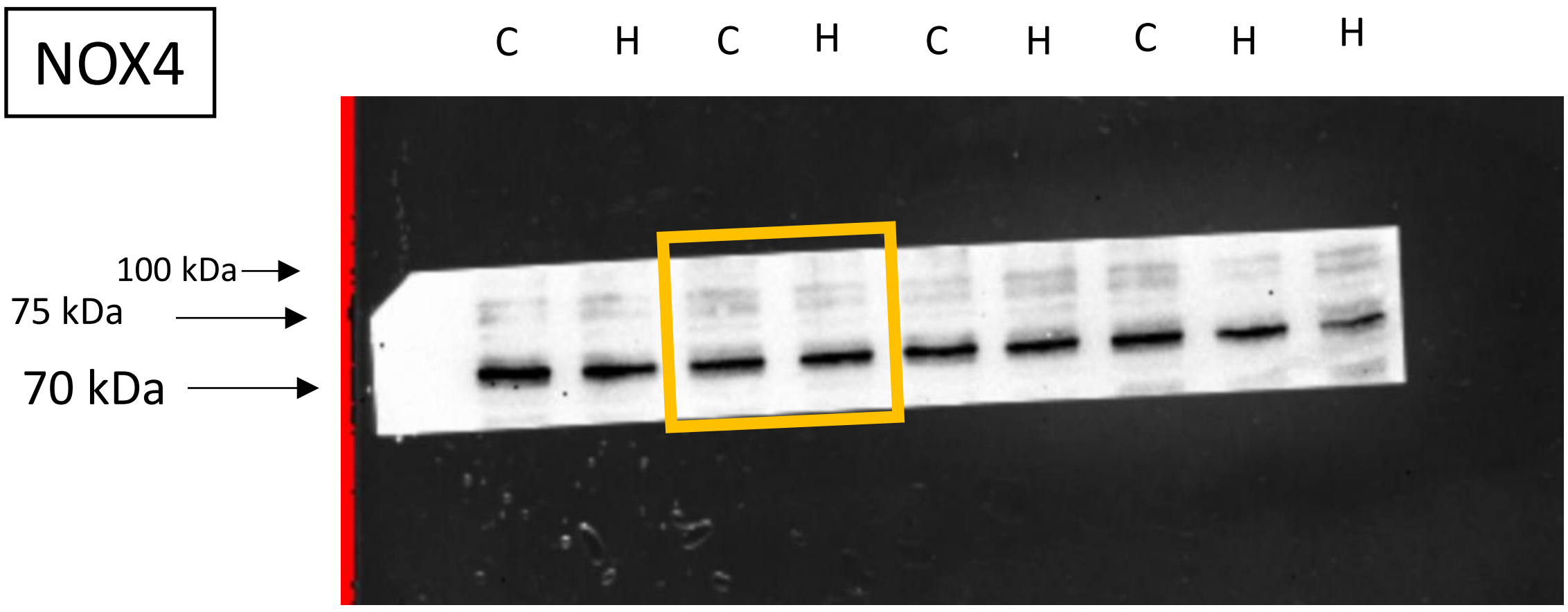

C = Control
H = Hipo

GAPDH

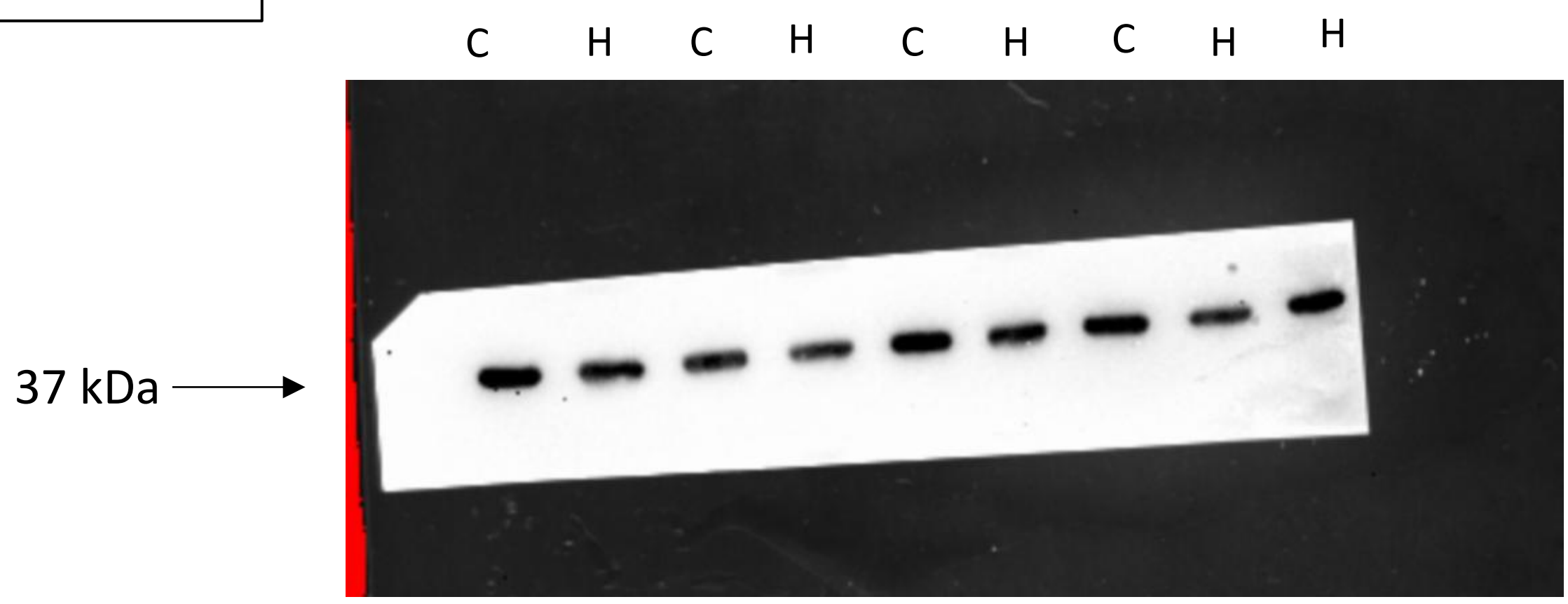

Chemiluminescence for immunodetection detected with iBrigt CL1000 (Thermo Fisher Scientific)

anti-GAPDH 1:1000, #32233, Santa Cruz Biotechnology;
anti-NOX4 1:1000, #MA5-32090 Invitrogen

Original Western blot Supplementary Figure 4E

Ponceou

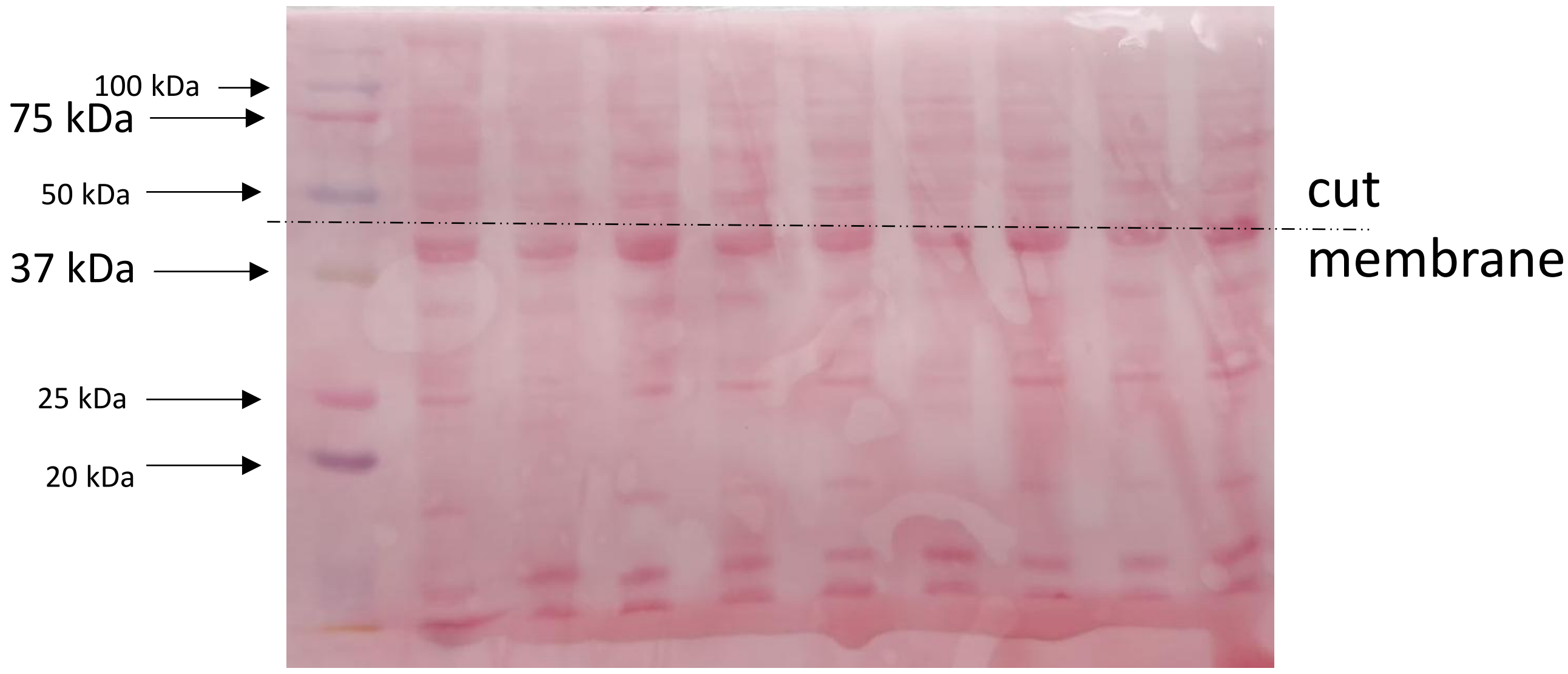

NOX4

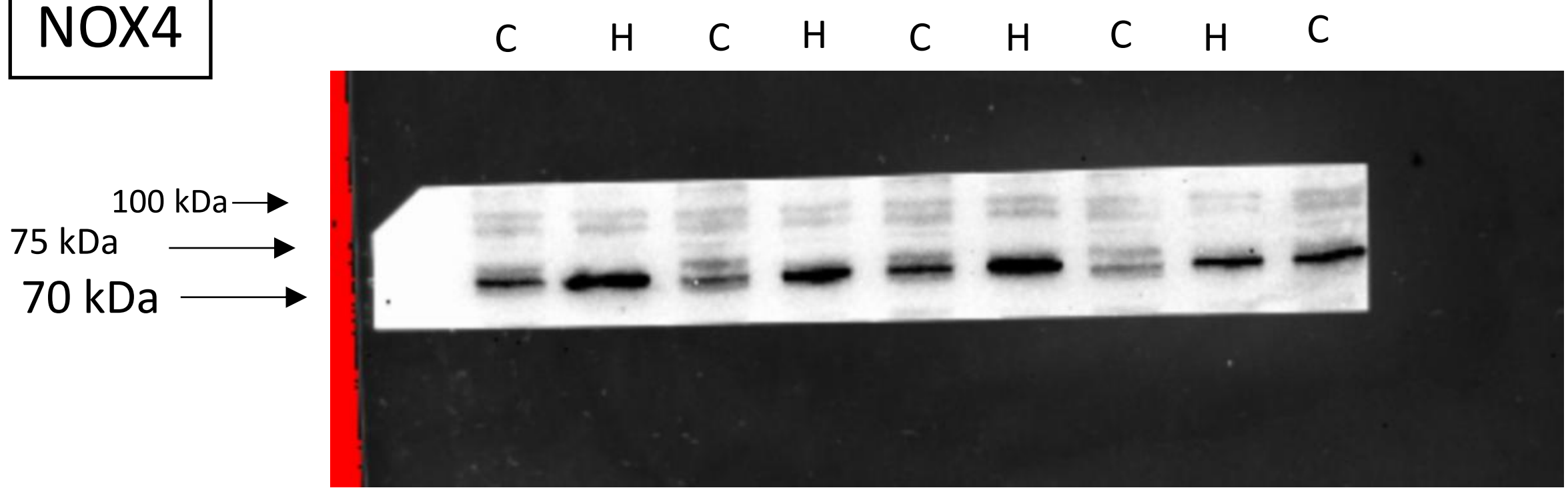

C = Control
H = Hipo

GAPDH

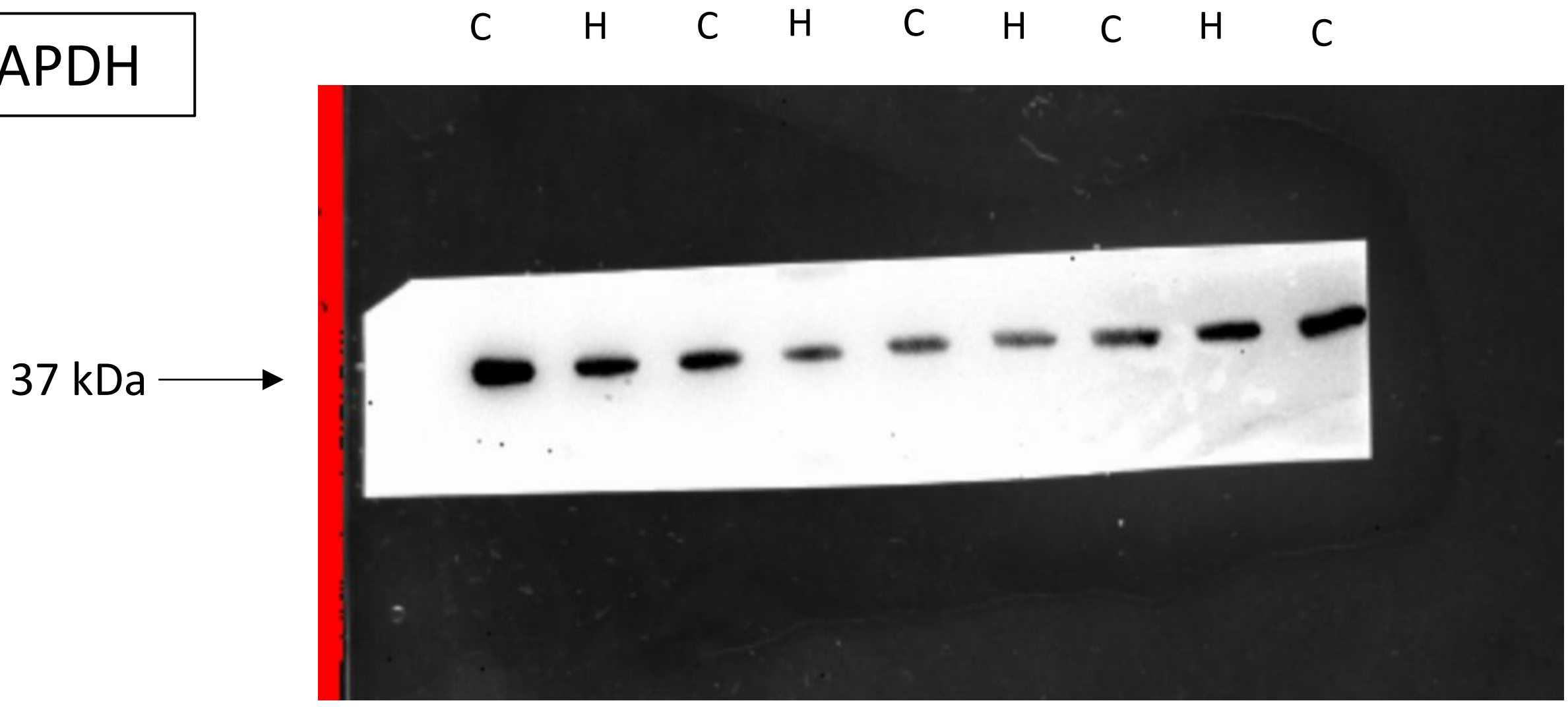

Chemiluminescence for immunodetection detected with iBrigt CL1000 (Thermo Fisher Scientific)

anti-GAPDH 1:1000, #32233, Santa Cruz Biotechnology; anti-NOX4 1:1000, #MA5-32090 Invitrogen

Ponceou

Original Western blot Supplementary Figure 4G

p47phox - Ponceau of representative western blot

50 kDa
37 kDa
25 kDa
20 kDa
15 kDa
10 kDa

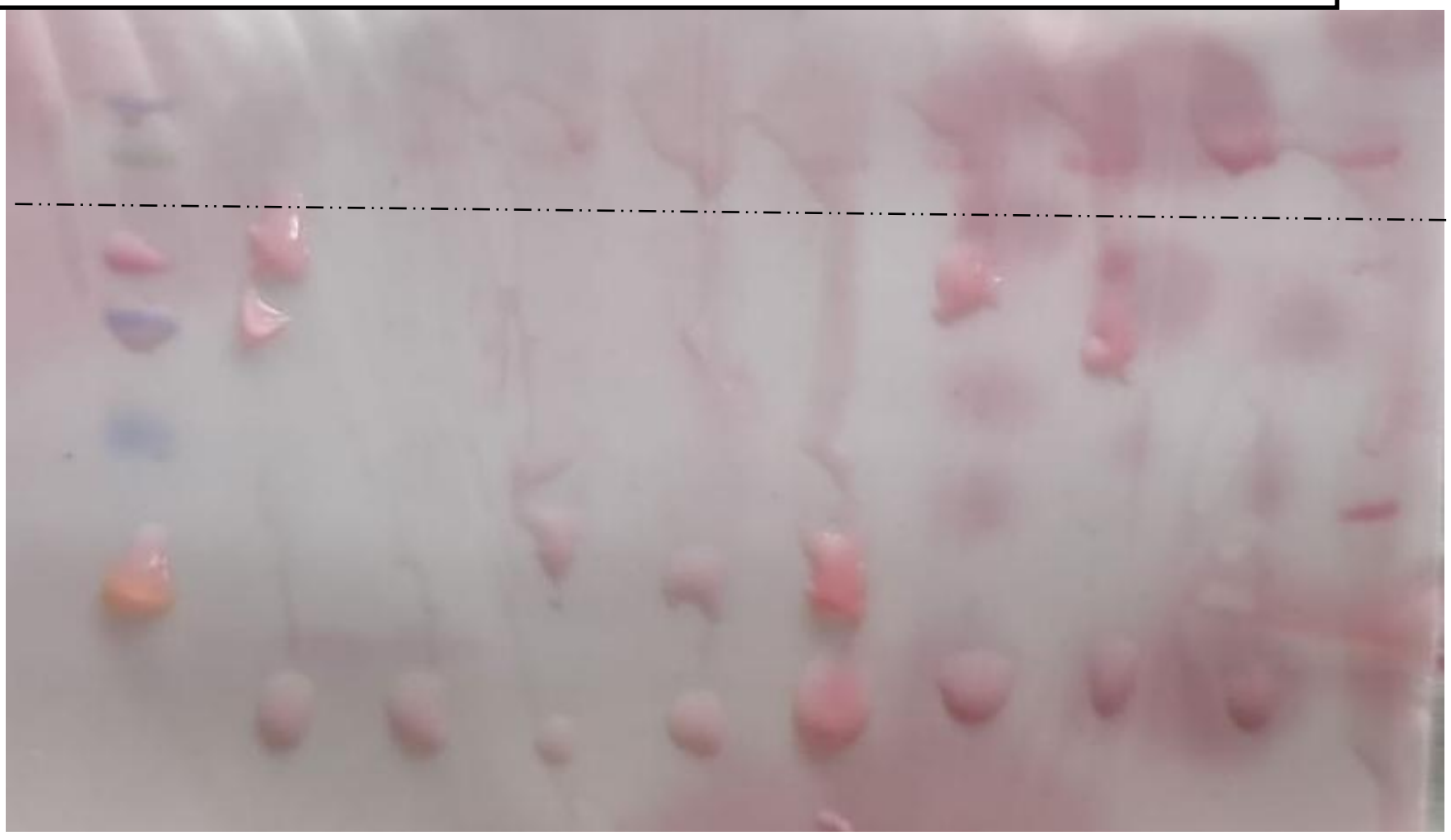

cut membrane

p22phox

C H C H C H C H H

25 kDa
20 kDa

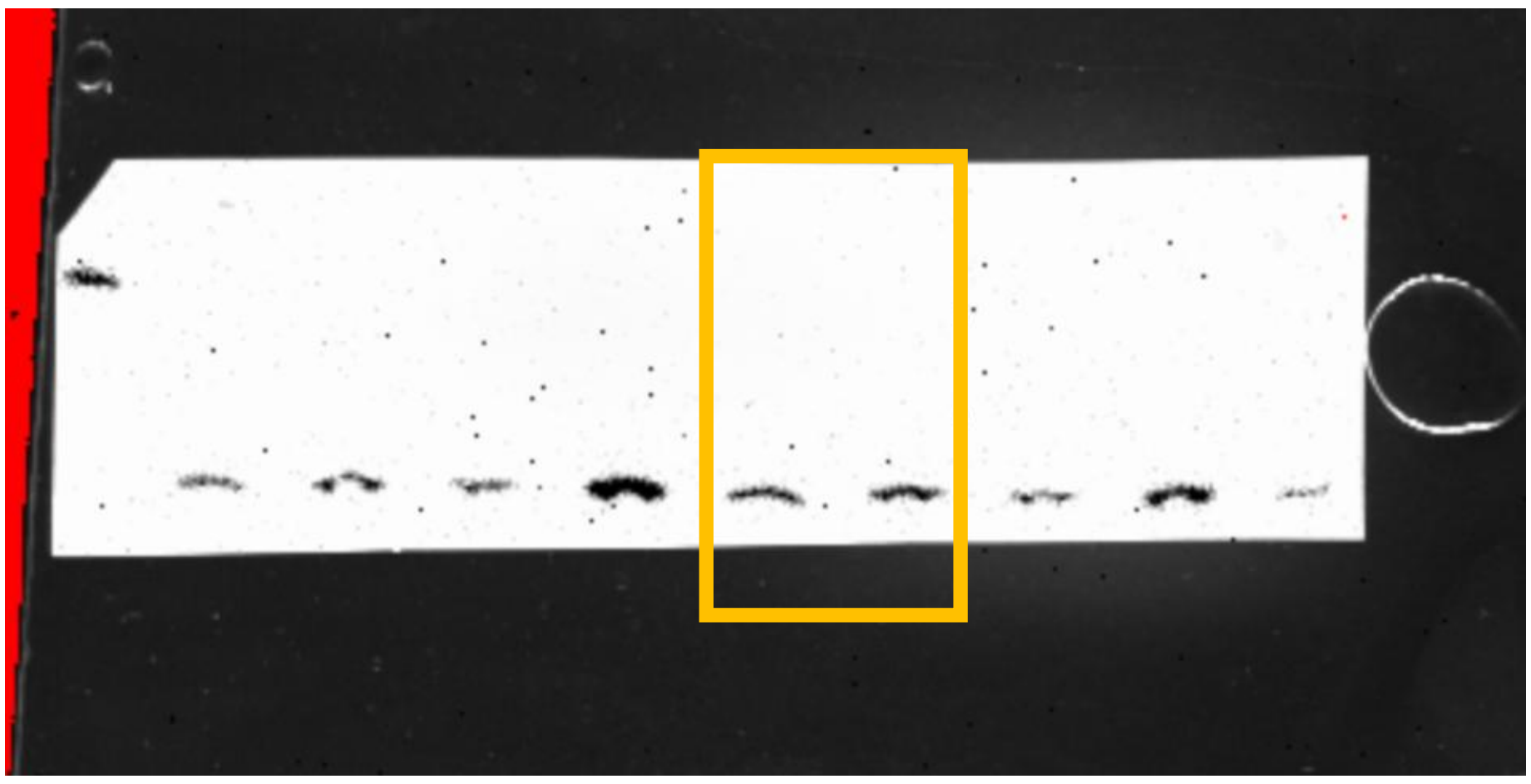

C = Control
H = Hipo

GAPDH

C H C H C H C H H

50 kDa
37 kDa

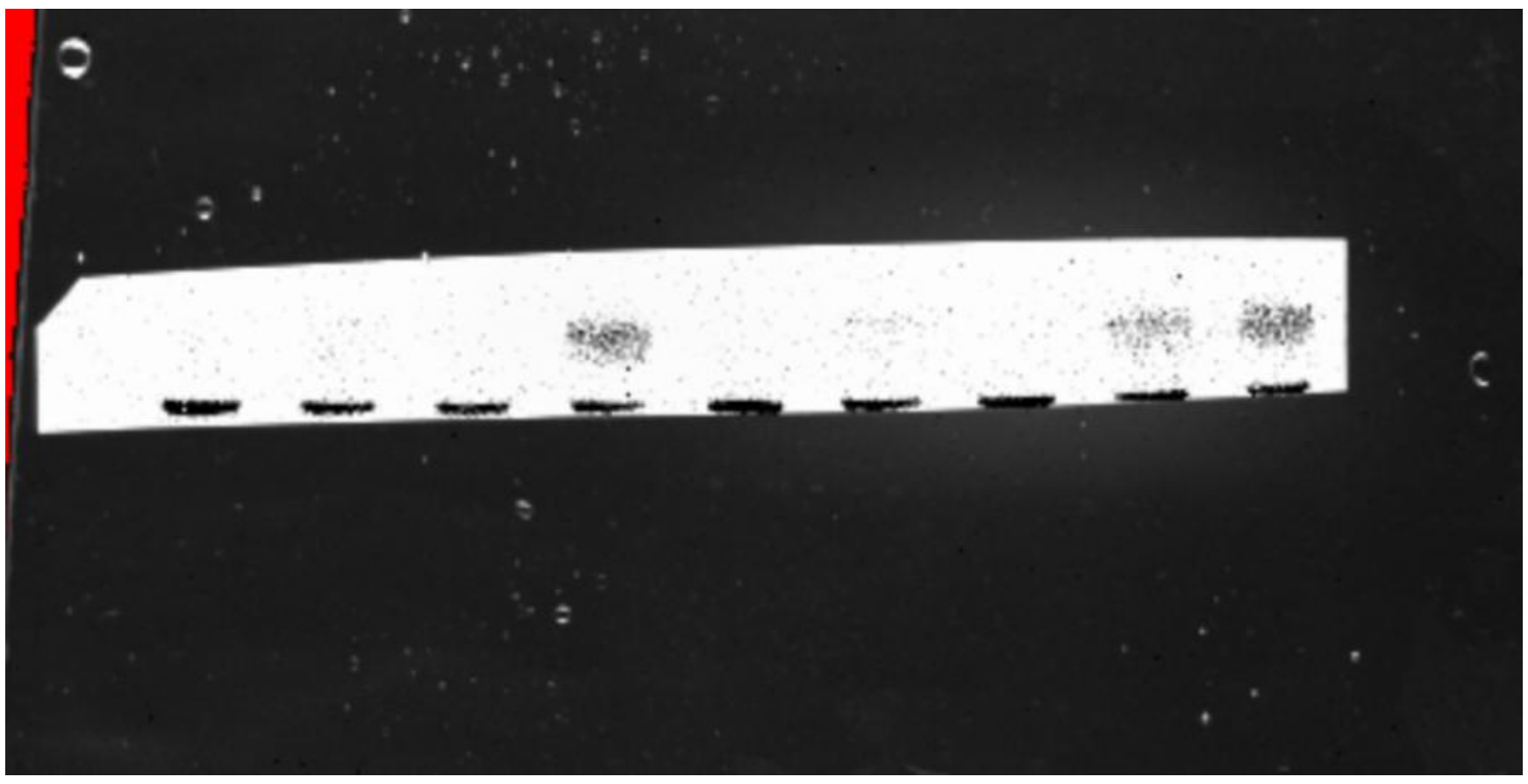

Chemiluminescence for immunodetection detected with iBrigt CL1000 (Thermo Fisher Scientific)

anti-p22phox 1:1000, #27297S, Cell Signaling;
anti-GAPDH 1:1000, #32233, Santa Cruz Biotechnology

Original Western blot Supplementary Figure 4G

Ponceou

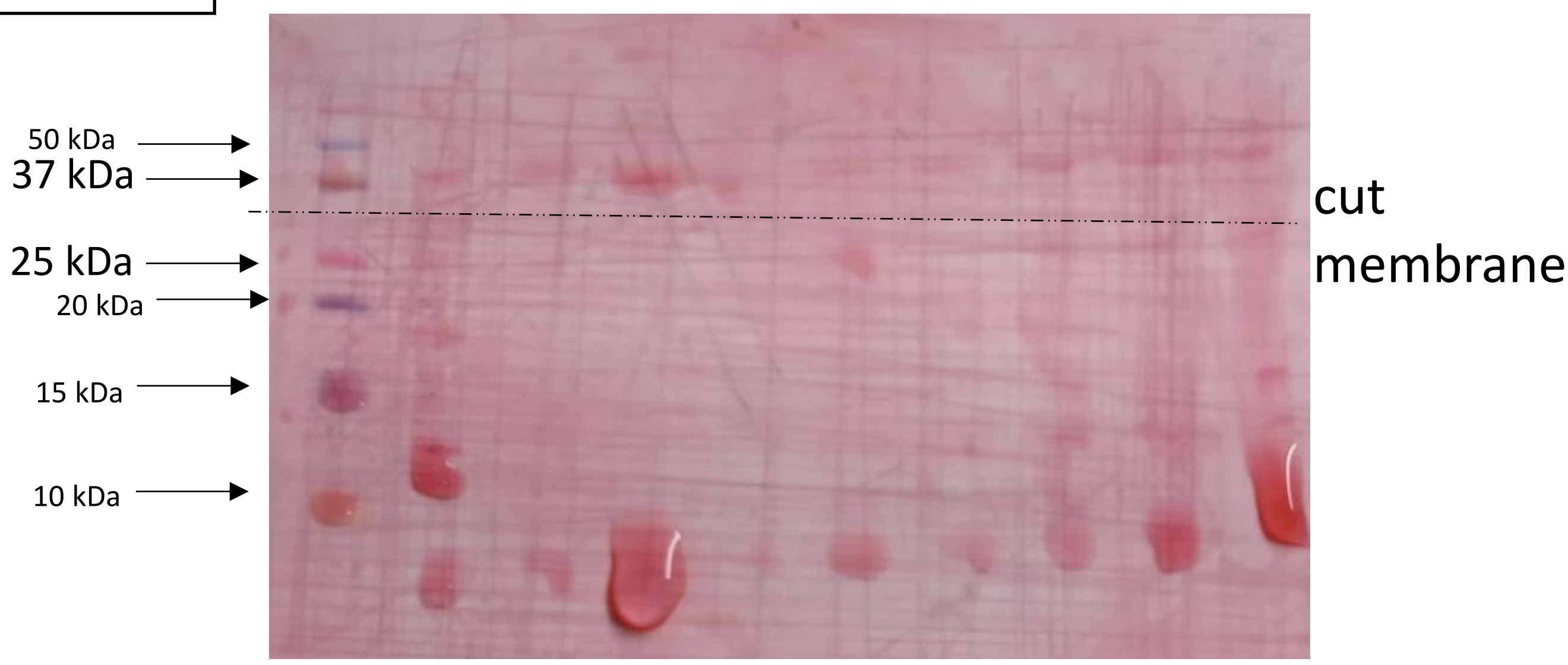

p22phox

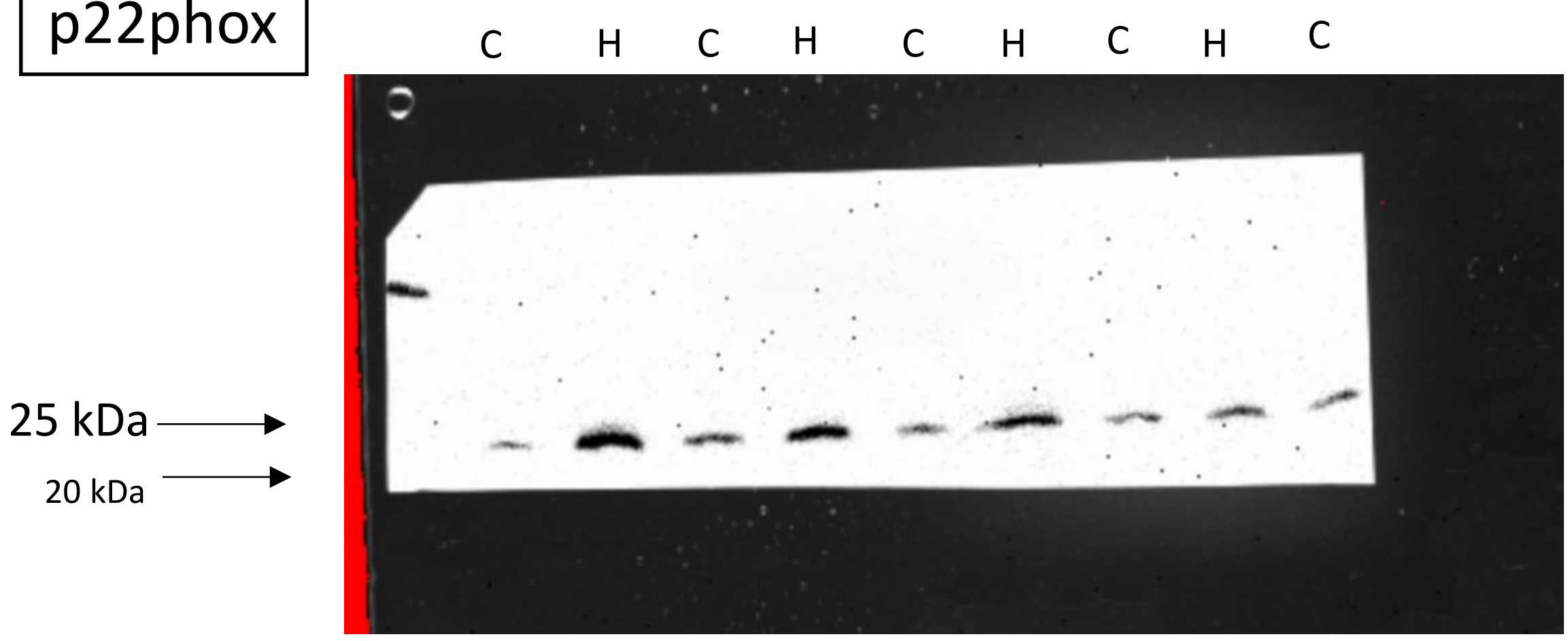

C = Control
H = Hipo

GAPDH

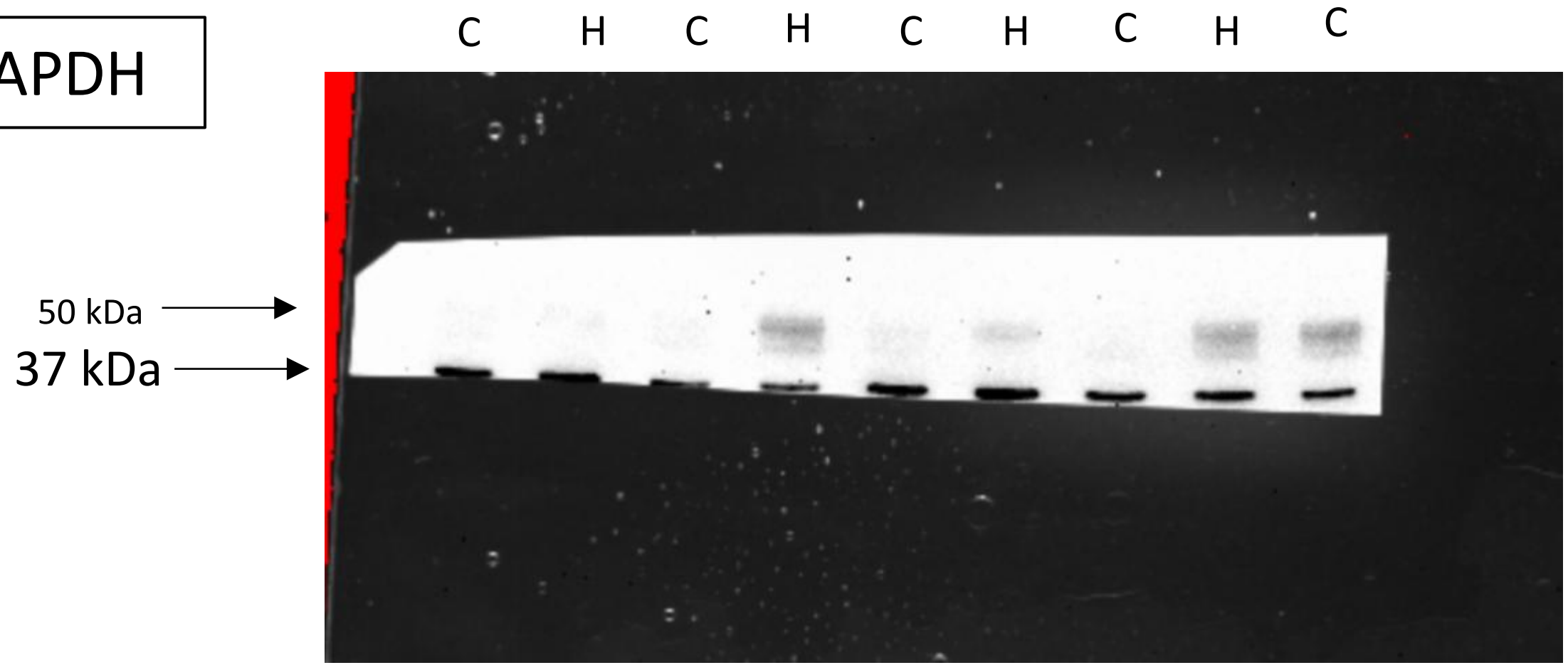

Chemiluminescence for immunodetection detected with iBrigt CL1000 (Thermo Fisher Scientific)

anti-p22phox 1:1000, #27297S, Cell Signaling;
anti-GAPDH 1:1000, #32233, Santa Cruz Biotechnology

Original Western blot Supplementary Figure 4H

Ponceou

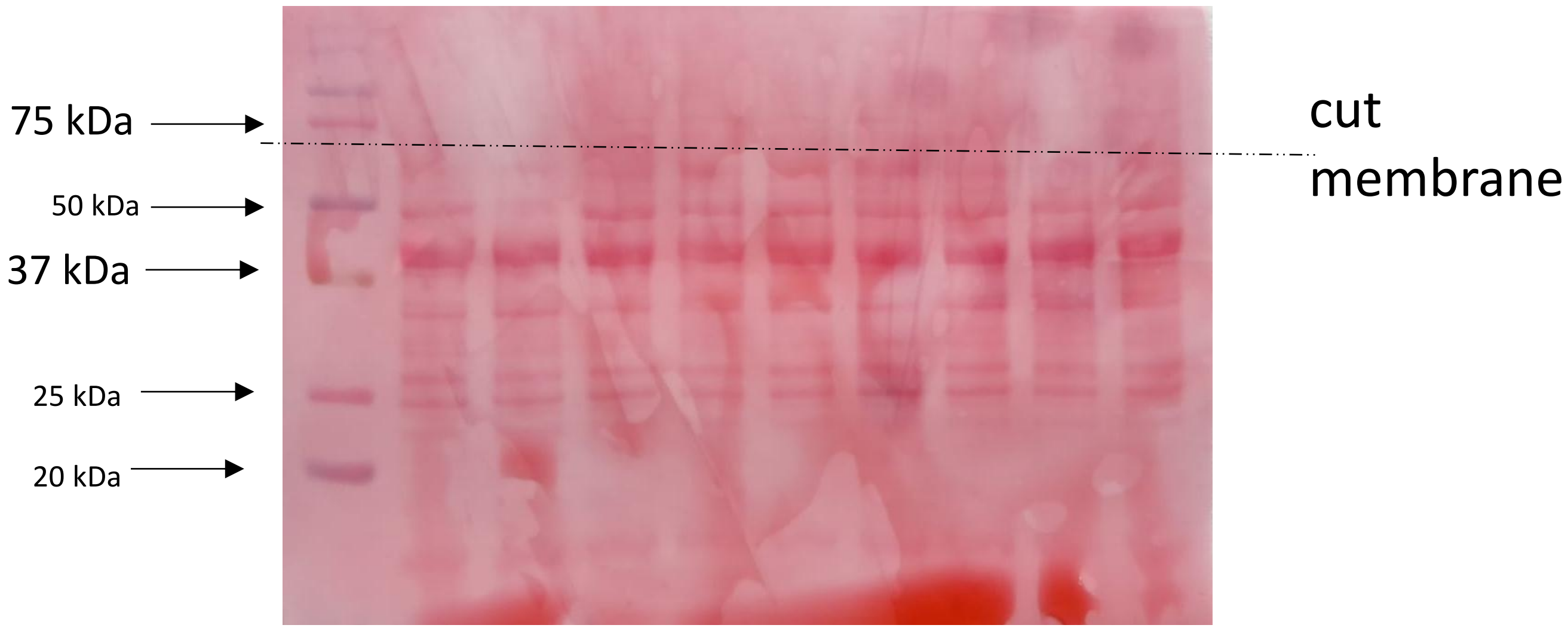

p47phox

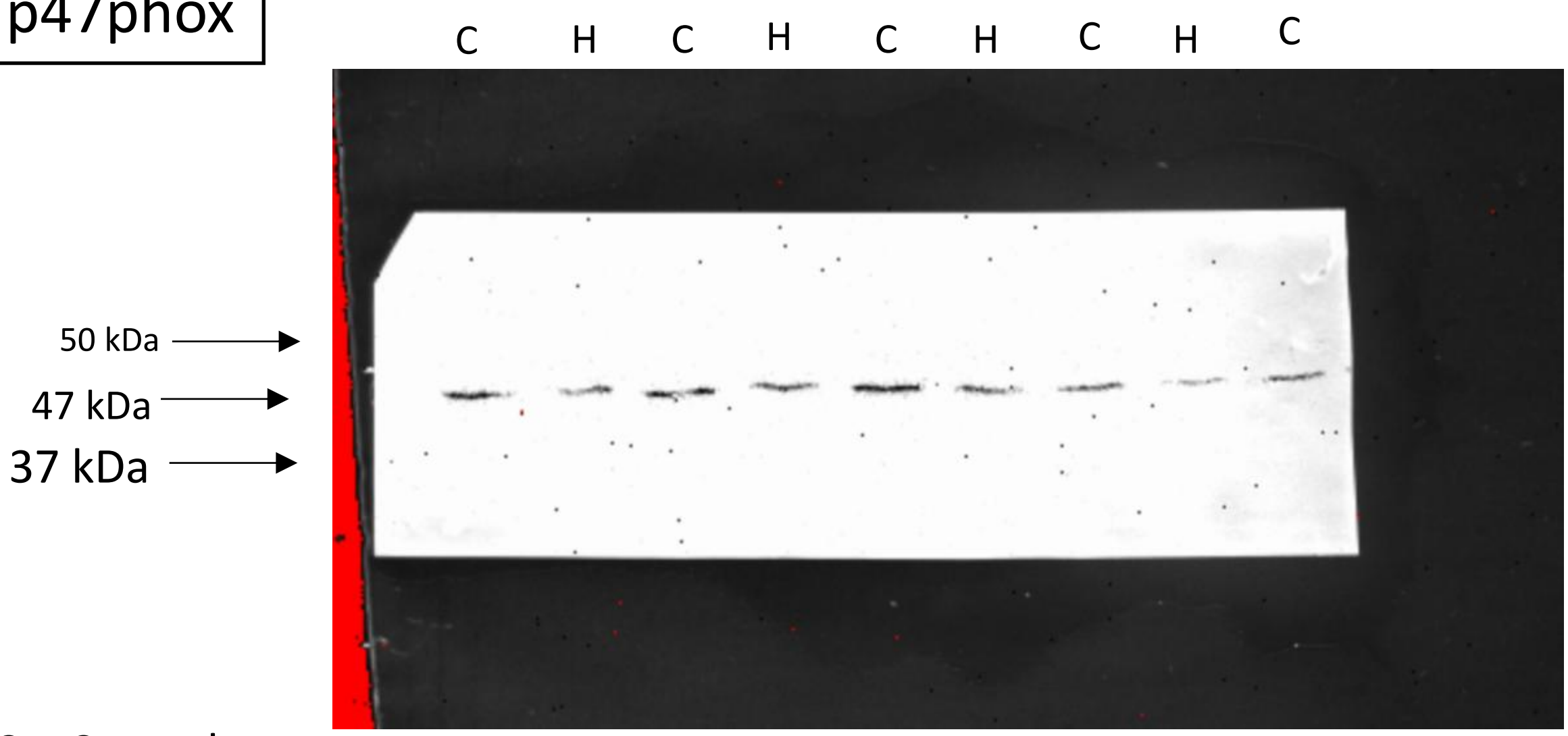

C = Control
H = Hipo

α-Tubulin

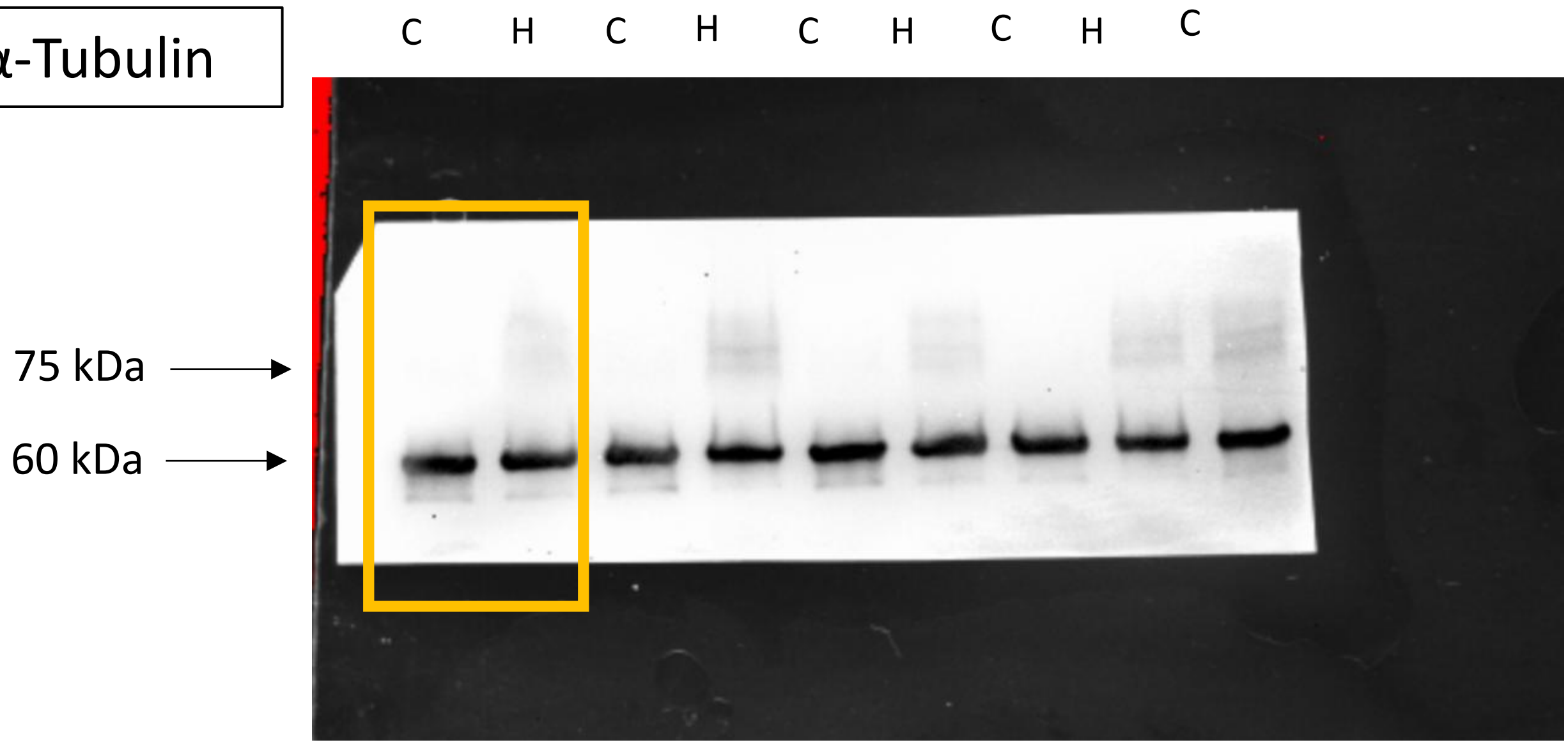

Chemiluminescence for immunodetection detected with iBrigt CL1000 (Thermo Fisher Scientific)

anti-p47phox 1:1000, #63290, Cell Signaling;
anti-GAPDH 1:1000, #32233, Santa Cruz Biotechnology

Original Western blot Supplementary Figure 4H

Ponceou

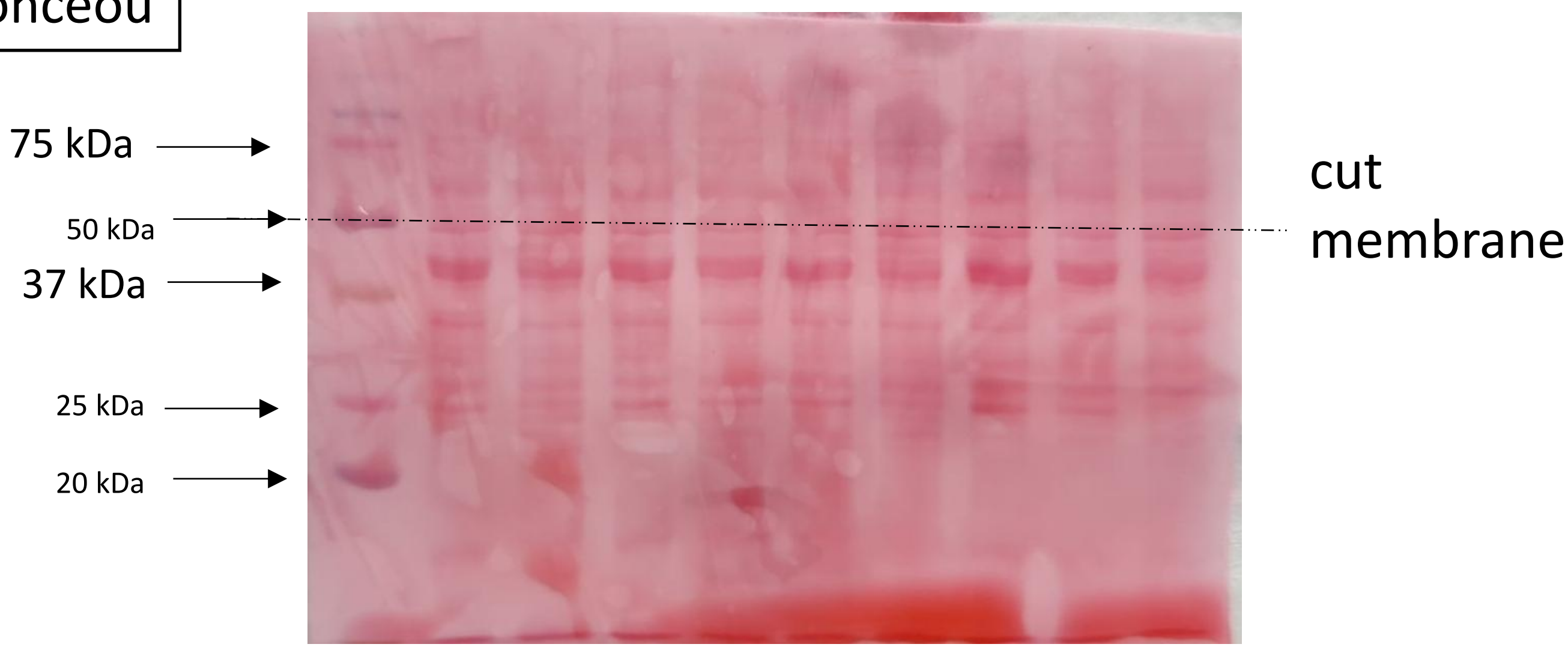

p47phox

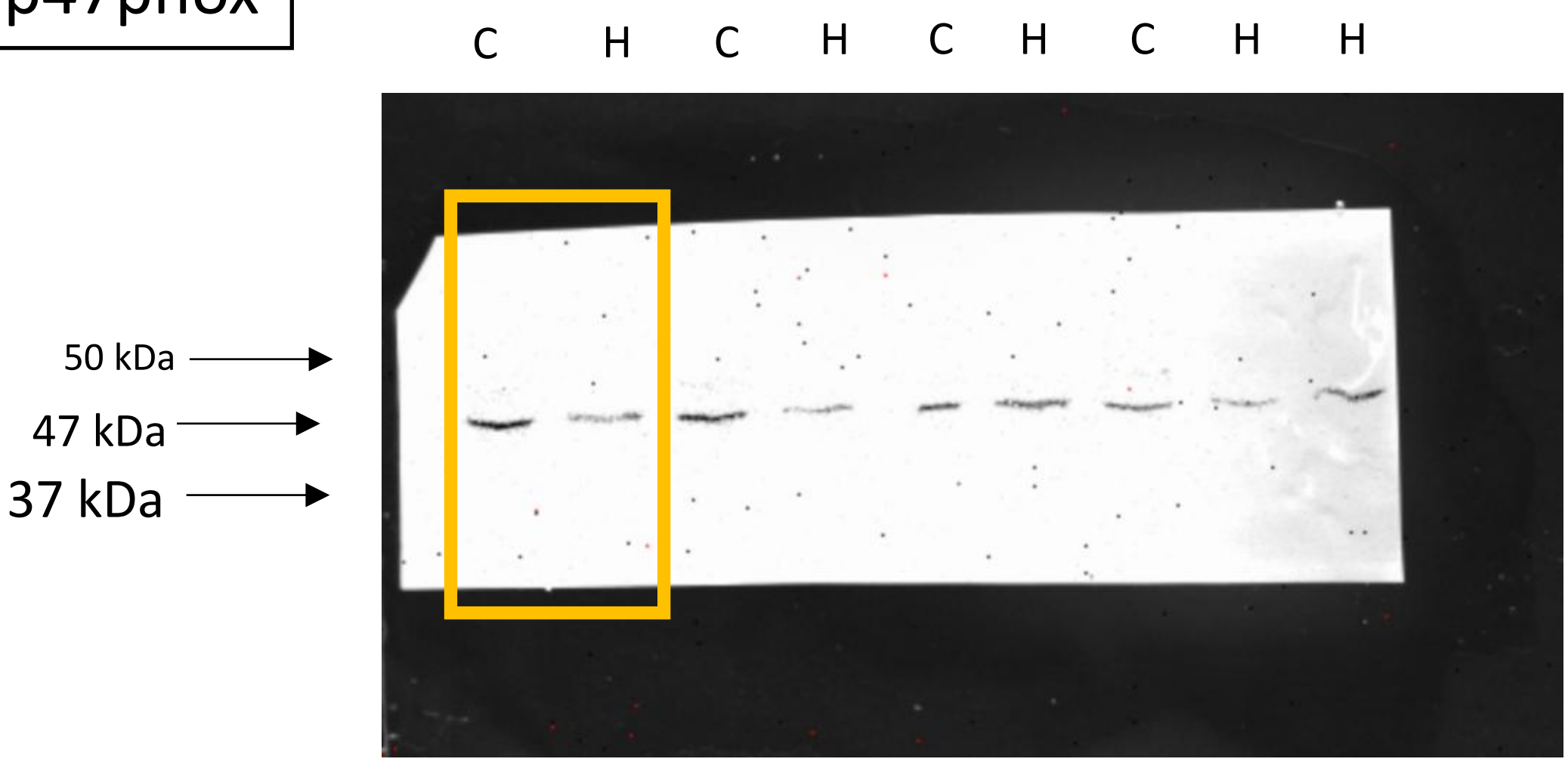

C = Control
H = Hipo

α-Tubulin

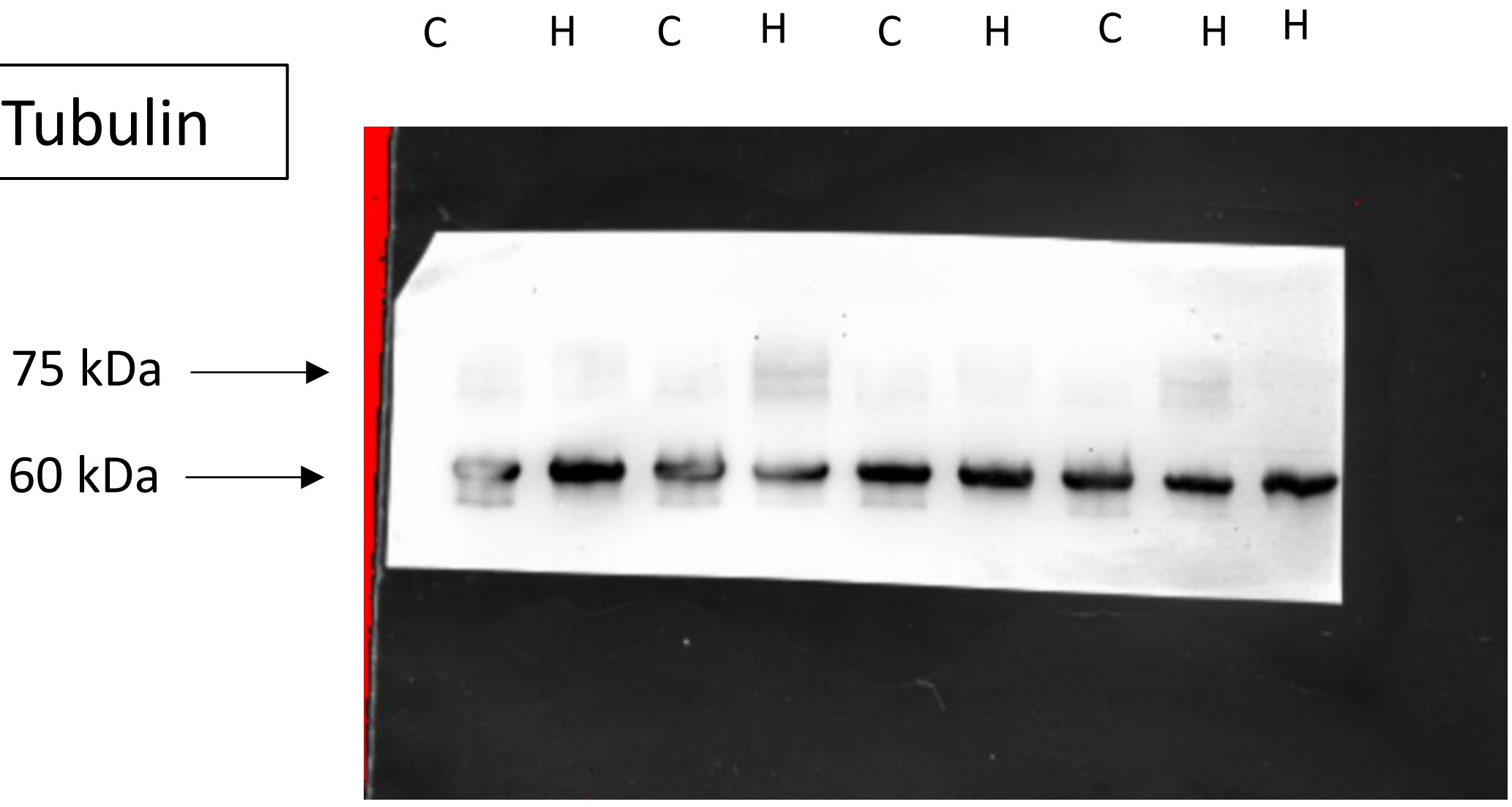

Chemiluminescence for immunodetection detected with iBrigt CL1000 (Thermo Fisher Scientific)

anti-p47phox 1:1000, #63290, Cell Signaling;
anti-GAPDH 1:1000, #32233, Santa Cruz Biotechnology
