# Supplementary material for: eNOS uncoupling drives oxidative stress and arrhythmogenic vulnerability in experimental hypothyroidism
Source: Redox Biol. 2026 Oct 20;98:104447. doi: 10.1016/j.redox.2026.104447 (PMC13720376; doi:10.1016/j.redox.2026.104447)
Supplement: Supplementary file 2 [file mmc1.docx]

**Supplementary Methods**

**eNOS uncoupling drives oxidative stress and arrhythmogenic vulnerability in experimental hypothyroidism**

* ^$^Diego Santos Souza^1,2^, *Leisiane Pereira Marques^2^, Aimée Obolari Durço^2^, Fabiana da Silva Alcântara^2^, Diego José Belato y Orts^2^, Melissa Tainan Silva Dias^3^, Michael Ramon de Lima Conceiçao^2^, Jorge Lucas Teixeira-Fonseca^2^, Luiz Fernando Santos Matos^1^, Maria Luiza Alarcon Mady Barbosa^1^, Daiara Custódio Santos^1^, Luciano dos Santos Aggum Capettini^3^, Carlos Roberto Prudencio^4^, Danilo Lustrino^1^, Jader Santos Cruz^5^, ^#^Danilo Roman-Campos^2^.

1. Department of Physiology, Federal University of Sergipe, São Cristóvão, Brazil.
2. Department of Biophysics, Federal University of São Paulo, São Paulo, Brazil.
3. Department of Pharmacology, Federal University of Minas Gerais, Belo Horizonte, Brazil.
4. Research Scientist, Immunology Center, Institute Adolfo Lutz. São Paulo, Brazil.
5. Department of Biochemistry and Immunology, Federal University of Minas Gerais, Belo Horizonte, Brazil.

*The authors contributed equally to this work

^#^Corresponding Author: Department of Biophysics, Federal University of Sao Paulo, Botucatu Street, 862, Biological Science Building, Sao Paulo, SP, Brazil.

^$^Co-corresponding Author: Department of Physiology, Federal University of Sergipe. Av. Marechal Rondon, S/N, Rosa Elze, Aracaju, SE, Brazil

E-mail addresses: drcampos@unifesp.br (D. Roman-Campos), santos.dss@outlook.com (D.S. Souza).

**Methods**

**Animals**

The Swiss mice (25 - 30 g) were obtained from the Animal Care Facility of the Federal University of São Paulo (Ethics Committee for Animal Research - UNIFESP #9389091220). All procedures involving mice were conducted in accordance with the guidelines of the National Council for the Control of Animal Experimentation (CONCEA), Brazil, and NIH Guide for the Care and Use of Laboratory Animals. Hypothyroidism was induced adding 0.1% (w/vol) of methimazole to their drinking water for 21 days ^1,2^. All the experimental procedures were realized in the 22 days; the mice were anesthetized with inhalation of isoflurane induced at 5% and maintained at 2% with 60% O_2_ ^3^. For euthanasia, animals were deeply anesthetized with 5% isoflurane, followed by decapitation to allow subsequent heart collection.

For the analysis of total triiodothyronine (T3), free thyroxine (T4), and thyroid-stimulating hormone (TSH), immunoassay tests from Labtest Scientific (Lagoa Santa, MG, Brazil) were used, according to the manufacturer’s instructions. Serum samples were randomly selected from the animals, with two to three samples per cage, in order to verify the successful induction of hypothyroidism.

***In vivo* electrocardiography recordings**

Mice were anesthetized with isoflurane 2%. Electrocardiograph (ECG) recordings were performed using a non-invasive ECG (ECG-PC version 2.07, Brazilian Electronic Technology—TEB, Belo Horizonte, MG, Brazil) and arrhythmias susceptibility in vivo was evaluate after injection of caffeine (120mg/kg, i.p.) and dobutamine (1mg/kg,i.p.) ^1^. To investigate the role of ROS on arrhythmia susceptibility, the animals were pretreated with N-Acetyl-L-cysteine (NAC-100 mg/kg, i.p.) or tetrahydrobiopterin (BH4, 20 mg/kg, i.p.- Sigma-Aldrich Brazil) 30 minutes prior to ECG acquisition and exposure to the dobutamine–caffeine arrhythmia protocol . This 30-minute pretreatment window was selected based on established in vivo protocols to ensure sufficient systemic distribution and effective modulation of the cardiac redox environment before the onset of adrenergic stress^4^. Analysis of the ECG, LabChart version 8 was used. The parameters, PR interval, complex QRS heart rate (BPM) and QT interval were evaluated. QT interval was normalized for rodents (QTcn-B = QT/√(RR/*f*)). Where “*f*” is the mean of duration heart beat in control (RR = 127ms) taken as previously described ^5^.

**Cardiomyocyte isolation**

Heparin (1000 UI/Kg, i.p.) was administrad, after 15 minutes the mice were euthanized and their hearts removed. The aorta was then cannulated and promptly set up in a Langendorff to the cardiomyocyte isolation. Experiments were realized using an enzymatic assay as described by Shioya (2007) ^6^. In this process, hearts were initially perfused with calcium-free solution (Cell isolation buffer - CIB) containing (in mM): 130 NaCl, 5.4 KCl, 0.5 MgCl_2_.6H_2_O, 0.33 NaH_2_PO_4_, 22 glucose, 25 HEPES, and 0.4 EGTA (pH 7.4), maintained at 37°C for 3 minutes to clean the heart. After this process, the solution was replaced with CIB containing 0.3 mM CaCl_2_, type II collagenase (1 mg/mL), type XIV protease (0.06 mg/mL), and trypsin (0.09 mg/mL). After 15 minutes of perfusion, the heart was removed from the cannula, and the atria and ventricles were separated. The endocardial left ventricles were cut into small pieces and subjected to an additional 15 minutes of digestion (37°C) with CIB supplemented with collagenase 1mg/ml, trypsin 0.06 mg/ml, protease 0.06 mg/ml, and 0.7 mM CaCl_2_. After these two enzymatic digestion steps, cells were isolated from the tissue by gentle agitation using a transfer pipette for 3min. This solution, containing the isolated cells, was filtered, centrifuged at 1000 rpm for 30 s, resuspended, and maintained in CIB solution containing 1.2 mM CaCl_2_ and bovine serum albumin (BSA, 2 mg/mL), for 15 minutes at 37°C. Finally, the cells were centrifuged again (1000 rpm for 30 s) and maintained in Tyrode solution with the following composition (in mM): 140 NaCl, 5.4 KCl, 0.5 MgCl_2_.6H_2_O, 0.33 NaH_2_PO_4_, 11 glucose, 5 HEPES, and 1.8 CaCl_2_.2H_2_O (pH 7.4), and used for a maximum of 4 hours after dissociation.

**Patch-Clamp Recordings**

Patch-clamp recordings in both voltage-clamp and current-clamp modes were performed on isolated ventricular endocardial cardiomyocytes using an EPC-10 patch-clamp amplifier (HEKA, Holliston, Massachusetts, USA) and a patch-clamp AxoPatch 200B (Axon Instruments, Molecular Devices, USA) at room temperature (20–26°C). Recording micropipettes had a resistance of 1.5 to 3 MΩ. After achieving a whole-cell configuration, a 2-minute waiting period was observed to allow for the establishment of ionic equilibrium between the pipette solution and the intracellular environment. Cells with series resistance greater than 8 MΩ were excluded from the analysis. Data analysis was conducted using Clampfit 11.1 software (Molecular Devices, Sunnyvale, CA, USA).

**Action Potential Recordings**

Action potentials (APs) were recorded in the whole-cell current-clamp configuration. APs were evoked by a 1 nA square pulse of 3-5 ms duration at a frequency of 5 Hz. The internal pipette solution contained (in mM): 130 L-aspartate, 130 KOH, 20 KCl, 10 HEPES, 2 MgCl_2_.6H_2_O, 5 NaCl, with pH adjusted to 7.2 with KOH. The external solution was Tyrode solution as previously mentioned. The average of 60 action potentials was evaluated for each cell. This protocol was repeated in the absence and presence of KN-93 . To induce arrhythmia *in vitro*, cardiomyocytes were subject to a stimulus of 5 Hz (100 pulses) and then a pause of 10 s to observe spontaneous events ^7^. The analysis of arrhythmias was performed on a cell-by-cell basis, applying a score for proarrhythmic events after AP stabilization, as follows: Delay afterdepolarization (DAD) was scored as 1; trigger was scored as 2.

**Macroscopic Ionic Current Recordings**

All currents were recorded in the whole-cell voltage-clamp configuration.

**Total Potassium Currents (I_K_)**

Voltage-dependent potassium currents, specifically the inwardly rectifying potassium current (I_K1_), transient outward (I_to_), delayed rectifier (I_Kdr_), and steady-state (I_ss_) currents, were determined. The internal pipette solution contained (in mM): 140 KCl, 1 MgCl₂.6H_2_O, 11 EGTA, and 10 HEPES, pH adjusted to 7.2 with KOH. The external solution contained (in mM): 140 NMDG, 5.6 KCl, 1.8 CaCl₂.2H_2_O, 0.1 CdCl₂.H_2_O, 0.5 MgCl₂.6H_2_O, 11 glucose, and 10 HEPES, pH adjusted to 7.4 with HCl. The holding potential of -80 mV was used followed by a series of depolarizing voltage steps ranging from -14 0 mV to +60 mV (in 10 mV increments) were applied for 3000 ms. To assess steady-state inactivation, a standard double-pulse protocol was used, consisting of conditioning pulses ranging from -140 mV to +60 mV for 3000 ms, followed by a constant test step to +60 mV to elicit the remaining available current. The recovery from inactivation was analyzed using a conditioning pulse to 0 mV for 1000 ms, followed by a recovery interval (-80 mV) ranging from 10 to 3000 ms, then a test pulse (0 mV) to assess the fraction of recovered current. Current values to I_Kdr_ were determined at the peak (after 500 ms) and at the end of the 3000 ms to I_SS_ depolarization pulse. The normalized conductance (Steady-State Activation/Inactivation)-voltage (G-V) curves were fitted with the Boltzmann equation. Recovery from inactivation was examined using a triple-pulse protocol and fitted with a double-exponential function ^8,9^. All potassium currents were sampled at 20kHz rate.

**Sodium Current (I_Na_)**

Macroscopic I_Na_ were recorded with the following internal solution (in mM): 125 CsCl, 35 aspartic acid, 30 TEA-Cl, 5 Mg-ATP, 10 EGTA, 11 HEPES, 0.5 MgCl₂ and 3.6 phosphocreatine (dissodium salt – 7.2 mM Na^+^), with pH adjusted to 7.3 with CsOH. The external solution contained (in mM): 125 TEA-Cl, 50 NaCl, 1.8 CaCl₂, 5 CsCl, 1.2 MgCl₂, 2 MnCl_2_, 20 HEPES, 11 glucose and 4-aminopyridine with pH adjusted to 7.3 with CsOH. To isolate I_Na_, 1 µM isradipine and 10 µM nifedipine were added to the external solution. To evaluate Na+ current activation, depolarizing steps from -80 mV to +40 mV (1 mV increments) for 50 ms were applied from a holding potential of −100 mV. Steady-state inactivation was assessed using a conditioning pre-pulse (from -100 to 0 mV, 5 mV increments) for 2 s, followed by a fixed test pulse to -20 mV to elicit remaining current. Current values were converted to conductance using Ohm's law. The normalized conductance (Steady-State Activation/Inactivation) curves were fitted with the Boltzmann equation. Recovery from inactivation was assessed using a double-pulse protocol consisting of two depolarizing pulses (P1 and P2) to −20 mV with fixed durations of 50 ms and 100 ms, respectively, separated by a variable interpulse interval ranging from 0.1 to 1000 ms ^10^. Recovery from inactivation was fitted with a biexponential function with free time constants The recordings were low-pass filtered (cutoff frequency 2 kHz) and digitized at 20 kHz.

**Calcium Current (I_Ca_)**

For I_Ca_ , the internal solution was (in mM): 120 CsCl, 20 TEA-Cl, 3 Na-ATP, 10 HEPES, 10 EGTA, and 2 Mg-ATP (pH 7.2 with CsOH). The external solution used was (in mM): 150 TEA-Cl, 0.5 MgCl_2_⋅6H_2_O, 1.8 CaCl₂2.H_2_O, 10 HEPES, and 11 glucose (pH 7.4 with TEA-OH). To evaluate Ca^2+^ current activation, depolarizing steps from -80 mV to +60 mV (10 mV increments) during 300 ms were applied. Steady-state inactivation was assessed using a conditioning pre-pulse (from -80 to +0 mV) during 2 s, followed by a fixed test pulse to +10 mV to elicit remaining current. To evaluate recovery from inactivation, a 300 ms conditioning pulse to 0 mV was followed by a recovery interval (-80 mV) ranging from 10 to 900 ms. Then, a test pulse (0 mV) was applied to assess the fraction of recovered current. The normalized conductance (Steady-State Activation/Inactivation) curves and inactivation curves were fitted with the Boltzmann equation. Recovery from inactivation was examined using a double-pulse protocol and fitted with a single exponential function ^11^. I_Ca_ was sampled at 20kHz.

**Sodium-Calcium Exchanger Current (I_NCX_)**

I_NCX_ was recorded using a ramp protocol and measured as Ni^2+^ sensitive current. Cells were held at a potential of -80 mV before being stepped to +60 mV for 50 ms. Following this, a ramp protocol was applied to -120 mV at a rate of 0.09 V/s. Recordings were sampled at 10 kHz. To isolate the I_NCX_, the internal pipette solution contained (in mM): 20 NaCl, 1.19 MgCl₂, 10 HEPES, 20 TEA-Cl, 33.8 CaCl₂, 40 EGTA, 40 aspartic acid, with pH adjusted to 7.2 with TEA-OH. The external solution was composed of (in mM): 145 NaOH, 145 aspartic acid, 5 HEPES, 2 MgCl₂, 1 CaCl₂, 2 BaCl₂, with pH adjusted to 7.3 with NaOH. Ouabain (50 µM) was added to the bath solution to block the Na^+^/K^+^ pump, and nicardipine (2 µM) was used to block the L-type calcium current (I_Ca,L_). I_NCX_ was determined as the current sensitive to the perfusion of 5 mM Ni^2+^, and was measured both before (Ni^2+^(-)) and after (Ni^2+^ (+)) application ^12^.

**Calcium transient and spontaneous Ca²⁺ event recordings in ventricular cardiomyocytes**

Isolated ventricular cardiomyocytes were loaded with 5 μM Fluo-4 AM in Tyrode’s solution for 30 minutes at room temperature. After incubation, cells were rinsed and kept in fresh Tyrode’s solution during imaging. Intracellular Ca²⁺ transients, Ca²⁺ sparks, and out-of-pacing calcium events were recorded under field stimulation at 5 Hz for 30 seconds to standardize excitation frequency. Fluorescence signals were acquired using a confocal laser-scanning microscope (LSM 880, Carl Zeiss, Germany) equipped with a 63× oil-immersion objective. Fluo-4 was excited using a 488 nm argon laser, and emission was collected at wavelengths >510 nm. The amplitude of Ca²⁺ transients were analyzed using Clampfit 11 software (Molecular Devices, USA). Quantification of Ca²⁺ sparks was performed with ImageJ (NIH, USA) using the SparkMaster plug-in, which allows automated and reproducible detection of localized calcium release events in confocal line-scan images ^1,13,14^.

**Measurements of reactive oxygen species (ROS) generation**

Cardiomyocytes ROS was evaluated using dihydroethidium 10µM (DHE Invitrogen, MA, USA), incubeted by 30 min, Mitotracker green 300nM (Invitrogen), incubeted by 15 min, MitoSOX red 5µM (Invitrogen). When appropriate, some cardiomyocytes were pre-incubated for 30 min with tetrahydrobiopterin (BH4,100 μM - Sigma-Aldrich Brazil) an NOS cofactor; hydrogen peroxide (H_2_O_2_,1 mM - Sigma-Aldrich Brazil); 15 min with	apocynin (10μM - Sigma-Aldrich Brazil) a NADPH oxidase inhibitor; 15 min with	N(G)-Nitro-L-arginine methyl ester (L-NAME, 100 μM - Sigma-Aldrich Brazil) general NOS inhibitor; 15 min with	L-N(5)-(1-iminoethyl)-ornithine ( L-NIO - 1uM - Cayman Chemical , MI, USA), specific inhibitor for eNOS; 15 min with	Nω-Propyl-L-arginine hydrochloride (NPLH,100 nM - Cayman Chemical, MI, USA), specific inhibitor for nNOS; 15 min with 1400W dihydrochloride (100nM - Cayman Chemical, MI, USA), specific inhibitor for iNOS ^15–17^. For all analyses we used Image J 1.52 × software (NIH, Bethesda, MD, USA). In all imaging experiments, at least one animal from each group was used on the same day to avoid artifacts from the imaging acquisition system.

**Nitric oxide and nitrite levels**

Nitric oxide (NO) production was assessed using two complementary approaches. For experiments evaluating nitrite production and nitro-oxidative stress, animals were treated with tetrahydrobiopterin (BH4, 100 μM; Sigma-Aldrich, Brazil) or N-acetylcysteine (NAC) 30 min before tissue collection. Intracellular NO levels were evaluated in freshly isolated cardiomyocytes using 4-amino-5-methylamino-2',7'-difluorofluorescein diacetate (DAF-FM diacetate; Invitrogen, Carlsbad, CA, USA). Cells were incubated with DAF-FM diacetate for 30 min at room temperature in the dark, washed with Tyrode's solution to remove excess probe, and immediately imaged using a fluorescence microscope under identical acquisition settings for all experimental groups. Some cardiomyocytes were pre-incubated for 30 min with tetrahydrobiopterin (BH4,100 μM - Sigma-Aldrich Brazil). Fluorescence intensity was quantified using ImageJ software and expressed as an index of intracellular NO production.

NO production was also determined indirectly by measuring nitrite levels using the Griess reaction. Briefly, 100 μL of cardiomyocyte lysate were mixed with 100 μL of Griess reagent (1% sulfanilamide in 2.5% H₃PO₄ and 0.1% N-(1-naphthyl) ethylenediamine dihydrochloride in 2.5% H₃PO₄; 1:1) and incubated for 10 min at room temperature. Absorbance was measured at 490 nm using a microplate reader, and nitrite concentration was calculated from a standard curve generated with sodium nitrite (NaNO₂). To further assess nitro-oxidative stress, myocardial 3-nitrotyrosine levels were quantified using a commercially available sandwich ELISA kit (3-Nitrotyrosine ELISA Kit, Abcam, ab116691), according to the manufacturer's instructions. Protein extracts from isolated cardiomyocytes were loaded into antibody-coated 96-well plates, and absorbance was measured at 450 nm using a microplate reader. The concentration of 3-nitrotyrosine was calculated from a standard curve and normalized to total protein content.

**Western blot analysis**

The complete gels and the Ponceau membranes are provided in the Supplementary Material. To avoid contamination by endothelial proteins, protein expression was assessed in isolated left ventricular cardiomyocytes, as previously described. In a subset of animals, tetrahydrobiopterin (BH4) and N-acetylcysteine (NAC) were administered 30 min before cardiomyocyte isolation to investigate the contribution of oxidative stress to CaMKII oxidation ^18,19^. The isolated cardiomyocytes were homogenized in ice-cold lysis buffer with protease (#P8340, Sigma-Aldrich) and phosphatase inhibitor cocktails (#P0044, Sigma-Aldrich). The lysate was centrifuged for 15 min at 6000g at 4 °C, and protein content was quantified using the Lorry protein assay. 50 μg of protein were separated by SDS/PAGE, transferring to a PVDF Transfer Membrane, 0.45 µm (Thermo Scientific). The membranes were incubated overnight and in a refrigerated environment, with the primary antibodies: anti-CaMKII (1:1000; A9196, ABclonal), anti-Oxi-CaMKII (1:1000, #071387, Millipore), anti-phospho-CaMKII 1:500, #4301561, Sigma Aldrich), anti-eNOS (1:1000; sc-136977, Santa Cruz Biotechnology), anti-peNOSser1177 (1:1000; sc- 12972, Santa Cruz Biotechnology), anti-nNOS (1:1000; sc-8309, Santa Cruz Biotechnology), anti-pnNOSser852 (1:1000; sc-19826, Santa Cruz Biotechnology), and anti-GAPDH (1:3000; sc-32233, Santa Cruz Biotechnology), Anti-NOX2/gp91phox (1:2000; SAB5702937, Sigma-Aldrich), p22phox (1:1000; #27297, Cell Signaling), p47phox (1:1000; #63290, Cell Signaling), NOX4 (1:2000: MA5-32090, ThermoFisher Scientific), Akt (1:500; A11016, ABclonal), Phospho-Akt (Ser473) (1:1000; # 9271,Cell Signaling), alpha-Tubulin (1:1000; #2144, Cell Signaling) .

After the overnight incubation, membranes were further incubated with the appropriate conjugated secondary antibody for 1 h at room temperature. After washing, the membranes were developed using enhanced chemiluminescence for immunodetection (Luminata strong™ - Western HRP substrate, Merck-Millipore) and detected with LAS 4000 equipment (GE HealthCare Life Science) or iBrigt CL1000 (Thermo Fisher Scientific).

eNOS and nNOS coupling was determined as previously described ^18^. The samples of isolated left ventricular cardiomyocytes were homogenized using ice-cold RIPA lysis buffer without DTT. To prepare the samples for analysis, 50 μg of non-boiled protein was mixed with a loading buffer lacking β-mercaptoethanol and then resolved using low-temperature SDS-PAGE (7.5%). As previously stated, the eNOS dimer and monomer signals were detected at 260 kDa and 140 kDa, respectively. The coupling of eNOS was determined based on the dimer/monomer ratio, and protein abundance was quantified using ImageJ software through densitometric analysis. To account for any variations in protein loading, GAPDH was used as a control. All results are derived from at least duplicates.

**Real-Time RT-PCR (RT-qPCR) using SYBR Green**

Gene expression analysis (or target detection) was performed using two-step RT-qPCR with the non-specific fluorescent dye system, SYBR Green. Endocardial left ventricular tissue was collected and total RNA was isolated by TRIZOL^®^ (Invitrogen, CA, USA) and quantified before being subjected to reverse transcription (RT) to synthesize complementary DNA (cDNA). The cDNA was then used as a template for the quantitative Polymerase Chain Reaction (qPCR). Each reaction mixture, typically run in technical duplicates, contained the commercial SYBR Green Master Mix (Applied Biosystems by Thermo Fisher Scientific), specific forward and reverse primers for the target gene were obtained from Integrated DNA Technologies (IDT). Gene expression levels were quantified using the Cycle Threshold (Ct) values, and relative expression was calculated by the 2-ΔΔCt method (or absolute quantification was determined via a standard curve) ^20^. Gene expression was normalized by the housekeeping gene GAPDH.

| Gene | Forward 5’ oligo | Reverse 5’ oligo |
| --- | --- | --- |
| CACNA1c | CTGGGACAGTTTGCTCAAGA | GCGTTCTCCATCTCCTCTATTG |
| SCN5a | CATATTCCTGGGCTCCTTCTAC | TGGAAACGCTTCTCCTTCTC |
| KCNH2 | ACCAGTGACCGGGAGATTATAG | GCTTATACTCAGGCAGCACATC |
| KCND2 | CGCCACATCCTCAACTTCTA | CGGTCCTTGTACTCCTCATAAC |
| kCND3 | GTGGCCATCATGCCCTATTA | GAAGATCCTGAAGACACGGAAG |
| KCNQ1 | CTGGGCTCTGTAGTCTTCATTC | CTCGTTCACCGCATCTTTCT |
| KCNA5 | TGTCTCCCTGGATGTGTTTG ( | GGCTTCTCCTCTTCCTTGATG |
| KCNE1 | GAAGGTTGTGCTCTCCATACTC | CTCAAGTCTGTCAGTGCTGTT |
| KChIP2 | CTGAGGGTCTGGAACAACTCCA | CTCCTCGTTGACAATTCCGCTG |
| SLC8A1 | AGCAGCAGTGATGGTGCTAC | TCCAGGATGGTGATGGTGAA |
| GAPDH | AACAGCAACTCCCACTCTTC | CCTGTTGCTGTAGCCGTATT |

**Statistics**

GraphPad Prism software version 8.0.1 (Boston - USA) was used to conduct statistical and graphical representation Data are expressed as mean ± SD. Data was evaluated using the Shapiro-Wilk test for comparisons between two groups, unpaired Student’s t-test or Mann–Whitney one-tailed test was applied, depending on data distribution. For multiple group comparisons, one-way ANOVA followed by Tukey’s post hoc test was used. In Fig. 1B and Fig. 4F, comparisons were performed using χ² test without Yates’ correction. The number of animals used in each experimental group is indicated as “N”, and the number of cell is indicated as “cell”.

**References**

1.	Souza DS, Marques LP, Costa AD, Cruz JS, Rhana P, Santos-Miranda A, et al. Experimental hypothyroidism induces cardiac arrhythmias and ranolazine reverts and prevents the phenotype. *Life Sciences* 2022;**308**:120945.

2.	Alves IGN, Cruz KML da, Mota CMD, Santana DS de, Gaujac DP, Carvalho VCB de, et al. Experimental hypothyroidism during pregnancy affects nociception and locomotor performance of offspring in rats. *European Journal of Pain* 2013;**17**:1291–1298.

3.	Durço AO, Souza DS, Rhana P, Costa AD, Marques LP, Santos LABO, et al. d-Limonene complexed with cyclodextrin attenuates cardiac arrhythmias in an experimental model of doxorubicin-induced cardiotoxicity: Possible involvement of calcium/calmodulin-dependent protein kinase type II. *Toxicology and Applied Pharmacology* 2023;**474**:116609.

4.	Macedo FN, Souza DS de, Araújo JE dos S, Dantas CO, Miguel-dos-Santos R, Silva-Filha E, et al. NOX-dependent reactive oxygen species production underlies arrhythmias susceptibility in dexamethasone-treated rats. *Free Radical Biology and Medicine* 2020;**152**:1–7.

5.	Kmecova J, Klimas J. Heart rate correction of the QT duration in rats. *European Journal of Pharmacology* 2010;**641**:187–192.

6.	Shioya T. A simple technique for isolating healthy heart cells from mouse models. *J Physiol Sci* 2007;**57**:327–335.

7.	Fredj S, Lindegger N, Sampson KJ, Carmeliet P, Kass RS. Altered Na+ channels promote pause-induced spontaneous diastolic activity in long QT syndrome type 3 myocytes. *Circ Res* 2006;**99**:1225–1232.

8.	Teixeira-Fonseca JL, Souza DS, Conceição MR de L, Marques LP, Durço AO, Silva PL da, et al. In vivo tebuconazole administration impairs heart electrical function and facilitates the occurrence of dobutamine-induced arrhythmias: involvement of reactive oxygen species. *Food and Chemical Toxicology* 2024;**187**:114596.

9.	Santos-Miranda A, Gondim AN, Menezes-Filho JER, Vasconcelos CML, Cruz JS, Roman-Campos D. Pharmacological evaluation of R(+)-pulegone on cardiac excitability: Role of potassium current blockage and control of action potential waveform. *Phytomedicine* 2014;**21**:1146–1153.

10.	Teixeira-Fonseca JL, Santos-Miranda A, Marques I lobo S, Marques LP, Alcantara F, Lima Conceição MR de, et al. Eugenol delays the onset of ouabain-induced ventricular cardiac arrhythmias in guinea pigs. *Basic & Clinical Pharmacology & Toxicology* 2023;**133**:565–575.

11.	Santos-Miranda A, Joviano-Santos JV, Marques ILS, Cau S, Carvalho FA, Fraga JR, et al. Electrocontractile remodeling of isolated cardiomyocytes induced during early-stage hypercholesterolemia. *J Bioenerg Biomembr* 2024;**56**:373–387.

12.	Santos-Miranda A, Joviano-Santos JV, Sarmento JO, Costa AD, Soares ATC, Machado FS, et al. A novel substrate for arrhythmias in Chagas disease. *PLOS Neglected Tropical Diseases* 2021;**15**:e0009421.

13.	Schneider CA, Rasband WS, Eliceiri KW. NIH Image to ImageJ: 25 years of image analysis. *Nat Methods* 2012;**9**:671–675.

14.	Picht E, Zima AV, Blatter LA, Bers DM. SparkMaster: automated calcium spark analysis with ImageJ. *American Journal of Physiology-Cell Physiology* 2007;**293**:C1073–C1081.

15.	Ramachandran J, Peluffo RD. Threshold levels of extracellular l-arginine that trigger NOS-mediated ROS/RNS production in cardiac ventricular myocytes. *Am J Physiol Cell Physiol* 2017;**312**:C144–C154.

16.	Zhang Y, Ding Y, Lu T, Zhang Y, Xu N, McBride DW, et al. Biliverdin reductase-A attenuated GMH-induced inflammatory response in the spleen by inhibiting toll-like receptor-4 through eNOS/NO pathway. *Journal of Neuroinflammation* 2018;**15**:118.

17.	Mosqueira M, Konietzny R, Andresen C, Wang C, H.A. Fink R. Cardiomyocyte depolarization triggers NOS-dependent NO transient after calcium release, reducing the subsequent calcium transient. *Basic Res Cardiol* 2021;**116**:18.

18.	Navia-Pelaez JM, Campos-Mota GP, Araujo de Souza JC, Aguilar EC, Stergiopulos N, Alvarez-Leite JI, et al. nNOS uncoupling by oxidized LDL: Implications in atherosclerosis. *Free Radical Biology and Medicine* 2017;**113**:335–346.

19.	Santana MNS, Souza DS, Miguel-dos-Santos R, Rabelo TK, Vasconcelos CML de, Navia-Pelaez JM, et al. Resistance exercise mediates remote ischemic preconditioning by limiting cardiac eNOS uncoupling. *Journal of Molecular and Cellular Cardiology* 2018;**125**:61–72.

20.	Souza DS, Chignalia AZ, Carvalho-de-Souza JL. Modulation of cardiac voltage-activated K+ currents by glypican 1 heparan sulfate proteoglycan. *Life Sciences* 2022;**308**:120916.
