# Supplementary material for: eNOS uncoupling drives oxidative stress and arrhythmogenic vulnerability in experimental hypothyroidism
Source: Redox Biol. 2026 Oct 20;98:104447. doi: 10.1016/j.redox.2026.104447 (PMC13720376; doi:10.1016/j.redox.2026.104447)
Supplement: Supplementary file 3 [file mmc2.docx]

**Supplementary Result**

**eNOS uncoupling favors excessive reactive oxygen species production and enhances susceptibility to stress induced *in vivo* arrhythmias in experimental hypothyroidism in mice**

* ^$^Diego Santos Souza^1,2^, *Leisiane Pereira Marques^2^, Aimée Obolari Durço^2^, Fabiana da Silva Alcântara^2^, Diego José Belato y Orts^2^, Melissa Tainan Silva Dias^3^, Michael Ramon de Lima Conceiçao^2^, Jorge Lucas Teixeira-Fonseca^2^, Luiz Fernando Santos Matos^1^, Maria Luiza Alarcon Mady Barbosa^1^, Daiara Custódio Santos^1^, Luciano dos Santos Aggum Capettini^3^, Carlos Roberto Prudencio^4^, Danilo Lustrino^1^, Jader Santos Cruz^5^, ^#^Danilo Roman-Campos^2^.

1. Department of Physiology, Federal University of Sergipe, São Cristóvão, Brazil.
2. Department of Biophysics, Federal University of São Paulo, São Paulo, Brazil.
3. Department of Pharmacology, Federal University of Minas Gerais, Belo Horizonte, Brazil.
4. Research Scientist, Immunology Center, Institute Adolfo Lutz
5. Department of Biochemistry and Immunology, Federal University of Minas Gerais, Belo Horizonte, Brazil.

*The authors contributed equally to this work

^#^Corresponding Author: Department of Biophysics, Federal University of Sao Paulo, Botucatu Street, 862, Biological Science Building, Sao Paulo, SP, Brazil.

^$^Co-corresponding Author: Department of Physiology, Federal University of Sergipe. Av. Marechal Rondon, S/N, Rosa Elze, Aracaju, SE, Brazil

E-mail addresses: drcampos@unifesp.br (D. Roman-Campos), santos.dss@outlook.com (D.S. Souza).

**Table 1. T3, T4 and TSH measurements**

|  | **Total T3 (ng/ml)** | | **T4 Free (ng/ml)** | | **TSH (µUI/ml)** | |
| --- | --- | --- | --- | --- | --- | --- |
|  | **Control** | **Hypo** | **Control** | **Hypo** | **Control** | **Hypo** |
| **Mean** | 46.82 | 30.01* | 1.15 | 0.60* | 0.53 | 1.37* |
| **Std. Deviation** | 7.34 | 6.45 | 0.14 | 0.17 | 0.07 | 0.14 |

Data are represented as means ± SD. ⁎P < 0.05, Hypo vs Control. n = 7 animals/group. Unpaired Student’s t-test.

**Table 2. Electrocardiogram parameters in control, hypothyroidism (Hypo) and hypothyroidism plus N-acetyl-l-cysteine (****Hypo + NAC) or tetrahydrobiopterin (Hypo + BH4).**

| **PR (ms)** | | | | |
| --- | --- | --- | --- | --- |
|  | **Control** | **Hypo** | **Hypo + NAC** | **Hypo + BH4** |
| **Mean** | 39.35 | 54.98* | 53.00* | 52.84* |
| **Std. Deviation** | 5.229 | 4.484 | 2.550 | 1.855 |
| **QRS (ms)** | | | | |
| **Mean** | 25.98 | 34.31 | 34.60 | 35.17 |
| **Std. Deviation** | 6.199 | 10.63 | 5.771 | 5.345 |
| **QT (ms)** | | | | |
| **Mean** | 101.1 | 164.6* | 170.1* | 136.2* |
| **Std. Deviation** | 20.22 | 17.03 | 22.32 | 17.20 |
| **QTc (ms)** | | | | |
| **Mean** | 42.96 | 66.20* | 66.19* | 75.66* |
| **Std. Deviation** | 6.514 | 6.849 | 7.924 | 4.979 |
| **BPM** | | | | |
| **Mean** | 476.1 | 353.4* | 344.0* | 334.7* |
| **Std. Deviation** | 50.07 | 87.29 | 36.37 | 21.49 |

Data are represented as means ± SD. BMP: beats per minute. ⁎P < 0.05, Hypo vs Control. n = 6 - 8 animals/group. One-way ANOVA followed by Tukey’s post-test.

**Table 3. Action potential parameters in control, hypothyroidism (Hypo) and control and hypothyroidism plus KN93 (Hypo + KN93)**

| **dV/dt (mV/ms)** | | | | |
| --- | --- | --- | --- | --- |
|  | **Control** | **Hypo** | **Control + KN93** | **Hypo + KN93** |
| **Mean** | 131.9 | 143.9 | 122.4 | 141.6 |
| **Std. Deviation** | 31.26 | 26.74 | 31.47 | 25.96 |
| **Amplitude (mV)** | | | | |
| **Mean** | 100.1 | 96.67 | 104.5 | 104.2 |
| **Std. Deviation** | 12.09 | 15.68 | 18.23 | 13.95 |
| **Resting membrane potential (mV)** | | | | |
| **Mean** | -69.86 | -61.22* | -69.93^#^ | -70.42^#^ |
| **Std. Deviation** | 3.59 | 4.968 | 5.056 | 5.528 |
| **10% of Action Potential Duration (ms)** | | | | |
| **Mean** | 0.8493 | 2.137 | 0.5878 | 2.029 |
| **Std. Deviation** | 0.9518 | 1.909 | 0.1891 | 2.431 |
| **50% of Action Potential Duration (ms)** | | | | |
| **Mean** | 5.533 | 20.51* | 3.686 | 16.47* |
| **Std. Deviation** | 4.854 | 14.48 | 2.356 | 15.48 |
| **90% of Action Potential Duration (ms)** | | | | |
| **Mean** | 35.31 | 110.6* | 32.73 | 65.07* ^#^ |
| **Std. Deviation** | 25.14 | 63.80 | 13.48 | 28,97 |

Data are represented as means ± SD. ⁎P < 0.05. Hypo vs Control. N = 5 animals/group; cell = 19 - 23. One-way ANOVA followed by Tukey’s post-test.

**Supplementary Figure 1**. **Electrical remodeling and potassium current alterations in hypothyroid hearts.** (A) Protocol schematic and representative data showing heart rate changes after saline and combined dobutamine + caffeine infusion (Hypo vs. Control, *P = 0.0008). (B) Cell capacitance (*P = 0.0005). (C–F) Steady-state current (I_ss_) and inward rectifier K⁺ current (I_K1_) densities recorded at different membrane potentials (I_K1_ at −140 mV, −130 mV, and −120 mV, *P < 0.0001; −110 mV, *P = 0.0487; −100 mV, *P = 0.0361), with corresponding activation curves (D, F). Animals per group: n = 6–8 (A) and n = 5 (B–F); cells analyzed: 47–52 (B) and 18–21 (C–F). Data are presented as mean ± SD. An unpaired Student’s t-test was used for panel A and B. Two-way ANOVA followed by Tukey’s post hoc test was applied to panels C and E.

**Supplementary Figure 2. Delayed rectifier and transient outward K⁺ currents remain functionally preserved in hypothyroid cardiomyocytes.** (A–B) Normalized inactivation curves of I_to_ and I_Kdr_ currents, respectively, obtained by standard voltage-clamp protocols (insets). (C–D) Recovery from inactivation of I_to_ and I_Kdr_ currents. Insets show corresponding voltage protocols. Data are expressed as mean ± SD. *P < 0.05. Statistical significance was evaluated by unpaired Student’s t-test to V_50_, Slope and tau - τ. Two-way ANOVA followed by Tukey’s post hoc test was applied where appropriate. The number of animals per group is indicated as N = 5 animals/group; cell = 18-21.

**Supplementary Figure 3. Enhanced NCX current, Hypothyroidism disrupts Ca²⁺ handling and promotes CaMKII-dependent electrical instability.** (A, Ai and Aii) Representative traces and density of I_NCX_ recorded in the absence and presence of Ni²⁺, with difference current obtained by subtraction. Panel A: 40 mV, *P = 0.0148; 50 mV, *P = 0.0169. Panel Ai: no significant differences. Panel Aii: 30 mV, *P = 0.0006; 40 mV, *P = 0.0076; 50 mV, *P = 0.0458. (B) SLC8A1 (NCX1) expression (P = 0.5818). (C) Representative line-scan images of Ca²⁺ transients recorded with Fluo-4 AM in ventricular cardiomyocytes from Control and Hypo groups. Out-of-pacing Ca²⁺ release events are indicated by arrows. (D, E) Quantification of Ca²⁺ transient amplitude (F/F₀; *P = 0.0002) and Ca²⁺ spark frequency (*P = 0.0018). (F) Number of out-of-pacing Ca²⁺ events per 10-s recording (*P = 0.0023). (G–H) Quantification of phosphorylated CaMKII (pCaMKII, *P < 0.0001) and oxidized CaMKII (oxiCaMKII, P = 0.6545) normalized to GAPDH. (I) Representative action potential traces showing delayed afterdepolarizations (DADs) and triggered activity. (J–K) Proportion of normal versus arrhythmic cells and distribution of arrhythmia types in each group, with and without KN-93 treatment. Data are presented as mean ± SD. Mann–Whitney one-tailed test was used for Ca²⁺ spark (E) and out-of-pacing event (F) analyses. The number of animals and cells analyzed were as follows: panels A, Ai, and Aii, n = 5 animals/group, 12–15 cells; panel B, n = 5 animals/group; panels C–F, n = 5 animals/group, 17–20 cells; panels G–H, n = 6 animals/group; panels I–K, n = 5 animals/group, 16–20 cells. Unpaired Student’s t-test was applied for other two-group comparisons. In panel J, comparisons were performed using the χ² test without Yates’ correction.

**Supplementary Figure 4. Mitochondrial ROS production and NOX complex expression are decreased in hypothyroid cardiomyocytes.** (A) Representative fluorescence images showing mitochondrial staining with MitoTracker Green and superoxide detection with MitoSOX Red in ventricular myocytes from Control and hypothyroid (Hypo) groups under basal conditions and after H₂O₂ exposure. (B) Quantification of MitoTracker Green fluorescence intensity (−H₂O₂, *P = 0.0010; +H₂O₂, *P < 0.0010). Scale bar: 20 µm. (C) Quantification of MitoSOX Red fluorescence intensity (−H₂O₂, *P = 0.0004; +H₂O₂, *P < 0.0001; Hypo −H₂O₂ vs. Hypo +H₂O₂, P < 0.0001). (D) Representative immunoblots for NOX4, gp91phox, p22phox, and p47phox, with GAPDH as a loading control. (E–H) Densitometric analysis of the respective protein bands normalized to GAPDH. (I) Nitrite levels and (J) 3-Nitrotyrosine: E, P = 0.1231; F, P = 0.6889; G, P = 0.4606; H, *P = 0.0157; I, *P = 0.019, #P = 0.0010; J, *P = 0.045, #P = 0.001 and 0.0003, respectively. The number of animals and cells analyzed were as follows: n = 5 animals/group, 18–22 cells (A–C); n = 5 animals/group (D–H); J n = 8 -11 animals/group. Data are presented as mean ± SD. *Unpaired Student’s t-test was used for two-group comparisons (E,F,G,H). One-way ANOVA followed by Tukey’s multiple comparisons test was used for comparisons involving more than two groups (I, J), *P < 0.05 vs. Control; #P < 0.05 vs. Hypo.
